# BdERECTA controls vasculature patterning and phloem-xylem organization in *Brachypodium distachyon*

**DOI:** 10.1186/s12870-021-02970-2

**Published:** 2021-04-23

**Authors:** Kaori Sakai, Sylvie Citerne, Sébastien Antelme, Philippe Le Bris, Sylviane Daniel, Axelle Bouder, Angelina D’Orlando, Amy Cartwright, Frédérique Tellier, Stéphanie Pateyron, Etienne Delannoy, Debbie Laudencia-Chingcuanco, Gregory Mouille, Jean Christophe Palauqui, John Vogel, Richard Sibout

**Affiliations:** 1 Institut Jean-Pierre Bourgin, INRAE, AgroParisTech, Université Paris-Saclay, 78000 Versailles, France; 2 INRAE, UR BIA, F-44316 Nantes, France; 3 United States Department of Energy Joint Genome Institute, Berkeley, California 94598 USA; 4 Université Paris-Saclay, CNRS, INRAE, Univ Evry, Institute of Plant Sciences Paris-Saclay (IPS2), 91405 Orsay, France; 5 Université de Paris, CNRS, INRAE, Institute of Plant Sciences Paris-Saclay (IPS2), 91405 Orsay, France; 6 USDA-ARS Western Regional Research Center, 800 Buchanan St., Albany, CA 94710 USA; 7 University of California, Berkeley, CA USA

**Keywords:** Brachypodium, ERECTA, Hormones, Phloem, Xylem

## Abstract

**Background:**

The vascular system of plants consists of two main tissue types, xylem and phloem. These tissues are organized into vascular bundles that are arranged into a complex network running through the plant that is essential for the viability of land plants. Despite their obvious importance, the genes involved in the organization of vascular tissues remain poorly understood in grasses.

**Results:**

We studied in detail the vascular network in stems from the model grass *Brachypodium distachyon* (Brachypodium) and identified a large set of genes differentially expressed in vascular bundles versus parenchyma tissues. To decipher the underlying molecular mechanisms of vascularization in grasses, we conducted a forward genetic screen for abnormal vasculature. We identified a mutation that severely affected the organization of vascular tissues. This mutant displayed defects in anastomosis of the vascular network and uncommon amphivasal vascular bundles. The causal mutation is a premature stop codon in *ERECTA,* a LRR receptor-like serine/threonine-protein kinase. Mutations in this gene are pleiotropic indicating that it serves multiple roles during plant development. This mutant also displayed changes in cell wall composition, gene expression and hormone homeostasis.

**Conclusion:**

In summary, ERECTA has a pleiotropic role in Brachypodium. We propose a major role of ERECTA in vasculature anastomosis and vascular tissue organization in Brachypodium.

**Supplementary Information:**

The online version contains supplementary material available at 10.1186/s12870-021-02970-2.

## Introduction

Formation of vasculature was a critical step in plant evolution that allowed plants to colonize the land. The vasculature forms an interconnected network that transports water and photosynthate throughout the plant [1]. Vascular ontogeny and patterning has been intensively studied in dicotyledonous [2, 3] and less in monocotyledonous plants [4–6]. Vascular strands are initiated from primary meristems in which a precise orchestration of hormonal mechanisms control both phyllotaxy and patterning of vascular bundles (VB) [7]. Both dicotyledonous and monocotyledonous plants have the same basic primary vascular plan consisting of large acropetally differentiating veins, however, grass stems are characterized by reiterative phytomers that need to be connected together along the stem for proper hydraulic supply. The inner organization of the VB varies between species and tissues and can be organized in different patterns: collateral (xylem and phloem are located on two distinct poles with phloem at a distal position), amphicribal (xylem is surrounded by phloem) or amphivasal (phloem is surrounded by xylem). Collateral is the pattern observed in most plants. Interestingly, organization of conductive elements diverged during evolution of angiosperms. For instance, when grass vasculature is formed, it lacks the thickening of vascular tissues produced by secondary meristem, the cambium located in an open collateral VB which separate xylem and phloem active in most dicotyledonous plants [8]. Whether dicotyledonous plants gained or grasses lost this ability remains open to debate [9] but, importantly, precise vascular patterning is crucial for monocotyledonous plants because they lack the cambial layer that allows some dicots to adjust their water conductance during their life through the formation of wood. The molecular and genetic underpinnings that drive vascular patterning are understudied in the grasses compared with dicots like *Arabidopsis* *thaliana* (Arabidopsis) and poplar although a few characterized genes, notably related to hormone homeostasis impact vascular patterning in rice and in maize [6, 10–15]. *Brachypodium distachyon* (Brachypodium) is cited as a relevant model plant for cell wall studies [16–18], but to date, it has not been extensively used for the identification of genes responsible for vasculature development despite a published description of its vascular anatomy [19]. Recently, abnormal vasculature development was observed by [20] when MAP20, a gene involved in vessel pit formation was knocked down in Brachypodium. In the current paper, we show that two alleles of an ortholog of Arabidopsis *ERECTA* induce fusion of vascular bundles (called anastomosis) and abnormal patterning in internodes of Brachypodium, a phenotype not reported in other grass *erecta* mutants. While Arabidopsis ERECTA is recognized as a developmental master gene which tune tissue elongation and plant architecture by activating the brassinosteroid and auxin signaling pathways [21–23] much less is known about its role in grasses. The rice OsERECTA1 was previously shown to be involved in heat tolerance [24] and very recently, to be a negative regulator of spikelet number per panicle through the indirect activation of a cytokinin oxidase [25]. In the Brachypodium ERECTA mutants, hormone homeostasis is disrupted and some VB lack polarity, displaying amphivasal phenotype. In this work, we use laser capture microdissection (LCM) to discover genes mainly expressed in nascent VB and to localize *BdERECTA* in the apical meristem. These results suggest that the Leucine-Rich Repeat Receptor Like Kinase (LRR-RLK) BdERECTA is a key factor controlling the anastomosis of vascular tissue in Brachypodium.

## Results

### Vascular network in the Brachypodium stem displays a regular pattern

**Fig. 1:**
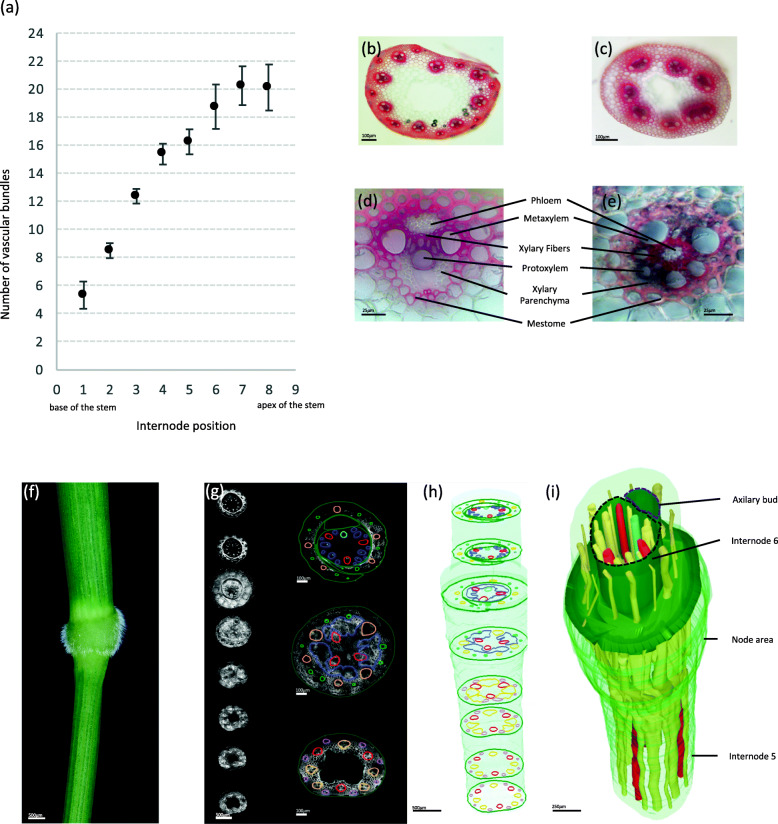
Pattern of vascular bundles along the main floral stem of Brachypodium. **a** Number of vascular bundles (VB) along the main primary stem of Brachypodium (*n* = 7). All VBs displayed collateral shape as described in **d** except in the first internode in which 20% of them were fused with at least one other VB and 14% displayed amphivasal phenotype as showed in **e**. **b** Hand-cross section in the fifth internode of WT Brachypodium stem at 45 Days After Germination (DAG). **c**, Hand-cross section in the first internode of WT stem at 45 DAG. **d**, Large collateral VB from the fifth internode of Brachypodium stem at 45 DAG. (e) VB with amphivasal phenotype in the first internode of WT Brachypodium stem at 45 DAG. All sections were stained with phloroglucinol-HCl. **f** Fifth node was used for making cross sections that were then imaged with a confocal microscope. **g** Eight cross sections illustrate a series of 36 sections (repeated in three different samples) imaged with a confocal microscope (left) and illustration of how VBs were manually identified in different area (right). Within the internode, all VB are distinct from each other. Most small VBs that are represented in pink in the internode are recognizable in the upper leaves as future small veins (green circles). Large vascular bundle colored in orange in the internode are interconnected together in the node (dark blue) and give new VB in the upper internode. Three vascular bundles (red) cross the node without any visible anastomosis. A new vascular bundle (light blue) develops in the node, in front of the axillary bud. **h** Illustration of stacked typical cross sections in the reconstructed node. **i** Three-dimensional reconstruction of the internode-node area (748 confocal images were produced for 3D reconstruction). In this reconstruction, VBs are shown in yellow while the three vascular bundles that do not anastomose in the node are shown in red and the neo-formed VB is shown in blue. Each vascular bundle was identified manually with the FreeD software

To characterize the vascular network in Brachypodium stems, we made cross-sections one centimeter below the top of each internode (numbered 1–9 starting at the base) along the main stem of seven plants at the flowering stage and when the stem was fully elongated (Fig. 1). The sections were stained with phloroglucinol-HCl which stains lignified tissues red; thus, highlighting the VBs. The number and size of VBs varied across the stem (Fig. 1a) while the number of VBs was stable inside each internode. Indeed, the number of VBs increased from four to seven at the base of the stem (first internode) to an average of 20 in the peduncle.

The typical stem VB in internode contains two large metaxylem vessels separated by smaller connecting xylem cells (Fig. 1b, d). However, more metaxylem cells can be observed when sections are made close to the nodes. Connecting xylem cells are conductive elements separated from phloem cells by a few layers of highly lignified xylary fibers. Protoxylem vessels can be observed below the connecting xylem cells. These elements display annular lignified thickenings. The protoxylem vessels are adjacent to xylem parenchyma cells. Phloem cells are located distal to the protoxylem cells. The entire VB is surrounded by a layer of sclerenchyma tissue called mestome in C3 grasses [26, 27]. In the first internode (at the base of the stem) VBs have a different pattern inside the stem (Fig. 1c) and different shape (Fig. 1e). Indeed, we observed that among VBs present in the first internode, some VBs were anastomosed or displayed amphivasal shape with phloem tissues in the middle of the VB while xylem vessels were located outward the VB (Fig. 1e). Among seven different plants, 20% of VBs were fused with another VB and 12.5% showed amphivasal shape in the first internode while these phenotypes were never observed in upper internodes.

**Fig. 2:**
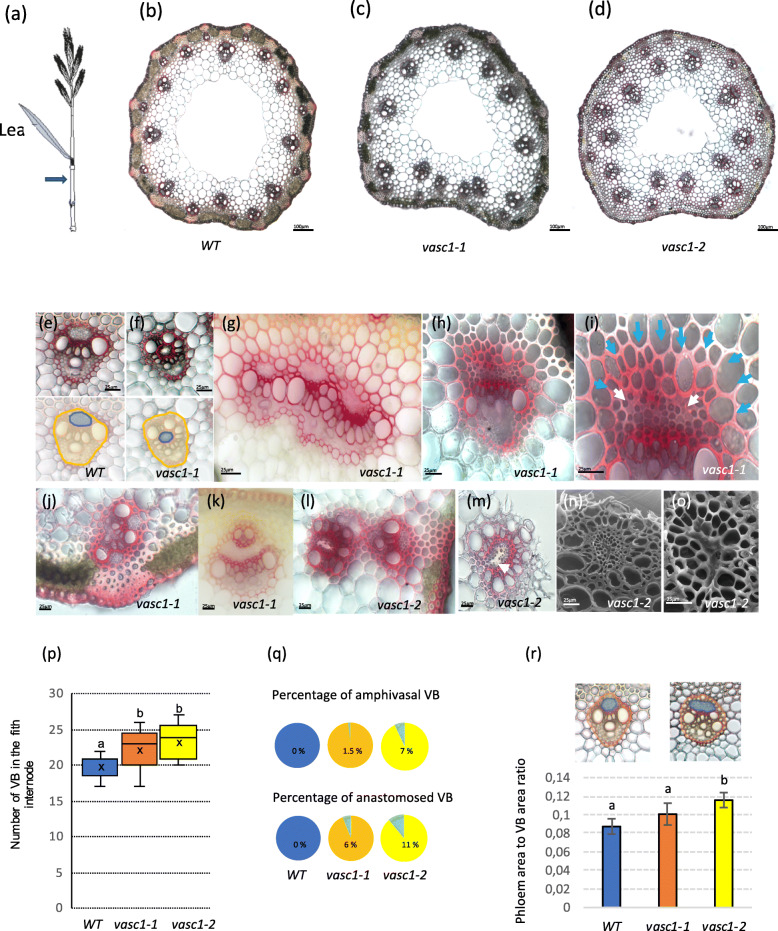
Vascular phenotype of *vasc1–1 and vasc1–2*. **a** Illustration of spike (including peduncle), last leaf, last node and penultimate internode. Blue arrow shows where cross sections were made. **b** Cross sections in WT stem. **c** *Vasc1–1*. **d** *Vasc1–2*. **e** Vascular bundle (VB) of WT. VB and phloem area from WT are delineated in orange and blue, respectively (bottom). **f** Amphivasal VB in *vasc1–1*. VB and phloem area from *vasc1–1* are delineated in orange and blue respectively (bottom). **g** Three anastomosed VBs in internode from *vasc1–1*. **h** VB from *vasc1–1*. **i** Magnification of **h** in the phloem area. White arrows show abnormal phloem cells. Blue arrows show enlarged lignified mestomes cells adjacent to phloem area. **j**, Two anastomosed VBs in *vasc1–1*. **k** Small VB inserted into the phloem tissues of large VB (*vasc1–1*). **l** Misplaced VB in *vasc1–2*. **m** VB in *vasc1–2*. Arrow show abnormal phloem tissues. **n** Scanning Electron Microscopic picture of **m**. **o** Focus on phloem area of (n). All sections were stained with phloroglucinol-HCl except **n** and **o**. **p** Number of VBs in WT and mutants penultimate internodes as shown in **b**. Letters above histograms indicate significant differences (*p* < 0.05) between genotypes after Tuckey’s pairwise test (*n* = 17). **q** Percentages of amphivasal or anastomosed VBs in the WT, *vasc1–1*, *vasc1–2*. (r) Ratio of phloem area versus the whole VB area in typical collateral shaped VBs in WT, *vasc1–1*, *vasc1–2*. Letters above histograms indicate significant differences (*p* < 0.05) between genotypes after Tuckey’s pairwise test (*n* = 6 plants, each section comprised from 17 to 24 VBs)

To gain a comprehensive view of the vascular network in the node-internode transition, we made 36 cross sections (100 μm each) from 1 cm below the fifth node to the top of it and repeat this experiment in three different nodes. We subsequently stacked 748 confocal images from one node to reconstruct the vascular network in 3D (Fig. 1f-i). As stated above VBs in the internode are not connected until they converge into the node because we clearly observed alternating large and small VBs with the latter in the most distal area of the internode sections (Fig. 1g, h, Fig. 2b, Fig. S1). Most VBs anastomosed in the node. This phenomenon is easily visible as most VBs enlarge in the node region (Fig. S1). However and despite anastomosis complicating the identification of the vascular network in the node, we noticed that three VBs (in red, Fig. 1h, i and Fig. S1) cross the node without any apparent anastomosis with other vessels because these three VBs do not enlarge in the node as observed for the five other VBs (Fig. S1). Anastomosis allows VBs to continue up the stem to the upper internode or branch into the leaf sheath (delineated in green, Fig. 1 g, h, i). Moreover, a new VB (light blue, Fig. 1h, i) develops in the node adjacent to the lateral bud.

### *BdERECTA* controls vascular bundle development in Brachypodium

In order to identify genes involved in vascular organization, we carried out a forward genetic screen on a population of 300 M2 chemically-induced Brachypodium mutants [28]. We made hand-cross sections in the top part of the internode below the peduncle and stained them with phloroglucinol-HCl in order to visualize the lignin in the walls of metaxylem cells, intra-vascular fibers and mestome cells in VBs [29, 30].

Among the progeny of one mutagenized line, named *vasc1–1*, some plants showed an obvious irregular vascular bundle pattern in internodes (Fig. 2). While most wild-type (WT) internodes display alternate arrangement of large and small bundles in a circular pattern around the stem (except in the first internode) (Fig. 2a, b), the mutant displayed misplaced bundles with large bundles occasionally located in the proximal area (Fig. 2c), an organization never observed in WT. In addition, we also occasionally observed amphivasal VBs (Fig. 2e, f) and fusions of large VBs together (Fig. 2g and j) and rarely, complete embedding of small VBs into the phloem tissues of large VBs (Fig. 2k). The organization of tissues in some VB were drastically disturbed in *vasc1–1*. For instance, phloem sieve elements were often collapsed and the mestome cells adjacent to the phloem area were oversized compared to mestome in WT (Fig. 2h, i). Again, these phenotypes were never observed in the WT internodes above the first internode.

**Fig. 3:**
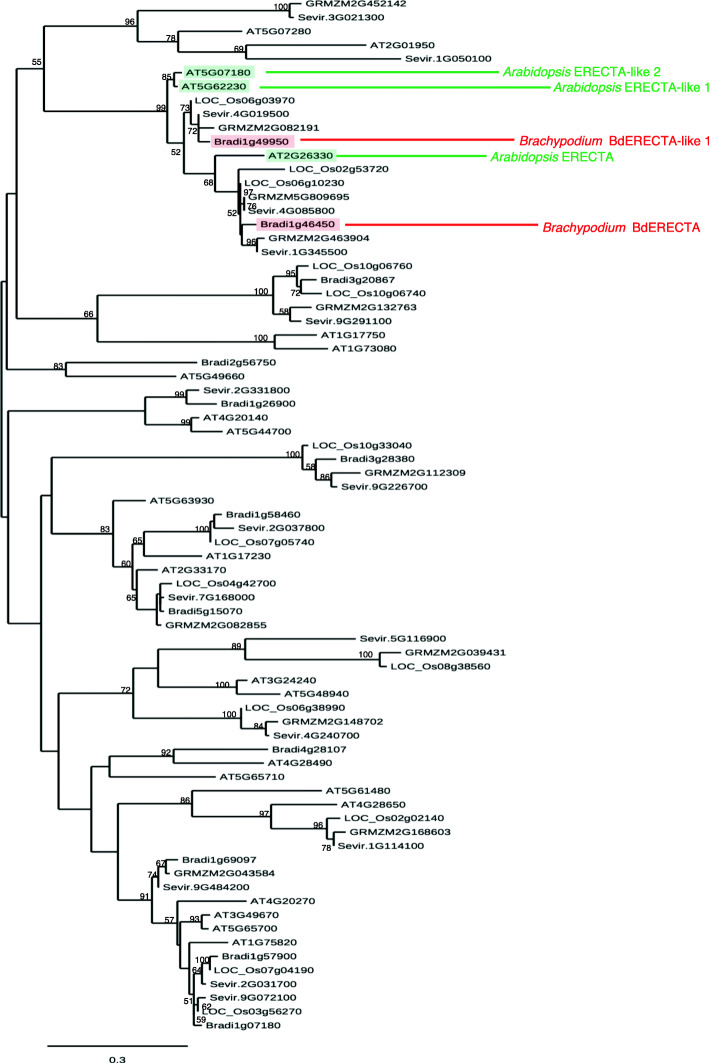
Neighbor-joining phylogeny tree of Leucine-Rich-Receptor kinase proteins from *Brachypodium distachyon*, *Oryza sativa, Setaria veridis, Zea mays and Arabidopsis thaliana*. The tree was made with proteins sharing more than 35% amino acid identity with Arabidopsis ERECTA (At2g26330, Table S2). Node value are bootstrap values. Legend shows the number of substitution per kb

Homozygous *vasc1–1* plants were nearly sterile but by examining the progeny of the heterozygous M2 line, we noted that the phenotype was recessive and segregated with a typical 3:1 ratio suggesting that it was induced by a single mutation. We sequenced pooled DNA from 16 M2 and M3 plants showing the mutant vascular phenotype. We found a total of 1377 mutations of which only four were homozygous (Table S1). Three of the mutations were located in intergenic regions or an intron and are therefore unlikely to be the causal mutation (Table S1). The fourth homozygous mutation was of particular interest because it introduced a nonsense mutation 1026 bp from the predicted start codon of the gene Bradi1g46450 that encodes a Leucine-Rich Repeat Receptor-Like Kinase (https://phytozome.jgi.doe.gov/). This mutation induces a premature stop codon early in the N-terminal region of the protein and only allows the translation of 20 amino acids (aa) instead of the predicted 978 aa in WT (Fig. S2). This suggests that the mutation is a complete knockout. Bradi1g46450, encodes a Leucine-Rich Repeat Receptor Like Kinase with 85% similarity to the Arabidopsis ERECTA protein. Interestingly, it has been shown that ERECTA is involved vasculature cambium maintenance in Arabidopsis [31, 32]. A survey of the Brachypodium Leucine-Rich Repeat Receptor Like Kinases (LRR-RLKs) in the Brachypodium genome showed that Bradi1g46450 is the ortholog of the Arabidopsis ERECTA protein (At2g26330). Thus, we named Bradi1g46450, *BdERECTA*. Another gene, Bradi1g49950, that we named *BdERECTA-like 1* is a close paralog (Fig. 3, Table S2). There are three homologous proteins in *Setaria veridis*, *Zea mays, Oryza sativa and * Arabidopsis (Fig. 3)*.* To definitively validate this allele of Bradi1g46450 is responsible for *vasc1–1* phenotype, we selected and genotyped a mutant, *vasc1–2,* harboring a T-DNA insertion [33] in the coding sequence of Bradi1g46450 (Fig. S2). The T-DNA is inserted in the first intron of Bradi1g46450, 299 bp after the start codon. *Bradi1g46450* transcript was not detected in mature and fully elongated stems (45 DAG) of *vasc1–2*, confirming that this T-DNA mutant is a null allele of *Bradi1g46450* (Fig. S2). Not surprisingly, *Bradi1g46450* transcript was detected in *vasc1–1* (a chemical-induced mutant) albeit at a low level compared to WT (Fig. S2). *vasc1–2* also clearly displayed vascular defects. We found that both mutants had significantly more VBs in the penultimate internode located below the peduncle (Fig. 2b-d, p) and that a substantial proportion of them were misplaced in the stem and showed amphivasal shape or were anastomosed (Fig. 2a-d, l, p, q). As observed in *vasc1–1*, scanning electron microscopy of *vasc1–2* confirmed abnormal shape of phloem and sieve elements (Fig. 2m-o). We quantified phloem area in *vasc1–1*, *vasc1–2* and WT in VBs showing a typical collateral pattern similar to WT and consequently by excluding anastomosed or amphivasal VBs. Relative to the entire VB area, phloem area were larger in *vasc1–2* and *vasc1–1* compared to WT but only, *vasc1–2* displayed significant statistical difference (Fig. 2r).

**Fig. 4:**
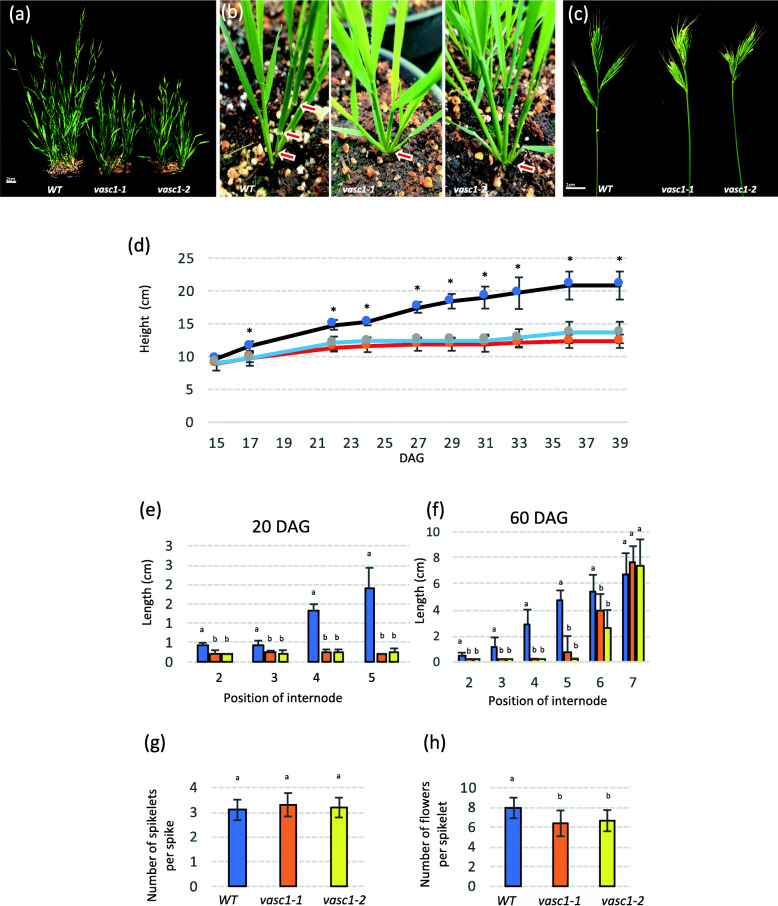
Phenotype of *vasc1–1* and *vasc1–2*. **a** *vasc1–1* and *vasc1–2* mutants are shorter than WT at 45 Days After Germination (DAG). **b** Both mutants are severely affected in internode elongation prior flowering (20 DAG). Red arrows show position of visible nodes. **c** Spikelet development is affected in both *vasc1–1* and *vasc1–2*. **d** Growth curve (height) of mutants (red line, *vasc1–1*; blue line, *vasc1–2*) and WT (black line) from 15 to 39 DAG. Stars show significant differences (one way ANOVA, *p* < 0.01, *n* = 10). **e** Internode size at 20 DAG (*n* = 10). **f** Internode size at plant maturity (60 DAG, *n* = 10). **g** Number of spikelets per spike at 50 DAG (*n* = 23 spikes). **h** Average number of flowers per spikelet at 50 DAG (*n* = 64). Letters above histograms indicate significant differences (*p* < 0.05) between genotypes after Tuckey’s pairwise test

Both *vasc1–1* and *vasc1–2* were shorter compared with WT (Fig. 4a, d) and this was due to reduced internode elongation (Fig. 4b). This phenotype was quantified at vegetative stage (20 DAG, Fig. 4e) and at mature stage (when fully elongated stem were drying, 60 DAG, Fig. 4f). This revealed that the upper internodes were not affected as much as the lower internodes. Spikes were abnormally shaped in both mutants (Fig. 4c). While number of spikelet per stem was similar both in mutants and WT, we observed less flowers per spikelets in *vasc1–1* and *vasc1–2* (Fig. 4g, h). Both mutants showed severe sterility because only 29 and 3 viable seeds were found among 123 *vasc-1-1* and 146 *vasc1–2* lines respectively, while each WT plant produces between 100 and 300 seeds in our culture conditions.

### *BdERECTA* is expressed in both vascular bundles and parenchyma tissues

**Fig. 5:**
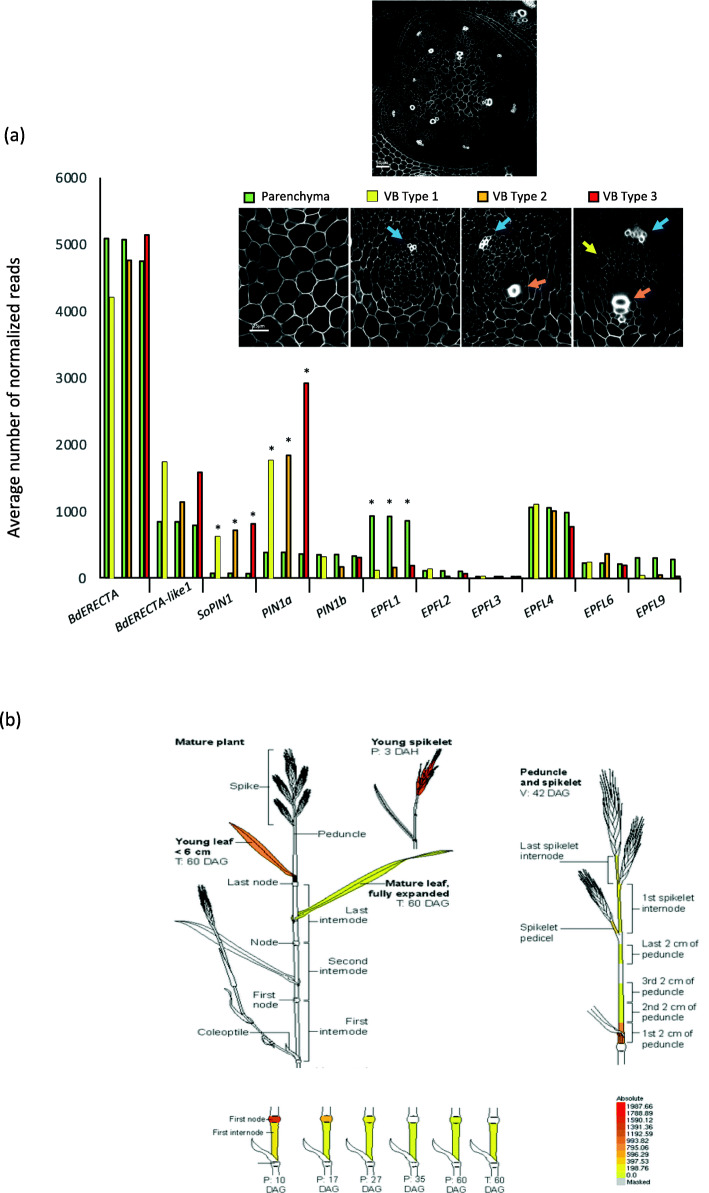
Expression pattern of *BdERECTA* in Brachypodium. **a** Expression level of some selected genes in three different types of vascular bundles (VBs) and in parenchyma from shoot apex. Pictures display confocal images highlighting how different types of VB where selected (see Fig. S3): VB Type 1, no protoxylem cell (with thick cell wall) is detectable in the laser-micro-dissected VBs; VB Type 2, only one or two protoxylem cells are detectable in the laser-micro-dissected VBs; VB Type 3, three protoxylem cells are detectable in the laser-micro-dissected VBs. *BdERECTA* (Bradi1g46450.1), *BdERECTA-like1* (Bradi1g49950.1), *SoPIN* (Bradi4g26300.2), *PIN1a* (Bradi1g45020.1), *PIN1b* (Bradi3g59520.1), *EPFL1* (Bradi3g38740.1), *EPFL2* (Bradi1g10080.1), *EPFL3* (Bradi2g05865.1), *EPFL4* (Bradi1g74380.1), *EPFL6* (Bradi2g22340.1), *EPFL9* (Bradi2g58540.1). Blue arrows refer to protophloem cells. Red arrows refer to protoxylem cells. Yellow arrow shows nascent metaxylem. Stars show significant values (false discovery rate, see Table S3). **b** In silico expression level of *BdERECTA* using eFP browser in some sampled organs of *Brachypodium distachyon* (Sibout et al., 2017). Expression values of *BdERECTA* transcript are indicated by a colour gradient (bottom right), where yellow indicates no detectable expression while red signifies highest expression

In order to identify genes involved in vascular tissue formation and quantify *BdERECTA* expression in nascent VB, we conducted laser capture microdissection (LCM) and RNAseq analysis on three different types of vascular bundles. Vascular procambium cells originate from a region close to the shoot apical meristem and increase in number as VBs mature. Therefore, in order to capture different developmental stages of VBs, we selected VBs according to number of visible protoxylem cells from serial cryo-sections of shoot apical area at 20 DAG (before flowering, Fig. S3). We used parenchyma tissue micro-dissected from the center of the stem as a control (Fig. S3, Fig. 5). Since procambium have little chlorophyll at very early stages we used this feature to detect nascent VBs in the shoot apex (Fig. S3b, c). In VB type 1 (without any detectable protoxylem cells (Fig. S3 and Fig. 5), we found 541 genes differentially expressed with 286 genes up-regulated (70 genes were exclusively detected in VB1 and not detected in parenchyma cells) in VB versus parenchyma (Table S3). In VB type 2 (showing one or two detectable protoxylem cells Fig. S3 and Fig. 5), we found 495 genes differentially expressed with 175 genes up-regulated (35 were exclusively detected in VB2 and not detected in parenchyma cells) in VB versus parenchyma (Table S3). In VB type 3 (showing three detectable protoxylem cells Fig. S3 and Fig. 5), we found 614 genes differentially expressed with 179 genes up-regulated (25 were exclusively detected in VB3 and not detected in parenchyma cells) in VB compared to parenchyma (Table S3). However, when we compared all VB transcriptomes together to parenchyma tissue only, we found 1769 gene differently expressed between these two types of tissues (Table S4). Many orthologs of genes recently shown to be involved in vascular differentiation and in xylem/phloem differentiation in Arabidopsis [34, 35] displayed high expression level in VBs. For instance, Bradi1g26570, an ortholog of TMO6 (At5g60200) was four fold up-regulated in VBs versus parenchyma and a PXY ortholog (Bradi3g17567, At5g61480) was three to five fold up-regulated in VB (according to VB type) compared to parenchyma. To definitely confirm that our LCM experiment discriminates correctly VB from parenchyma tissues, we checked for the expression pattern of some genes known to be specifically expressed in grass VBs and already published. Xylem cysteine proteinase (XCP1, Bradi2g39320) is the second most differentially expressed gene in our RNAseq data with 30 fold higher expression level in VBs versus parenchyma. pXCP1:XCP1::gus lines indicated specific expression of *XCP1* in protoxylem and metaxylem cells in the apical shoot area (Fig. S4) as previously observed in stem by [36]. Similarly, *PIN1a* transcript is 10 and 16 fold higher represented in VB type1 and VB type 3 respectively compared with parenchyma. PIN1a-citrine [10, 37] was clearly and specifically located in VBs of apical shoot area confirming *PIN1a* is differentially expressed in VB compared to parenchyma (Fig. S4). These results confirmed our LCM samples are clearly enriched for VBs. *BdERECTA* was highly expressed in all tissues with no significant differences (Fig. 5a). Interestingly, we observed that *BdERECTA-like 1* was more highly expressed in VBs than in parenchyma, however, this difference was not statistically significant. Because Arabidopsis ERECTA interacts with different ligands depending of localization in the plant [38], we focused our attention on orthologs of *EPIDERMAL FACTOR-LIKE i.e* EPFL1, 2, 3, 4, 6 and 9 transcripts (Fig. 5a). Among the latter, *BdEPFL1* (also named RAE in rice, Os08g0485500, [39]) was significantly more expressed in parenchyma tissues than in VB at all stages. *EPFL9* displayed the same pattern of expression as *EPFL1*, but the pattern was not statistically significant.

At a macroscopic scale, *BdERECTA* is expressed in young tissues including leaves, spikes, nodes and in the base of internodes (Fig. 5b) according to BAR (https://bar.utoronto.ca/efp_brachypodium/cgi-bin/efpWeb.cgi and PlaNet (https://aranet.sbs.ntu.edu.sg/responder.py?name=gene!bdi!5672) databases [40].

According to the data in PlaNet, *BdERECTA* is co-expressed with several hormone responsive genes (SAUR genes for example), genes involved in shoot apical meristem development and vascularization (CLAVATA1 and YABBY homologs [41, 42]), and cell wall biosynthetic genes (e.g. xyloglucan galactosyltransferase, cellulose synthase-like) (Table S5) (https://aranet.sbs.ntu.edu.sg/responder.py?name=gene!bdi!5672 [40]. Interestingly, we discovered that *BdERECTA-like 1* is among the 137 genes co-expressed with *BdERECTA*.

### Microarray analysis of *vasc1–1* reveals changes in vasculature and hormone metabolism

We carried out microarray analysis on 20 DAG plants. At this stage, the plants have not begun to flower and the visual phenotype of *vasc1–1* is not easily discernible from WT (Fig. S5). We chose this stage in the hopes of capturing more primary transcriptomic responses rather than indirect developmental responses that may occur later due to the loss of BdERECTA activity. Because *vasc1–1* is a chemically-induced mutant with multiple mutations, we compared transcriptomes of four homozygous *vasc1–1* plants with four plants heterozygous for the *vasc1–1 BdERECTA* mutated allele*.* We found 215 and 541 genes differentially expressed with the Bonferroni or Benjamini-Hochberg false discovery methods, respectively (Table S6).

As observed by RT-PCR on mature stems (Fig. S2), microarray analysis confirmed a lower *BdERECTA* transcript level in homozygous *vasc1–1* compared to heterozygous *vasc1–1* at this stage. Regarding the vascular phenotype, we noticed that *Bradi3g10270* and *Bradi4g07570* which are the orthologs of the specific phloem sucrose transporters, *Arabidopsis sweet 7* (*At4g10850*) and *11* (*At3g48740*) were overexpressed (1.5 fold) in *vasc1–1* homozygous mutant (Table S6). Similarly, the transcript level of *Bradi2g0025*, an ortholog of a phloem specific protein PP2 in Arabidopsis (*At4g19840*) was also found 1.45 fold more abundant in homozygous *vasc1–1* at 20 DAG compared to heterozygous plants. The hypothesis of a role of Brachypodium ERECTA in xylem differentiation and proliferation is reinforced by the downregulation of *Bradi3g13291* (1.5 fold lower expression in homozygous *vasc1–1* versus heterozygous), an ortholog of tracheary element differentiation-related 7 (TED7, *At5g48920*) involved in tracheary development in *Zinnia elegans* [43] (Table S6). At last, genes putatively related to sugar metabolism were found to be misregulated in the mutant (Table S6). In general, a very small set of genes involved in hormone biosynthesis or homeostasis was deregulated at 20 DAG. For instance, expression of *Bradi2g06030*, an ortholog of a cytokinin oxidase in Arabidopsis (*CKX5*) and rice (*OsCKX2*) was decreased by 1.35 fold at 20 DAG (Table S6) suggesting that turnover of cytokinin is impacted in *vasc1–1*. This result is in line with the very recent discovery that rice ERECTA1 indirectly controls the expression of OsCKX2 [25]. In contrast, homologs of ethylene forming enzyme (EFE) encoding a aminocyclopropane-1-carboxylate oxidase 4 (*Bradi4g31820*/ *At1g05010*) and the protein phosphatase 2C (PP2C, *Bradi2g54810*/ *At2g2938*0) involved in ABA signaling were overexpressed (1.5 fold, Table S6). We did not detect altered expression of genes involved in auxin biosynthesis or degradation at 20 DAG but typical auxin-responsive genes like PILS1 (*Bradi3g60740*/ a homolog of *At1g20925*) or Small Auxin-Upregulated RNA (SAUR16, *Bradi3g1389*/ a homolog of *At4g38860*) were upregulated (1.5 and 2 fold respectively in homozygous *vasc1–1*, Table S6). Importantly, *BdERECTA-like1* was not deregulated in *vasc1–1* in homozygous plants compared to heterozygous plants (Table S6)*.* This suggests that the phenotype of *vasc1–1* is not mediated by changes in *BdERECTA-like 1* expression.

### Hormone homeostasis is altered in *vasc1–1*

**Fig. 6:**
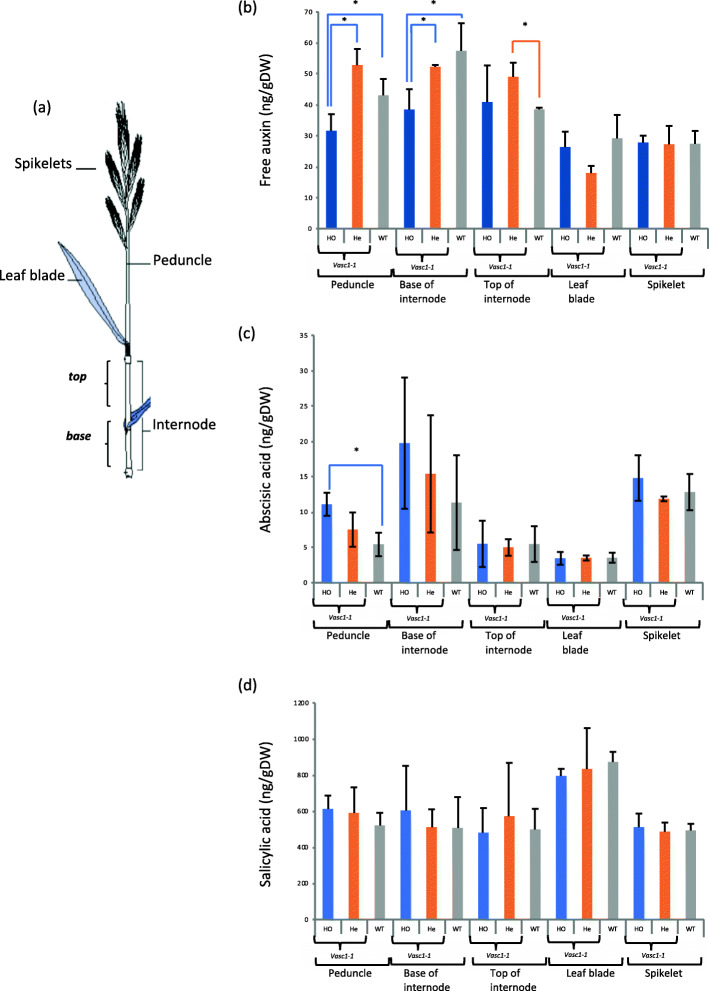
Free auxin content, abscisic and salicylic acid content *in vasc1–1*. Hormone levels in different organs of 42-DAG *vasc1–1* homozygous plants (*vasc1–1* ^*ho*^), *vasc1–1* heterozygous plants (*vasc1–1* ^*he*^) and WT plants. **a** Scheme of the different organs sampled for quantification of hormone content. **b** Free auxin content. **c** Abscisic acid content. **d** Salicylic acid content. Error bars are standard deviations. Stars show significant differences between genotypes (ANOVA, *p*-value< 0.05, *n* = 3)

**Fig. 7:**
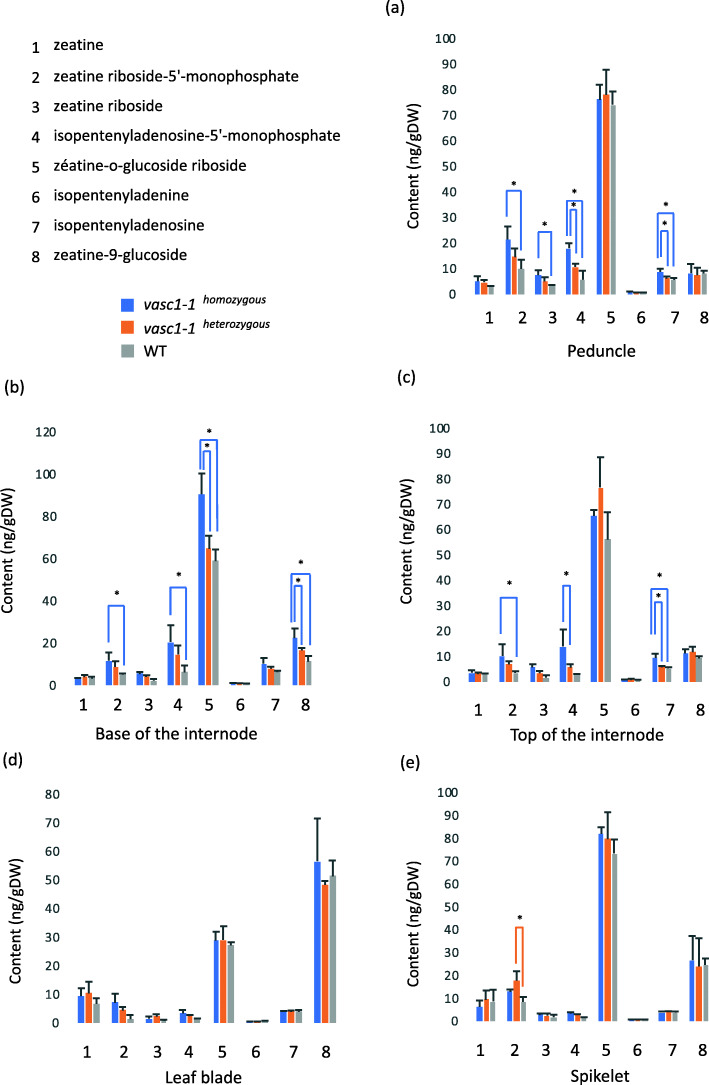
Cytokinin content in *vasc1–1*. Cytokinins were quantified in different organs (see Fig. 6a) at 42 DAG. **a** Peduncle. **b** base of internode. **c** Top of the internode. **d** Leaf. **e** Spikelet. Error bars are standard deviations. Stars show significant differences between genotypes (ANOVA, p-value ≤0.05, *n* = 3), *vasc1–1* homozygous plants (*vasc1–1* ^*ho*^), *vasc1–1* heterozygous plants (*vasc1–1* ^*he*^) and WT plants

The altered shoot development of *vasc1–1* and *vasc1–2* as well as the misregulation of hormone homeostasis-related genes observed at 20 DAG and the co-expression of hormone homeostasis-related genes with BdERECTA observed in the PlaNet database (https://aranet.sbs.ntu.edu.sg/responder.py?name=gene!bdi!5672) led us to quantify hormone content in different tissues of mature WT and *vasc1–1* plants with fully elongated stems at 42 DAG (Figs. 6 and 7). We quantified hormones in WT and, to control for background mutations, heterozygous and homozygous *vasc1* mutants. We used the entire peduncle, the base and the top of the last true internode, leaves and spikelets (Fig. 6a). The *vasc1–1* mutant showed significant reduction of free auxin content in the entire peduncle and at the base of the internode when compared with WT and heterozygous mutant (Fig. 6b). This difference was not found at the top of the internode despite heterozygous *vasc1–1* was found significantly different compared to WT. In leaves and spikelets, auxin content was detected but no significant differences were found (Fig. 6). While ABA levels tended to be higher in homozygous *vasc1–1* plants, no significant difference were found between homozygous or heterozygous *vasc1–1* or WT in the different tissues, except in the peduncle where ABA content in homozygous *vasc1–1* was higher than WT but not heterozygous *vasc1–1* (Fig. 6c). Similarly, no difference was found in salicylic acid content in all genotypes (Fig. 6d).

We quantified the level of eight different cytokinins (Fig. 7). As observed for auxin content, leaves and spikelets did not display major changes in cytokinins levels. However, in contrast to free auxin content, amounts of cytokinins were increased in the peduncle and in the internodes of homozygous *vasc1–1* compared with WT.

### Lignocellulosic content is impacted in *vasc1–1* and *vasc1–2*

**Fig. 8:**
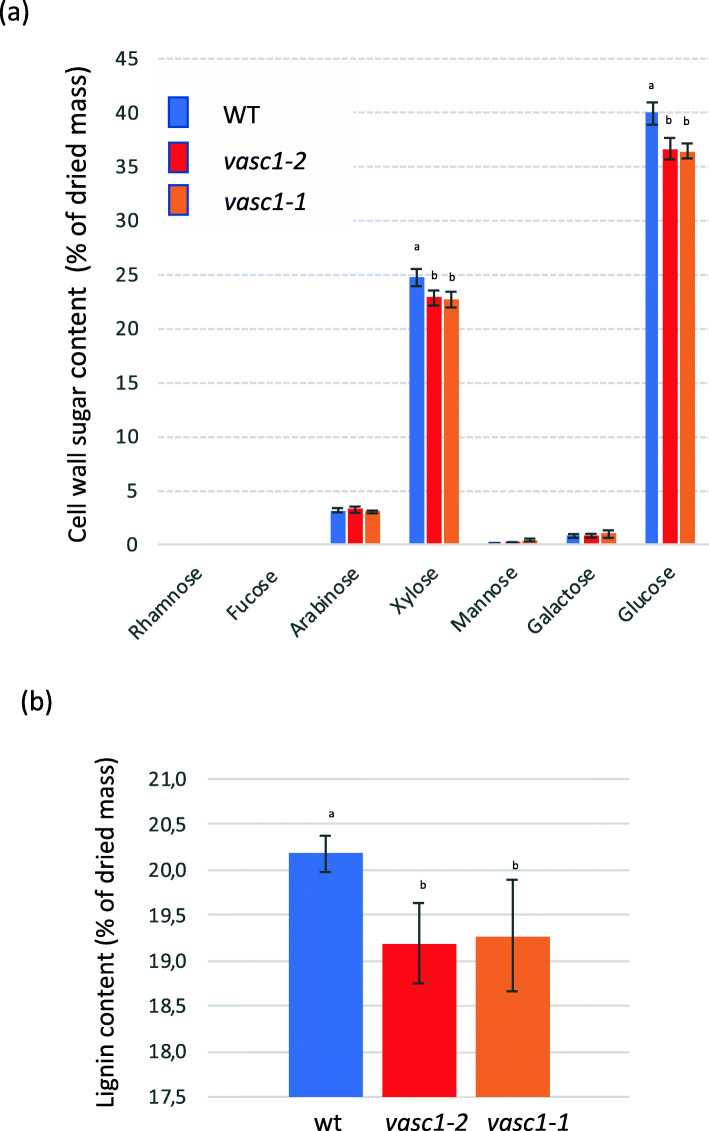
Lignocellulosic composition in *BdERECTA* deficient plants. **a** Soluble-free cell wall sugar content in mutants and WT stems. **b** Acetyl Bromide lignin content in mature dried mutants (*vasc1–1* and *vasc1–2*) and WT stems. Error bars are standard deviations. Letters above histograms indicate significant differences (*p* < 0.05) between genotypes after Tuckey’s pairwise test (*n* = 3)

Vascular tissues are enriched in secondary cell walls. The severe phenotype observed in the mutant vasculature prompted us to quantify whether cell wall sugar and lignin content from dried stems are impacted or not. Hydrolysis of soluble-free dried stem material followed by gas chromatography analysis highlighted a significant decrease in xylose and glucose in the mutants (Fig. 8a). These results suggest less xylan and cellulose content (the main polysaccharides in Brachypodium stem) in *vasc1–1* and *vasc1–2* stems. Quantification of lignin content with acetyl bromide showed that lignin deposition was reduced by 5% in both *vasc1–1* and *vasc1–2* mutants compared to WT (Fig. 8b).

## Discussion

Brachypodium displays a typical C3 monocot vascular bundle organization in its stem similar to rice, wheat or barley [19, 26, 27, 44]. The number of vascular bundles in each internode depends on the internode position along the stem. This phenomenon might be correlated with the size of the leaves that are connected to nodes because first leaves are thinner than leaves emerging later. Interestingly, we noticed that the organization of VBs is more variable in the first internode with some VBs displaying an amphivasal organization. In upper phytomeres, amphivasal organization and anastomosis of VBs were never observed in internodes of WT plants but only in *vasc1–1* and *vasc1–2* mutants. Nevertheless, anastomosis naturally occurs in the node of WT plants. This organization is required to connect the leaf veins to the nodes. We presume that the three main vascular bundles that do not anastomose in the node preserve the water supply to the stem above the node. Indeed, any damage in a leaf connected to a node could induce embolisms that would completely interrupt water traffic in the node and consequently in the upper part of the main stem. Overall, this vascular network resembles the organization described in rice by [45]. Our data show that BdERECTA controls the correct pattern of the vascular bundle in the internodes. Recently, KNOTTED1-like homeobox (KNOX) transcription factors, BLH12 and BLH14 were proposed to prevent precocious anastomosis of provascular bundles in young stems of corn [6]. The *blh12/14* double mutant shows a reduction in plant height like *vasc1–1* and *vasc1–2* but also fewer veins, a phenotype not observed in *vasc1–1* and *vasc1–2*. In dicots, the class I KNOTTED1-like homeobox (KNOX) transcription factor BREVIPEDICELLUS is a component that controls the activity of the vascular cambium in Arabidopsis and interestingly, data indicated that regulation of the xylem development by *ERECTA*, *ERECTA-like1* and SOBIR1/EVR is BREVIPEDICELLUS-dependent [31, 46, 47]. Arabidopsis *ERECTA*, *ERECTA-like1* and SOBIR1/EVR also prevent premature initiation of the fiber differentiation process and consequently lignification (a key phenomenon during the fiber differentiation) could be enhanced in the *BdERECTA*-loss-of-function *vasc1* mutants. This was not the case. On the contrary, dried mature *vasc1* mutants had less polysaccharide and lignin contents. While it is impossible to determine that BdERECTA is directly controlling cell wall content and composition in this study, perturbation of the vasculature might partly explain these changes. We did not notice a severe phenotype in the shape of interfascicular fibers and *vasc1* stems did not tend to bend more than WT, a phenomenon expected in case of cell wall deficiency. However, we can not exclude that the reduced size of both mutants (which have impaired internode elongation) compared to WT may be caused by a cell wall defect. Interestingly, lower lignin content in *vasc1* mutants is in line with the recent observation that overexpression of the sorghum *ERECTA* gene, *SbER2–1* in maize induces higher lignin content in response to moderate and severe drought stress [48].

Our microarray analysis suggests that phloem function is impacted in *vasc1–1* at 20 DAG because transcripts of Arabidopsis sweet 7 and 11 homologs as well as of a PP2 protein were overexpressed. The latter belongs to a gene family that plays important roles in the function and the integrity of sieve elements and companion cells [49, 50]. The presence of misshaped sieve elements in mutants shows unambiguously that phloem element differentiation is under the control of BdERECTA. Xylem development also suffers from the absence of a functional *BdERECTA* allele. Xylem tissue is disorganized inside VBs and the role of BdERECTA in xylem differentiation and proliferation is confirmed by the downregulation of an ortholog of tracheary element differentiation-related 7 (*TED7*, *At5g48920*). Indeed, in Arabidopsis, inducible TED7 RNAi lines displayed aberrant vessel elements (discontinuous or gapped vessels in the metaxylem) with unusual secondary cell wall [43]. The recurrence of the amphivasal phenotype in both mutants suggests proliferation of xylem at the expense the phloem tissue, however in “normal” collateral VBs, we found larger phloem area in some VBs of the *vasc1–2* mutant (this difference is not significant in *vasc1–1*). While we were not able to characterize tissue-specific expression of *BdERECTA* inside the VB, our LCM data show that *BdERECTA* expression in VB and parenchyma of shoot apex is similar. Considering that Arabidopsis *ER* expression is detected in phloem and xylem tissues of Arabidopsis stem [51] and that both xylem and phloem tissues are impacted in *vasc1–1* and *vasc1–2,* it is highly probable that similar mechanism of regulation occurs both in Brachypodium and Arabidopsis. However, our data show that *BdEPFL1*, a putative ligand of BdERECTA, is more abundant in the parenchyma tissue of the shoot apex than in developing VB. As observed previously for ERL1 in endodermis of Arabidopsis, this result questions the role of putative ligand located in parenchyma in the vascular bundle ontogeny both in patterning and organization. Interestingly, [39] suggested *RAE2* gene in rice, an ortholog of EPFL1 may function to promote the proliferation of vasculature cells necessary for awn elongation as also reported by [51] for AtEPFL4 and AtEPFL6. Consequently, the high expression level of *BdEPFL1* in the Brachypodium shoot apex parenchyma would illustrate its putative role in VB patterning. At last, the fact that *vasc1–1* and *vasc1–2* display more VBs suggests that, in contrary BdERECTA is a negative regulator of VB number. Another hypothesis, is that in the absence of BdERECTA, BdERECTA-like1 promotes VB abundance.

Our results show that hormone levels are dramatically modified in *vasc1–1*. In monocotyledonous plants, cell division and elongation occur along the base of the internode while mature tissues are located at the top so it is not surprising to detect an auxin gradient in the internode. The role of auxin transport element has been recently studied in Brachypodium [37, 52]. Interestingly *Bdaux1* displays a shorter stem and sterility such as *vasc1–1*. Auxin signaling was shown to be modified in the Arabidopsis *erecta* mutant [22] and our results show that in Brachypodium, BdERECTA impacts auxin content in the stem. This result is in line with previous data showing that *er erl1 erl2* triple mutant contains a low level of auxin [22] and that increasing exogenous or endogenous auxin levels could partially rescue the cell elongation defects of the *er erl1 erl2* triple mutant. It is, however, interesting to note that auxin content is impacted in the *vasc1* peduncles while there their size are not changed in contrast to lower internodes. Loss of the entire ERECTA family genes in Arabidopsis led to abnormal flower development and ovule differentiation [23]. These defects are believed to be the consequence of severely reduced cell proliferation. Our findings suggest that disrupting the *BdERECTA* gene only is sufficient to cause phenotypes similar to the Arabidopsis *erecta* triple mutant and thus there is less gene redundancy in Brachypodium despite the presence of *BdERECTA-like 1* whose role remains to be discovered. Less is known about the interaction of ERECTA and cytokinins in grasses [53]. demonstrate that cytokinin negatively regulate protoxylem differentiation and that AHP6 counteracts cytokinin signaling, allowing protoxylem formation in Arabidopsis. This would partly explain why specific phloem genes such as PP2 and phloem sucrose transporters are overexpressed in ERECTA and why in contrary to phloem tissue both protoxylem and metaxylem are abundant in *vasc1* mutants. Moreover, [54] suggested that cytokinin signaling specifies meristematic activity that influences the amplitude of the cambial auxin gradient in poplar. Our work suggests that hormone crosstalk orchestrates vascular tissue organization in Brachypodium stem VB although cambium is absent in this species. Indeed, our work suggests that loss of BdERECTA activity increases cytokinin content in stems. Recent work indicates that cytokinin signaling plays crucial roles in regulating rice growth and development [55] and very recently [25] reported that ERECTA1 (OsER1) is a negative regulator of spikelet number per panicle without impacting grain yield and acts upstream of the OsMKKK10-OsMKK4-OsMPK6 cascade [25]. Several lines of evidence suggest interaction between ethylene and BdERECTA plays a role in regulating vasculature development. Ethylene signaling is up-regulated in the *tdr* mutant [56] and it is known that TDR and ERECTA pathways both contribute to procambium cell maintenance [32, 57]. Our data also show upregulation of EFE, a gene we found differentially expressed in VBs. Overall, a functional ERECTA protein impacts the balance between auxin and cytokinin in grass stems (at least in Brachypodium and rice), in addition to partially controling phloem-xylem pattern in the stem.

## Conclusion

*BdERECTA* belongs to the LRR receptor-like serine/threonine-protein kinase family that has been shown to regulate multiple developmental processes. In rice, ERECTA has a major effect on panicle formation but no defect in vasculature has been reported in *Oser1* to our knowledge. Our work, clearly shows that *BdERECTA* has a pleiotropic role in Brachypodium. Mutations disrupting *BdERECTA* function result in disorganization of the vascular bundle pattern within the main stem. More striking, these mutations alter the internal organization of some vascular bundles resulting in an amphivasal organization of the vascular elements, an organization that is normally only found in the first internode. However, only some VBs (not all of them) in the mutants display an amphivasal phenotype. While we do not know how the transition between VB morphologies is controlled, this switch may be tuned by the hormonal balance, particularly the auxin/cytokinin equilibrium, which is clearly altered in *vasc1–1* compared to WT. The EPF/EPFL-like ligands of BdERECTA are not known to date, their study may partially explain why and how VB organization is controlled by ERECTA in grasses.

## Material and methods

### Plant materials

Seeds of Brachypodium (accession Bd21–3 [58]) were planted in soil and watered twice a week with water or nutrient solution (Plant Prod. NPK 15–15-30, 1 g/l) from germination to desiccation (around 70 DAG). Plants grew under a 18 h light/6 h dark cycle at 21/18 °C. Light intensity was 130 me.m^− 1^.s^− 1^. For the quantification of VBs in WT plants, nodes were numbered from the first emerging internode to the last internode (also called peduncle in this manuscript) below spikelets. For growth quantification, plantlets were measured from the base of the first node to the top of the most recent leaf or spikelet when plants have flowered.

### Hormone content analysis

For chemical analysis, young flowers, top leaves, peduncles and first internodes below the peduncle (top and base) of homozygous and heterozygous *vasc1–1* and WT were harvested from flowering plants (42 DAG, with fully elongated stems).

For each sample, 1 mg of dry powder was extracted with 0.8 mL of acetone/water/acetic acid (80/19/1 v:v:v). Abscisic acid, salicylic acid, jasmonic acid, indole-3-acetic acid and cytokinins stable labelled isotopes used as internal standards were prepared as described in [59]. One ng of each standard was added to the sample. The extract was vigorously shaken for 1 min, sonicated for 1 min at 25 Hz, shaken for 10 min at 10 °C in a Thermomixer (Eppendorf®, and then centrifuged (8000 g, 10 °C, 10 min.). The supernatants were collected, and the pellets were re-extracted twice with 0.4 mL of the same extraction solution, then vigorously shaken (1 min) and sonicated (1 min; 25 Hz). After the centrifugations, the three supernatants were pooled and dried (final volume 1.6 mL).

Each dry extract was dissolved in 100 μL of acetonitrile/water (50/50 v/v), filtered, and analyzed using a Waters Acquity Ultra Performance Liquid Chromatography coupled to a Waters Xevo Triple quadrupole mass spectrometer TQS (UPLC-ESI-MS/MS). The compounds were separated on a reverse-phase column (Uptisphere C18 UP3HDO, 100*2.1 mm*3 μm particle size; Interchim, France) using a flow rate of 0.4 mL min^− 1^ and a binary gradient: (A) acetic acid 0.1% in water (v/v) and (B) acetonitrile with 0.1% acetic acid, the column temperature was 40 °C. For Abscisic acid, salicylic acid, jasmonic acid, and indole-3-acetic acid, we used the following binary gradient (time, % A): (0 min., 98%), (3 min., 70%), (7.5 min., 50%), (8.5 min., 5%), (9.6 min., 0%), (13.2 min., 98%), (15.7 min., 98%), and for cytokinins (time, % A): (0 min., 95%), (13 min., 40%), (16 min., 0%), (16.5 min., 95%), Mass spectrometry was conducted in electrospray and Multiple Reaction Monitoring scanning mode (MRM mode), in positive ion mode for the indole-3-acetic acid and in negative ion mode for the other hormones. Relevant instrumental parameters were set as follows: capillary 1.5 kV (negative mode), source block and desolvation gas temperatures 130 °C and 500 °C, respectively. Nitrogen was used to assist the cone and desolvation (150 L h^− 1^ and 800 L h^− 1^, respectively), argon was used as the collision gas at a flow of 0.18 mL min^− 1^ as described in [59].

### Sample preparation for studying vascular organization along the stem

For vascular bundle network studies among the whole plant, of the main stem on a 45–50 DAG plants were harvested and fixed in 4% paraformaldehyde (Sigma-Aldrich) in 1 x Phosphate Buffered Saline (PBS, Eurobio), buffer-0,1% tritonX100 (Bioprobe) under vacuum on ice for 2 h. Samples were incubated at 4 °C O/N in the fixative and then washed in PBS and store at 4 °C until use. Thirty μm section were obtain with a HM 650 V Vibratome from MicroMicrotech France. For 3D reconstruction of the node, the 3 cm of the fifth node including upper and lower internode of the main WT stem were fixed as described above and later incubated in 10%, then 20% sucrose for 1 h each, and 30% sucrose overnight at 4 °C. After removing the excess of sucrose, samples were embedded in cryo-embedding medium (NEG-50TM, Thermo scientific) and freeze with liquid nitrogen. Tissue cutting were performed at − 16 °C (CryostarTM NX70, Thermo scientific) and 100 μm section were placed on superfrost slides (Thermo scientific) and stored at − 20 °C or mounted in citifluor AF1 (agar scientific) for confocal imaging.

### Confocal imaging and scanning electron microscopy

Images were captured on a ZEISS 710 confocal microscope equipped with a 405 nm diode, using a Plan APOCHROMAT X25 oil immersion objective (NA 0.8, WD 0.57 μm). The 3D-stack images with a voxel resolution of 0.24 μm in the XY plane and of 4 or 8 μm in Z-axis were acquired. Cell wall autofluorescence was revealed using the UV light (405 diode). For scanning electron microscopy, 30 μm-cross sections were made in the top part of the internode below the floral peduncle. These plant cross-sections were analyzed using an environmental scanning electron microscope (ESEM, Quattro S, Thermo Scientific, US). Images were obtained under environmental conditions without any sputter-coating of the specimens (10 °C and 1000 Pa, resulting in a humidity around 84%RH). An accelerating voltage of 10 kV was used.

### 3D reconstruction of vascular bundle network

Confocal images of serial sections were loaded into FreeD software and vascular bundles were manually segmented at least every 100 μm on 3.6 mm length in total. Sdfexport and sviewer, tools related to FreeD software were used to generate and visualize 3D reconstruction of vascular bundle network.

### Laser capture microdissection and RNA extraction

Plants were grown under a 18 h light/6 h dark cycle at 18/21 °C. Top nodes (1 cm) of the main stem on 20 DAG plants (prior flowering) were harvested and were embedded in cryo-embedding medium (NEG-50TM,Thermo scientific), then frozen with liquid nitrogen.

Samples were sectioned at 30 μm thickness using a cryo-microtome (CryostarTM NX70, Thermo scientific) at − 20 °C and mounted on polyethylene napthalate (PEN)-membrane slides (Zeiss) in RNase-free conditions.

Laser capture microdissection were performed, using a PALM MicroBeam system (Zeiss). From serial sections, each vascular bundle tissue was microdissected independently to minimize contamination from adjacent cell and tissue types. Three biological replicates were captured for each developmental stage and tissue type. Each biological replicate consisted of captured tissue from one to three individual plants (from 10 to 20 micro-dissected elements). All tissues were captured in a collected tube with adhesive cap (Zeiss) within 10 min to maximize RNA quality. Microdissected elements were harvested and incubated directly into RNA extraction buffer. Total RNA was extracted (PicoPure® RNA Isolation Kit; LifeTechnologies) following treatment of the samples on the RNA purification column with RNase-free DNase (1:8 dilution of DNase I in RDD buffer; Qiagen). RNA quantity and quality were checked by microcapillary electrophoresis (RNA 6000 Pico Chip, Agilent 2100 BioAnalyzer; Agilent Technologies). RNA Integrity Numbers (RIN) obtained were around 7.1.

### RNA-Seq

cDNA syntheses were performed using the SMARTer Ultra Low Input RNA Kit for Sequencing-v4 (Takara Bio, California USA) and libraries were prepared according to DNA Sample Preparation Illumina kit instructions with a different bar code for each sample (Illumina, California, U.S.A.). Samples were sequenced using an Illumina to produce 100 bp paired-end stranded reads from a 260 bp size selected library. Approximately 28 million of paired-end stranded reads per sample were produced.

### RNA-seq bioinformatic treatments and analyses

RNA-Seq preprocessing included trimming library adapters and performing quality controls. The raw data (fastq) were trimmed with Trimmomatic [60] tool for Phred Quality Score Qscore > 20, read length > 30 bases, and ribosome sequences were removed with tool sortMeRNA [61].

The mapper Bowtie version 2 [62] was used to align reads against the Brachypodium transcriptome (with local option and other default parameters). The abundance of each 34,310 gene was calculated by a local script which parses SAM files using only paired-end reads for which both reads map unambiguously to one gene. According to these rules, around 73% of paired-end stranded reads were counted. Differential expression analyses followed the procedure described in [63]. Briefly, genes with less than 1 read after a count per million (CPM) normalization in at least half of the samples were discarded. Library size was normalized using the trimmed mean of M-value (TMM) method and count distribution was modeled with a negative binomial generalized linear model. Dispersion was estimated by the edgeR method (Version 1.12.0, [64]) in the statistical software ‘R’ (Version 3.2.5 R Development Core Team (2005)). Expression differences compared 3 samples using likelihood ratio test and *p*-values were adjusted by the Benjamini-Hochberg procedure to control False Discovery Rate (FDR). A gene was declared differentially expressed if its adjusted *p*-value < 0.05.

### Microarray analysis

The progeny from 4 heterozygous descending lines of the original heterozygous v*asc1–1* line were sown in growth chambers and grown under identical conditions (6 to 10 individuals per lines). All plants were genotyped to classify individuals into two groups (heterozygous or homozygous, for the locus of interest). Four independent biological replicates were produced. For microarray experiment, the whole plant (excluding roots) of genotyped homozygous and heterozygous plants were harvested at 20 DAG (vegetative stage). Leaves were removed to keep the main stems and the leaf sheaths and thus frozen into liquid nitrogen. RNA were extracted with the RNAeasy kit from QIAGEN and purified with RNase-free DNase according to QIAGEN advices. RNA quality control and microarray analysis were carried out at the Institute of Plant Sciences Paris-Saclay (IPS2, Gif sur Yvette, France), by POPS Platform of this Institute as described in [65] with the following modifications. We used the 4plex_Brachypodium array (AMADID 066852) based on AGILENT technology. The single high density 4plex_ Brachypodium microarray slide contains a total of 127,258 features, distributed in four chambers. Each chamber contains 31,651 primers and 654 controls.

### Statistical analysis of microarray data

Experiments were designed with the Genomic Networks team of IPS2. For each array, the raw data comprised the logarithm of median feature pixel intensity at wavelengths 635 nm (red) and 532 nm (green). For each array, a global intensity-dependent normalization using the loess procedure [66] was performed to correct the dye bias. The differential analysis is based on the log-ratios averaging over the duplicate probes and over the technical replicates. Hence the numbers of available data for each gene equals the number of biological replicates and are used to calculate the moderated t-test [67].

Under, the null hypothesis, no evidence that the specific variances vary between probes is highlighted by Limma and consequently the moderated t-statistic is assumed to follow a standard normal distribution. To control the false discovery rate, adjusted *p*-values found using the optimized FDR approach of [68] are calculated. We considered as being differentially expressed the probes with an adjusted p-value ≤0.05.Analysis was done with the R software. The function SqueezeVar of the library limma has been used to smooth the specific variances by computing empirical Bayes posterior means. The library kerfdr has been used to calculate the adjusted p-values.

### DNA sequencing and variant calling

DNA was randomly sheared into ~ 250 bp fragments and then used to create Illumina fragment libraries. Paired end 2 × 100 sequencing was performed on Illumina HiSeq2500 at the Joint Genome Institute. Illumina reads, ~ 30 fold coverage, were aligned to Bd21–3 v1.1 reference genome (https://phytozome.jgi.doe.gov/pz/portal.html#!info?alias=Org_BdistachyonBd21_3_er) with BWA (v0.7.17) 78, filtered with Picard tools (v2.18) FixMateInformation and MarkDuplicates (https://broadinstitute.github.io/picard), then GATK (v4.0) 79 was used for base quality score recalibration, and SNV discovery using standard hard filtering parameters from GATK best practices recommendations [69, 70].

### Phylogeny analysis

The neighbor joining phylogeny tree was made from Leucine-Rich-Receptor kinase proteins from Brachypodium, *Oryza sativa, Setaria veridis, Zea mays and Arabidopsis thaliana* sharing more than 35% identity with Arabidopsis ERECTA. Node value are bootstrap values (%). Legend displays the number of substitution per kb. The analysis was performed on the Phylogeny.fr platform [71]. Sequences were aligned with MUSCLE (v3.8.31) configured for highest accuracy (MUSCLE with default settings) and curated with Gblocks for removing ambiguous regions. The final phylogeny tree was reconstructed using the maximum likelihood method implemented in the PhyML program (v3.1/3.0 aLRT). TreeDyn (v198.3) was used for tree representation.

### RT-PCR analysis

Total RNA was extracted from WT and mutant fully elongated stems (45 DAG) with EZ-10 Spin column Plant RNA Miniprep Kit following the guidelines of Biobasic (canada). Forty μl were treated with DNAse1 (New England Biolabs, USA) during 1 h. RNA there then purified with RNeasy Clean Up kit, Qiagen (Germany) and eluted in final 15 μl. RNA concentration was estimated with Epoch spectrophometer, BioTek (USA). cDNAs were produced from 500 ng of RNA with Transcriptor First Strand cDNA Synthesis Kit (Roche, Switzerland), and anchored oligo d(T). cDNAs were diluted 1/10 before use. Semi quantitative PCR were run on 1 μl of cDNA. Transcrit cDNAs were with amplified with Q5 Hot Start High-Fidelity DNA Polymerase (New England Biolabs, USA) according to the manufacturer instructions. We used the same melting hybridization (60 °C) and elongation time (10 s) with 25 to 30 cycles according to the gene of interest. Genes of interests were amplified with the following primers: *ERECTA-1*/Bradi1g46450fw: ATGGCGACGACGGCGGCGGCGTCCG, Bradi1g46450rev: CAATCTCATCAGGGATCTGGCCGGTAAGCCC; House keeping gene (Bradi5g14640) /SamDCfw: CGGCAAGCTTGCTAATCTGCTCCAAT and SamDCrev: CAGAGCAACAATAGCCTGGCTGGC.

### Cell wall neutral sugar analysis and lignin content

The neutral sugar composition of cell wall polysaccharides was monitored after hydrolysis of alcohol insoluble residues with 1 M H_2_SO_4_ for 2 h at 100 °C. A pre-hydrolysis step was carried out with 72% H_2_SO_4_ for 30 min at 25 °C to release glucose from cellulose. Each sugar was quantified after its derivatization into alditol acetates and gas chromatography analysis according to the method of [72]. Lignin content was quantified with the AcBr method as descibed in [73].

## Supplementary Information

**Additional file 1: Table S1**. Identification of SNPs in *vasc1–1*.

**Additional file 2: Table S2**. List of amino acid sequences of Leucine-Rich-Receptor kinase similar to Arabidopsis ERECTA in different species and used for phylogeny analysis (Fig. 3).

**Additional file 3: Table S3**. Statistical analysis of LCM-RNAseq data from three different types of vascular bundles and parenchyma tissue in wild-type plants.

**Additional file 4: Table S4**. Statistical analysis of genes expression level in all vascular bundles compared to parenchyma.

**Additional file 5: Table S5**. Co-expressed genes with *BdERECTA.*

**Additional file 6; Table S6**. Microarray data: Differentially expressed genes in homozygous *vasc1–1* versus heterozygous *vasc1–1*

**Additional file 7: Figure S1**. Surface area of the eight largest vascular bundles along the fifth node in the main Brachypodium stem. **Figure S2**. Mutation loci and expression of *Bradi1g46450* in WT, *vasc1–1* and *vasc1–2* .**Figure S3**. Laser capture microdissection. **Figure S4**. Validation of LCM data. **Figure S5**. Picture of WT and mutants at 20 DAG. **Figure S6**. Full length gels

## Data Availability

All data and materials used in this article are available from the authors. Microarray data from this article were deposited in the international repository GEO, Gene Expression Omnibus ([74], https://www.ncbi.nlm.nih.gov/geo/), accession no. GSE139323. All steps of the experiment, from growth conditions to bioinformatic and statistical analyses, were detailed in CATdb database ([49], https://tools.ips2.u-psud.fr/CATdb/; Project: 4 plex_Brachy_2016_01) according to the “Minimum Information About a Microarray Experiment” standards. All steps of the RNAseq experiment, from growth conditions to bioinformatic analyses, were also managed in CATdb database ([49] https://tools.ips2.u-psud.fr/CATdb/) with Project ID ANR-JC-BRAVO. This project is submitted from CATdb into the international repository GEO (Gene Expression Omnibus, [74], https://www.ncbi.nlm.nih.gov/geo) with ProjetID = GSE162395.

## Abbreviations

BP: Base pair
CPM: Count per million
DAG: Days After Germination
ESEM: Environmental scanning electron microscope
FDR: False Discovery Rate
LCM: Laser capture microdissection
LRR-RLK: Leucine-Rich Repeat Receptor Like Kinase
RIN: RNA Integrity Numbers
TMM: Trimmed mean of M-value
VB: Vascular bundle
WT: Wild-type plants
3D: 3 Dimension
