# Supplementary material for: BdERECTA controls vasculature patterning and phloem-xylem organization in Brachypodium distachyon
Source: BMC Plant Biol. 2021 Apr 23;21:196. doi: 10.1186/s12870-021-02970-2 (PMC8067424; doi:10.1186/s12870-021-02970-2)
Supplement: Supplementary file 2 — Additional file 2: Table S2. List of amino acid sequences of Leucine-Rich-Receptor kinase similar to Arabidopsis ERECTA in different species and used for phylogeny analysis (Fig. 3). [file 12870_2021_2970_MOESM2_ESM.pdf]

Additional information 3

BdERECTA controls vasculature patterning and phloem-xylem organization in *Brachypodium distachyon*.

Kaori Sakai[1], Sylvie Citerne[1], Sebastien Antelme[1], Philippe Lebris[1], Sylviane Daniel[2], Axelle Bouder[2], Angelina d'Orlando[2], Amy Cartwright[3], Frederique Tellier[1], Stéphanie Pateyron[4,5], Etienne Delannoy[4,5], Debbie Laudencia-Chingcuanco[6], Gregory Mouille[1], Jean Christophe Palauqui[1], John Vogel[3,7], Richard Sibout[1,2].

1- Institut Jean-Pierre Bourgin, INRAE, AgroParisTech, Université Paris-Saclay, 78000, Versailles, France
2- INRAE, UR BIA, F-44316, Nantes, France

3- United States Department of Energy Joint Genome Institute, Berkeley, California 94598.

4- Université Paris-Saclay, CNRS, INRAE, Univ Evry, Institute of Plant Sciences Paris-Saclay (IPS2), 91405, Orsay, France.

5- Université de Paris, CNRS, INRAE, Institute of Plant Sciences Paris-Saclay (IPS2), 91405, Orsay, France

6- USDA-ARS Western Regional Research Center, 800 Buchanan St., Albany, CA, 94710, USA

7- University of California, Berkeley, CA

Author for correspondence : Richard Sibout, richard.sibout@inrae.fr; Tel: +33 2 40 67 50 67

Table S2. Protein sequences from *Brachypodium distachyon*, *Oryza sativa, Setaria veridis, Zea Mays and Arabidopsis thaliana* sharing more than 35% identity with At2g26330 (*Arabidopsis thaliana* ERECTA). Data were downloaded from https://phytozome.jgi.doe.gov

>AT1G17230
MRGRICFLAIVILCSFSFILVRSLNEEGRVLLEFKAFLNDSNGYLASWNQLDSNPCNWTGIACTHLRTVTSV
DLNGMNLSGTLSPLICKLHGLRKLNVST
NFISGPIPQDLSLCRSLEVLDLCTNRFHGVIPIQLTMIITLKKLYLCENYLFGSIPRQIGNLSSLQELVIYSNNLT
GVIPPSMAKLRQLRIIRAGRNGFS
GVIPSEISGCESLKVLGLAENLLEGSLPKQLEKLQNLTDLILWQNRLSGEIPPSVGNISRLEVLALHENYFTGS
IPREIGKLTKMKRLYLYTNQLTGEIP
REIGNLIDAAEIDFSENQLTGFIPKEFGHILNLKLLHLFENILLGPIPRELGELTLLEKLDLSINRLNGTIPQELQF
LPYLVDLQLFDNQLEGKIPPLIG
FYSNFSVLDMSANSLSGPIPAHFCRFQTLILLSLGSNKLSGNIPRDLKTCKSLTKLMLGDNQLTGSLPIELFNL
QNLTALELHQNWLSGNISADLGKLKN
LERLRLANNNFTGEIPPEIGNLTKIVGFNISSNQLTGHIPKELGSCVTIQRLDLSGNKFSGYIAQELGQLVYLE
ILRLSDNRLTGEIPHSFGDLTRLMEL
QLGGNLLSENIPVELGKLTSLQISLNISHNNLSGTIPDSLGNLQMLEILYLNDNKLSGEIPASIGNLMSLLICNI
SNNNLVGTVPDTAVFQRMDSSNFAG
NHGLCNSQRSHCQPLVPHSDSKLNWLINGSQRQKILTITCIVIGSVFLITFLGLCWTIKRREPAFVALEDQT
KPDVMDSYYFPKKGFTYQGLVDATRNFS
EDVVLGRGACGTVYKAEMSGGEVIAVKKLNSRGEGASSDNSFRAEISTLGKIRHRNIVKLYGFCYHQNSNL
LLYEYMSKGSLGEQLQRGEKNCLLDWNAR
YRIALGAAEGLCYLHHDCRPQIVHRDIKSNNILLDERFQAHVGDFGLAKLIDLSYSKSMSAVAGSYGYIAPE
YAYTMKVTEKCDIYSFGVVLLELITGKP
PVQPLEQGGDLVNWVRRSIRNMIPTIEMFDARLDTNDKRTVHEMSLVLKIALFCTSNSPASRPTMREVV
AMITEARGSSSLSSSSITSETPLEEANSSKE
I*
>AT1G17750
MRNLGLLEITLLCSLFVYFRIDSVSSLNSDGLALLSLLKHFDKVPLEVASTWKENTSETTPCNNNWFGVICD
LSGNVVETLNLSASGLSGQLGSEIGELK
SLVTLDLSLNSFSGLLPSTLGNCTSLEYLDLSNNDFSGEVPDIFGSLQNLTFLYLDRNNLSGLIPASVGGLIELV
DLRMSYNNLSGTIPELLGNCSKLEY
LALNNNKLNGSLPASLYLLENLGELFVSNNSLGGRLHFGSSNCKKLVSLDLSFNDFQGGVPPEIGNCSSLHS
LVMVKCNLTGTIPSSMGMLRKVSVIDLS
DNRLSGNIPQELGNCSSLETLKLNDNQLQGEIPPALSKLKKLQSLELFFNKLSGEIPIGIWKIQSLTQMLVYN
NTLTGELPVEVTQLKHLKKLTLFNNGF
YGDIPMSLGLNRSLEEVDLLGNRFTGEIPPHLCHGQKLRLFILGSNQLHGKIPASIRQCKTLERVRLEDNKLS
GVLPEFPESLSLSYVNLGSNSFEGSIP
RSLGSCKNLLTIDLSQNKLTGLIPPELGNLQSLGLLNLSHNYLEGPLPSQLSGCARLLYFDVGSNSLNGSIPSS
FRSWKSLSTLVLSDNNFLGAIPQFLA
ELDRLSDLRIARNAFGGKIPSSVGLLKSLRYGLDLSANVFTGEIPTTLGALINLERLNISNNKLTGPLSVLQSLK
SLNQVDVSYNQFTGPIPVNLLSNSS
KFSGNPDLCIQASYSVSAIIRKEFKSCKGQVKLSTWKIALIAAGSSLSVLALLFALFLVLCRCKRGTKTEDANIL
AEEGLSLLLNKVLAATDNLDDKYII

GRGAHGVVYRASLGSGEEYAVKKLIFAEHIRANQNMKREIETIGLVRHRNLIRLERFWMRKEDGLMLYQY
MPNGSLHDVLHRGNQGEAVLDWSARFNIAL
GISHGLAYLHHDCHPPIIHRDIKPENILMDSDMEPHIGDFGLARILDDSTVSTATVTGTTGYIAPENAYKTV
RSKESDVYSYGVVLLELVTGKRALDRSF
PEDINIVSWVRSVLSSYEDEDDTAGPIVDPKLVDELLDTKLREQAIQVTDLALRCTDKRPENRPSMRDVVK
DLTDLESFVRSTSGSVH*
>AT1G73080
MKNLGGLFKILLLFFCLFLSTHIISVSCLNSDGLTLLSLLKHLDRVPPQVTSTWKINASEATPCNWFGITCDD
SKNVASLNFTRSRVSGQLGPEIGELKS
LQILDLSTNNFSGTIPSTLGNCTKLATLDLSENGFSDKIPDTLDSLKRLEVLYLYINFLTGELPESLFRIPKLQVL
YLDYNNLTGPIPQSIGDAKELVEL
SMYANQFSGNIPESIGNSSSLQILYLHRNKLVGSLPESLNLLGNLTTLFVGNNSLQGPVRFGSPNCKNLLTL
DLSYNEFEGGVPPALGNCSSLDALVIVS
GNLSGTIPSSLGMLKNLTILNLSENRLSGSIPAELGNCSSLNLLKLNDNQLVGGIPSALGKLRKLESLELFENR
FSGEIPIEIWKSQSLTQLLVYQNNLT
GELPVEMTEMKKLKIATLFNNSFYGAIPPGLGVNSSLEEVDFIGNKLTGEIPPNLCHGRKLRILNLGSNLLH
GTIPASIGHCKTIRRFILRENNLSGLLP
EFSQDHSLSFLDFNSNNFEGPIPGSLGSCKNLSSINLSRNRFTGQIPPQLGNLQNLGYMNLSRNLLEGSLPA
QLSNCVSLERFDVGFNSLNGSVPSNFSN
WKGLTTLVLSENRFSGGIPQFLPELKKLSTLQIARNAFGGEIPSSIGLIEDLIYDLDLSGNGLTGEIPAKLGDLI
KLTRLNISNNNLTGSLSVLKGLTSL
LHVDVSNNQFTGPIPDNLEGQLLSEPSSFSGNPNLCIPHSFSASNNSRSALKYCKDQSKSRKSGLSTWQIVL
IAVLSSLLVLVVVLALVFICLRRRKGRP
EKDAYVFTQEEGPSLLLNKVLAATDNLNEKYTIGRGAHGIVYRASLGSGKVYAVKRLVFASHIRANQSMM
REIDTIGKVRHRNLIKLEGFWLRKDDGLML
YRYMPKGSLYDVLHGVSPKENVLDWSARYNVALGVAHGLAYLHYDCHPPIVHRDIKPENILMDSDLEPHI
GDFGLARLLDDSTVSTATVTGTTGYIAPEN
AFKTVRGRESDVYSYGVVLLELVTRKRAVDKSFPESTDIVSWVRSALSSSNNNVEDMVTTIVDPILVDELLD
SSLREQVMQVTELALSCTQQDPAMRPTM
RDAVKLLEDVKHLARSCSSDSVR*
>AT1G75820
MAMRLLKTHLLFLHLYLFFSPCFAYTDMEVLLNLKSSMIGPKGHGLHDWIHSSSPDAHCSFSGVSCDDDA
RVISLNVSFTPLFGTISPEIGMLTHLVNLT
LAANNFTGELPLEMKSLTSLKVLNISNNGNLTGTFPGEILKAMVDLEVLDTYNNNFNGKLPPEMSELKKLK
YLSFGGNFFSGEIPESYGDIQSLEYLGLN
GAGLSGKSPAFLSRLKNLREMYIGYYNSYTGGVPPEFGGLTKLEILDMASCTLTGEIPTSLSNLKHLHTLFLHI
NNLTGHIPPELSGLVSLKSLDLSINQ
LTGEIPQSFINLGNITLINLFRNNLYGQIPEAIGELPKLEVFEVWENNFTLQLPANLGRNGNLIKLDVSDNHL
TGLIPKDLCRGEKLEMLILSNNFFFGP
IPEELGKCKSLTKIRIVKNLLNGTVPAGLFNLPLVTIIELTDNFFSGELPVTMSGDVLDQIYLSNNWFSGEIPP
AIGNFPNLQTLFLDRNRFRGNIPREI
FELKHLSRINTSANNITGGIPDSISRCSTLISVDLSRNRINGEIPKGINNVKNLGTLNISGNQLTGSIPTGIGN
MTSLTTLDLSFNDLSGRVPLGGQFLV
FNETSFAGNTYLCLPHRVSCPTRPGQTSDHNHTALFSPSRIVITVIAAITGLILISVAIRQMNKKKNQKSLAW
KLTAFQKLDFKSEDVLECLKEENIIGK
GGAGIVYRGSMPNNVDVAIKRLVGRGTGRSDHGFTAEIQTLGRIRHRHIVRLLGYVANKDTNLLLYEYMP
NGSLGELLHGSKGGHLQWETRHRVAVEAAK

GLCYLHHDCSPLILHRDVKSNNILLDSDFEAHVADFGLAKFLVDGAASECMSSIAGSYGYIAPEYAYTLKVD
EKSDVYSFGVVLLELIAGKKPVGEFGEG
VDIVRWVRNTEEEITQPSDAAIVVAIVDPRLTGYPLTSVIHVFKIAMMCVEEEAAARPTMREVVHMLTNP
PKSVANLIAF*
>AT2G01950
MTTSPIRVRIRTRIQISFIFLLTHLSQSSSSDQSSLKTDSLSLLSFKTMIQDDPNNILSNWSPRKSPCQFSGVT
CLGGRVTEINLSGSGLSGIVSFNAFT
SLDSLSVLKLSENFFVLNSTSLLLLPLTLTHLELSSSGLIGTLPENFFSKYSNLISITLSYNNFTGKLPNDLFLSSKK
LQTLDLSYNNITGPISGLTIPL
SSCVSMTYLDFSGNSISGYISDSLINCTNLKSLNLSYNNFDGQIPKSFGELKLLQSLDLSHNRLTGWIPPEIGD
TCRSLQNLRLSYNNFTGVIPESLSSC
SWLQSLDLSNNNISGPFPNTILRSFGSLQILLLSNNLISGDFPTSISACKSLRIADFSSNRFSGVIPPDLCPGAA
SLEELRLPDNLVTGEIPPAISQCSE
LRTIDLSLNYLNGTIPPEIGNLQKLEQFIAWYNNIAGEIPPEIGKLQNLKDLILNNNQLTGEIPPEFFNCSNIE
WVSFTSNRLTGEVPKDFGILSRLAVL
QLGNNNFTGEIPPELGKCTTLVWLDLNTNHLTGEIPPRLGRQPGSKALSGLLSGNTMAFVRNVGNSCKG
VGGLVEFSGIRPERLLQIPSLKSCDFTRMYS
GPILSLFTRYQTIEYLDLSYNQLRGKIPDEIGEMIALQVLELSHNQLSGEIPFTIGQLKNLGVFDASDNRLQG
QIPESFSNLSFLVQIDLSNNELTGPIP
QRGQLSTLPATQYANNPGLCGVPLPECKNGNNQLPAGTEEGKRAKHGTRAASWANSIVLGVLISAASVC
ILIVWAIAVRARRRDADDAKMLHSLQAVNSA
TTWKIEKEKEPLSINVATFQRQLRKLKFSQLIEATNGFSAASMIGHGGFGEVFKATLKDGSSVAIKKLIRLSC
QGDREFMAEMETLGKIKHRNLVPLLGY
CKIGEERLLVYEFMQYGSLEEVLHGPRTGEKRRILGWEERKKIAKGAAKGLCFLHHNCIPHIIHRDMKSSN
VLLDQDMEARVSDFGMARLISALDTHLSV
STLAGTPGYVPPEYYQSFRCTAKGDVYSIGVVMLEILSGKRPTDKEEFGDTNLVGWSKMKAREGKHMEVI
DEDLLKEGSSESLNEKEGFEGGVIVKEMLR
YLEIALRCVDDFPSKRPNMLQVVASLRELRGSENNSHSHSNSL*
>AT2G26330
MALFRDIVLLGFLFCLSLVATVTSEEGATLLEIKKSFKDVNNVLYDWTTSPSSDYCVWRGVSCENVTFNVV
ALNLSDLNLDGEISPAIGDLKSLLSIDLR
GNRLSGQIPDEIGDCSSLQNLDLSFNELSGDIPFSISKLKQLEQLILKNNQLIGPIPSTLSQIPNLKILDLAQNK
LSGEIPRLIYWNEVLQYLGLRGNNL
VGNISPDLCQLTGLWYFDVRNNSLTGSIPETIGNCTAFQVLDLSYNQLTGEIPFDIGFLQVATLSLQGNQLS
GKIPSVIGLMQALAVLDLSGNLLSGSIP
PILGNLTFTEKLYLHSNKLTGSIPPELGNMSKLHYLELNDNHLTGHIPPELGKLTDLFDLNVANNDLEGPIPD
HLSSCTNLNSLNVHGNKFSGTIPRAFQ
KLESMTYLNLSSNNIKGPIPVELSRIGNLDTLDLSNNKINGIIPSSLGDLEHLLKMNLSRNHITGVVPGDFGN
LRSIMEIDLSNNDISGPIPEELNQLQN
IILLRLENNNLTGNVGSLANCLSLTVLNVSHNNLVGDIPKNNNFSRFSPDSFIGNPGLCGSWLNSPCHDSR
RTVRVSISRAAILGIAIGGLVILLMVLIA
ACRPHNPPPFLDGSLDKPVTYSTPKLVILHMNMALHVYEDIMRMTENLSEKYIIGHGASSTVYKCVLKNC
KPVAIKRLYSHNPQSMKQFETELEMLSSIK
HRNLVSLQAYSLSHLGSLLFYDYLENGSLWDLLHGPTKKKTLDWDTRLKIAYGAAQGLAYLHHDCSPRIIH
RDVKSSNILLDKDLEARLTDFGIAKSLCV
SKSHTSTYVMGTIGYIDPEYARTSRLTEKSDVYSYGIVLLELLTRRKAVDDESNLHHLIMSKTGNNEVMEM
ADPDITSTCKDLGVVKKVFQLALLCTKRQ

PNDRPTMHQVTRVLGSFMLSEQPPAATDTSATLAGSCYVDEYANLKTPHSVNCSSMSASDAQLFLRFG
QVISQNSE*
>AT2G33170
MGWWIFEFKKESKSMFVGVLFLLTLLVWTSESLNSDGQFLLELKNRGFQDSLNRLHNWNGIDETPCNWI
GVNCSSQGSSSSSNSLVVTSLDLSSMNLSGI
VSPSIGGLVNLVYLNLAYNALTGDIPREIGNCSKLEVMFLNNNQFGGSIPVEINKLSQLRSFNICNNKLSGPL
PEEIGDLYNLEELVAYTNNLTGPLPRS
LGNLNKLTTFRAGQNDFSGNIPTEIGKCLNLKLLGLAQNFISGELPKEIGMLVKLQEVILWQNKFSGFIPKDI
GNLTSLETLALYGNSLVGPIPSEIGNM
KSLKKLYLYQNQLNGTIPKELGKLSKVMEIDFSENLLSGEIPVELSKISELRLLYLFQNKLTGIIPNELSKLRNLA
KLDLSINSLTGPIPPGFQNLTSMR
QLQLFHNSLSGVIPQGLGLYSPLWVVDFSENQLSGKIPPFICQQSNLILLNLGSNRIFGNIPPGVLRCKSLLQ
LRVVGNRLTGQFPTELCKLVNLSAIEL
DQNRFSGPLPPEIGTCQKLQRLHLAANQFSSNLPNEISKLSNLVTFNVSSNSLTGPIPSEIANCKMLQRLDL
SRNSFIGSLPPELGSLHQLEILRLSENR
FSGNIPFTIGNLTHLTELQMGGNLFSGSIPPQLGLLSSLQIAMNLSYNDFSGEIPPEIGNLHLLMYLSLNNN
HLSGEIPTTFENLSSLLGCNFSYNNLTG
QLPHTQIFQNMTLTSFLGNKGLCGGHLRSCDPSHSSWPHISSLKAGSARRGRIIIIVSSVIGGISLLLIAIVVH
FLRNPVEPTAPYVHDKEPFFQESDIY
FVPKERFTVKDILEATKGFHDSYIVGRGACGTVYKAVMPSGKTIAVKKLESNREGNNNNSNNTDNSFRAE
ILTLGKIRHRNIVRLYSFCYHQGSNSNLLL
YEYMSRGSLGELLHGGKSHSMDWPTRFAIALGAAEGLAYLHHDCKPRIIHRDIKSNNILIDENFEAHVGDF
GLAKVIDMPLSKSVSAVAGSYGYIAPEYA
YTMKVTEKCDIYSFGVVLLELLTGKAPVQPLEQGGDLATWTRNHIRDHSLTSEILDPYLTKVEDDVILNHMI
TVTKIAVLCTKSSPSDRPTMREVVLMLI
ESGERAGKVIVSTTCSDLPPPAPP*
>AT3G24240
MSLHSLIFFSSSSSSLLFSFFFIFIFCFSLSDAEQNPEASILYSWLHSSSPTPSSLSLFNWNSIDNTPCNNWTFI
TCSSQGFITDIDIESVPLQLSLPKN
LPAFRSLQKLTISGANLTGTLPESLGDCLGLKVLDLSSNGLVGDIPWSLSKLRNLETLILNSNQLTGKIPPDIS
KCSKLKSLILFDNLLTGSIPTELGKL
SGLEVIRIGGNKEISGQIPSEIGDCSNLTVLGLAETSVSGNLPSSLGKLKKLETLSIYTTMISGEIPSDLGNCSEL
VDLFLYENSLSGSIPREIGQLTKL
EQLFLWQNSLVGGIPEEIGNCSNLKMIDLSLNLLSGSIPSSIGRLSFLEEFMISDNKFSGSIPTTISNCSSLVQL
QLDKNQISGLIPSELGTLTKLTLFF
AWSNQLEGSIPPGLADCTDLQALDLSRNSLTGTIPSGLFMLRNLTKLLLISNSLSGFIPQEIGNCSSLVRLRL
GFNRITGEIPSGIGSLKKINFLDFSSN
RLHGKVPDEIGSCSELQMIDLSNNSLEGSLPNPVSSLSGLQVLDVSANQFSGKIPASLGRLVSLNKLILSKNL
FSGSIPTSLGMCSGLQLLDLGSNELSG
EIPSELGDIENLEIALNLSSNRLTGKIPSKIASLNKLSILDLSHNMLEGDLAPLANIENLVSLNISYNSFSGYLPD
NKLFRQLSPQDLEGNKKLCSSTQD
SCFLTYRKGNGLGDDGDASRTRKLRLTLALLITLTVVLMILGAVAVIRARRNIDNERDSELGETYKWQFTPF
QKLNFSVDQIIRCLVEPNVIGKGCSGVV
YRADVDNGEVIAVKKLWPAMVNGGHDEKTKNVRDSFSAEVKTLGTIRHKNIVRFLGCCWNRNTRLLMY
DYMPNGSLGSLLHERRGSSLDWDLRYRILLGA
AQGLAYLHHDCLPPIVHRDIKANNILIGLDFEPYIADFGLAKLVDEGDIGRCSNTVAGSYGYIAPEYGYSMKI
TEKSDVYSYGVVVLEVLTGKQPIDPTV

PEGIHLVDWVRQNRGSLEVLDSTLRSRTEAEADEMMQVLGTALLCVNSSPDERPTMKDVAAMLKEIKQ
EREEYAKVDLLLKKSPPPTTTMQEECRKNEMM
MIPAAAASSSKEMRREERLLKSNNTSFSASSLLYSSSSSIE*
>AT3G49670
MKLLLLLLLLLLLHISHSFTVAKPITELHALLSLKSSFTIDEHSPLLTSWNLSTTFCSWTGVTCDVSLRHVTSLD
LSGLNLSGTLSSDVAHLPLLQNLSL
AANQISGPIPPQISNLYELRHLNLSNNVFNGSFPDELSSGLVNLRVLDLYNNNLTGDLPVSLTNLTQLRHLH
LGGNYFSGKIPATYGTWPVLEYLAVSGN
ELTGKIPPEIGNLTTLRELYIGYYNAFENGLPPEIGNLSELVRFDAANCGLTGEIPPEIGKLQKLDTLFLQVNA
FTGTITQELGLISSLKSMDLSNNMFT
GEIPTSFSQLKNLTLLNLFRNKLYGAIPEFIGEMPELEVLQLWENNFTGSIPQKLGENGRLVILDLSSNKLTG
TLPPNMCSGNRLMTLITLGNFLFGSIP
DSLGKCESLTRIRMGENFLNGSIPKELFGLPKLSQVELQDNYLTGELPISGGGVSGDLGQISLSNNQLSGSL
PAAIGNLSGVQKLLLDGNKFSGSIPPEI
GRLQQLSKLDFSHNLFSGRIAPEISRCKLLTFVDLSRNELSGDIPNELTGMKILNYLNLSRNHLVGSIPVTIAS
MQSLTSVDFSYNNLSGLVPSTGQFSY
FNYTSFVGNSHLCGPYLGPCGKGTHQSHVKPLSATTKLLLVLGLLFCSMVFAIVAIIKARSLRNASEAKAWR
LTAFQRLDFTCDDVLDSLKEDNIIGKGG
AGIVYKGTMPKGDLVAVKRLATMSHGSSHDHGFNAEIQTLGRIRHRHIVRLLGFCSNHETNLLVYEYMP
NGSLGEVLHGKKGGHLHWNTRYKIALEAAKG
LCYLHHDCSPLIVHRDVKSNNILLDSNFEAHVADFGLAKFLQDSGTSECMSAIAGSYGYIAPEYAYTLKVDE
KSDVYSFGVVLLELITGKKPVGEFGDGV
DIVQWVRSMTDSNKDCVLKVIDLRLSSVPVHEVTHVFYVALLCVEEQAVERPTMREVVQILTEIPKIPLSK
QQAAESDVTEKAPAINESSPDSGSPPDLL
SN*
>AT4G20140
MQPLVLLLLFILCFSGLGQPGIINNDLQTLLEVKKSLVTNPQEDDPLRQWNSDNINYCSWTGVTCDNTGL
FRVIALNLTGLGLTGSISPWFGRFDNLIHL
DLSSNNLVGPIPTALSNLTSLESLFLFSNQLTGEIPSQLGSLVNIRSLRIGDNELVGDIPETLGNLVNLQMLAL
ASCRLTGPIPSQLGRLVRVQSLILQD
NYLEGPIPAELGNCSDLTVFTAAENMLNGTIPAELGRLENLEILNLANNSLTGEIPSQLGEMSQLQYLSLM
ANQLQGLIPKSLADLGNLQTLDLSANNLT
GEIPEEFWNMSQLLDLVLANNHLSGSLPKSICSNNTNLEQLVLSGTQLSGEIPVELSKCQSLKQLDLSNNSL
AGSIPEALFELVELTDLYLHNNTLEGTL
SPSISNLTNLQWLVLYHNNLEGKLPKEISALRKLEVLFLYENRFSGEIPQEIGNCTSLKMIDMFGNHFEGEIP
PSIGRLKELNLLHLRQNELVGGLPASL
GNCHQLNILDLADNQLSGSIPSSFGFLKGLEQLMLYNNSLQGNLPDSLISLRNLTRINLSHNRLNGTIHPLC
GSSSYLSFDVTNNGFEDEIPLELGNSQN
LDRLRLGKNQLTGKIPWTLGKIRELSLLDMSSNALTGTIPLQLVLCKKLTHIDLNNNFLSGPIPPWLGKLSQL
GELKLSSNQFVESLPTELFNCTKLLVL
SLDGNSLNGSIPQEIGNLGALNVLNLDKNQFSGSLPQAMGKLSKLYELRLSRNSLTGEIPVEIGQLQDLQS
ALDLSYNNFTGDIPSTIGTLSKLETLDLS
HNQLTGEVPGSVGDMKSLGYLNVSFNNLGGKLKKQFSRWPADSFLGNTGLCGSPLSRCNRVRSNNKQQ
GLSARSVVIISAISALTAIGLMILVIALFFKQ
RHDFFKKVGHGSTAYTSSSSSSQATHKPLFRNGASKSDIRWEDIMEATHNLSEEFMIGSGGSGKVYKAEL
ENGETVAVKKILWKDDLMSNKSFSREVKTL

GRIRHRHLVKLMGYCSSKSEGLNLLIYEYMKNGSIWDWLHEDKPVLEKKKKLLDWEARLRIAVGLAQGVE
YLHHDCVPPIVHRDIKSSNVLLDSNMEAHL
GDFGLAKVLTENCDTNTDSNTWFACSYGYIAPEYAYSLKATEKSDVYSMGIVLMEIVTGKMPTDSVFGAE
MDMVRWVETHLEVAGSARDKLIDPKLKPLL
PFEEDAACQVLEIALQCTKTSPQERPSSRQACDSLLHVYNNRTAGYKKL*
>AT4G20270
MADKIFTFFLILSSISPLLCSSLISPLNLSLIRQANVLISLKQSFDSYDPSLDSWNIPNFNSLCSWTGVSCDNLN
QSITRLDLSNLNISGTISPEISRLS
PSLVFLDISSNSFSGELPKEIYELSGLEVLNISSNVFEGELETRGFSQMTQLVTLDAYDNSFNGSLPLSLTTLT
RLEHLDLGGNYFDGEIPRSYGSFLSL
KFLSLSGNDLRGRIPNELANITTLVQLYLGYYNDYRGGIPADFGRLINLVHLDLANCSLKGSIPAELGNLKNL
EVLFLQTNELTGSVPRELGNMTSLKTL
DLSNNFLEGEIPLELSGLQKLQLFNLFFNRLHGEIPEFVSELPDLQILKLWHNNFTGKIPSKLGSNGNLIEIDL
STNKLTGLIPESLCFGRRLKILILFN
NFLFGPLPEDLGQCEPLWRFRLGQNFLTSKLPKGLIYLPNLSLLELQNNFLTGEIPEEEAGNAQFSSLTQINL
SNNRLSGPIPGSIRNLRSLQILLLGAN
RLSGQIPGEIGSLKSLLKIDMSRNNFSGKFPPEFGDCMSLTYLDLSHNQISGQIPVQISQIRILNYLNVSWNS
FNQSLPNELGYMKSLTSADFSHNNFSG
SVPTSGQFSYFNNTSFLGNPFLCGFSSNPCNGSQNQSQSQLLNQNNARSRGEISAKFKLFFGLGLLGFFLV
FVVLAVVKNRRMRKNNPNLWKLIGFQKLG
FRSEHILECVKENHVIGKGGRGIVYKGVMPNGEEVAVKKLLTITKGSSHDNGLAAEIQTLGRIRHRNIVRLL
AFCSNKDVNLLVYEYMPNGSLGEVLHGK
AGVFLKWETRLQIALEAAKGLCYLHHDCSPLIIHRDVKSNNILLGPEFEAHVADFGLAKFMMQDNGASEC
MSSIAGSYGYIAPEYAYTLRIDEKSDVYSF
GVVLLELITGRKPVDNFGEEGIDIVQWSKIQTNCNRQGVVKIIDQRLSNIPLAEAMELFFVAMLCVQEHSV
ERPTMREVVQMISQAKQPNTF*
>AT4G28490
MLYCLILLLCLSSTYLPSLSLNQDATILRQAKLGLSDPAQSLSSWSDNNDVTPCKWLGVSCDATSNVVSVD
LSSFMLVGPFPSILCHLPSLHSLSLYNNS
INGSLSADDFDTCHNLISLDLSENLLVGSIPKSLPFNLPNLKFLEISGNNLSDTIPSSFGEFRKLESLNLAGNFL
SGTIPASLGNVTTLKELKLAYNLFS
PSQIPSQLGNLTELQVLWLAGCNLVGPIPPSLSRLTSLVNLDLTFNQLTGSIPSWITQLKTVEQIELFNNSFS
GELPESMGNMTTLKRFDASMNKLTGKI
PDNLNLLNLESLNLFENMLEGPLPESITRSKTLSELKLFNNRLTGVLPSQLGANSPLQYVDLSYNRFSGEIPA
NVCGEGKLEYLILIDNSFSGEISNNLG
KCKSLTRVRLSNNKLSGQIPHGFWGLPRLSLLELSDNSFTGSIPKTIIGAKNLSNLRISKNRFSGSIPNEIGSLN
GIIEISGAENDFSGEIPESLVKLKQ
LSRLDLSKNQLSGEIPRELRGWKNLNELNLANNHLSGEIPKEVGILPVLNYLDLSSNQFSGEIPLELQNLKLN
VLNLSYNHLSGKIPPLYANKIYAHDFI
GNPGLCVDLDGLCRKITRSKNIGYVWILLTIFLLAGLVFVVGIVMFIAKCRKLRALKSSTLAASKWRSFHKLH
FSEHEIADCLDEKNVIGFGSSGKVYKV
ELRGGEVVAVKKLNKSVKGGDDEYSSDSLNRDVFAAEVETLGTIRHKSIVRLWCCCSSGDCKLLVYEYMP
NGSLADVLHGDRKGGVVLGWPERLRIALDA
AEGLSYLHHDCVPPIVHRDVKSSNILLDSDYGAKVADFGIAKVGQMSGSKTPEAMSGIAGSCGYIAPEYVY
TLRVNEKSDIYSFGVVLLELVTGKQPTDS
ELGDKDMAKWVCTALDKCGLEPVIDPKLDLKFKEEISKVIHIGLLCTSPLPLNRPSMRKVVIMLQEVSGAV
PCSSPNTSKRSKTGGKLSPYYTEDLNSV*

>AT4G28650
MKMKIIVLFLYYCYIGSTSSVLASIDNVNELSVLLSVKSTLVDPLNFLKDWKLSDTSDHCNWTGVRCNSNG
NVEKLDLAGMNLTGKISDSISQLSSLVSF
NISCNGFESLLPKSIPPLKSIDISQNSFSGSLFLFSNESLGLVHLNASGNNLSGNLTEDLGNLVSLEVLDLRGN
FFQGSLPSSFKNLQKLRFLGLSGNNL
TGELPSVLGQLPSLETAILGYNEFKGPIPPEFGNINSLKYLDLAIGKLSGEIPSELGKLKSLETLLLYENNFTGTI
PREIGSITTLKVLDFSDNALTGEI
PMEITKLKNLQLLNLMRNKLSGSIPPAISSLAQLQVLELWNNTLSGELPSDLGKNSPLQWLDVSSNSFSGEI
PSTLCNKGNLTKLILFNNTFTGQIPATL
STCQSLVRVRMQNNLLNGSIPIGFGKLEKLQRLELAGNRLSGGIPGDISDSVSLSFIDFSRNQIRSSLPSTILSI
HNLQAFLVADNFISGEVPDQFQDCP
SLSNLDLSSNTLTGTIPSSIASCEKLVSLNLRNNNLTGEIPRQITTMSALAVLDLSNNSLTGVLPESIGTSPALE
LLNVSYNKLTGPVPINGFLKTINPD
DLRGNSGLCGGVLPPCSKFQRATSSHSSLHGKRIVAGWLIGIASVLALGILTIVTRTLYKKWYSNGFCGDET
ASKGEWPWRLMAFHRLGFTASDILACIK
ESNMIGMGATGIVYKAEMSRSSTVLAVKKLWRSAADIEDGTTGDFVGEVNLLGKLRHRNIVRLLGFLYND
KNMMIVYEFMLNGNLGDAIHGKNAAGRLLV
DWVSRYNIALGVAHGLAYLHHDCHPPVIHRDIKSNNILLDANLDARIADFGLARMMARKKETVSMVAGS
YGYIAPEYGYTLKVDEKIDIYSYGVVLLELL
TGRRPLEPEFGESVDIVEWVRRKIRDNISLEEALDPNVGNCRYVQEEMLLVLQIALLCTTKLPKDRPSMRD
VISMLGEAKPRRKSNSNEENTSRSLAEKH
SSVFSTSPVNGLL*
>AT5G07180
MRRIETMKGLFFCLGMVVFMLLGSVSPMNNEGKALMAIKASFSNVANMLLDWDDVHNHDFCSWRG
VFCDNVSLNVVSLNLSNLNLGGEISSALGDLMNLQ
SIDLQGNKLGGQIPDEIGNCVSLAYVDFSTNLLFGDIPFSISKLKQLEFLNLKNNQLTGPIPATLTQIPNLKTL
DLARNQLTGEIPRLLYWNEVLQYLGL
RGNMLTGTLSPDMCQLTGLWYFDVRGNNLTGTIPESIGNCTSFEILDVSYNQITGVIPYNIGFLQVATLSL
QGNKLTGRIPEVIGLMQALAVLDLSDNEL
TGPIPPILGNLSFTGKLYLHGNKLTGQIPPELGNMSRLSYLQLNDNELVGKIPPELGKLEQLFELNLANNNL
VGLIPSNISSCAALNQFNVHGNFLSGAV
PLEFRNLGSLTYLNLSSNSFKGKIPAELGHIINLDTLDLSGNNFSGSIPLTLGDLEHLLILNLSRNHLNGTLPAE
FGNLRSIQIIDVSFNFLAGVIPTEL
GQLQNINSLILNNNKIHGKIPDQLTNCFSLANLNISFNNLSGIIPPMKNFTRFSPASFFGNPFLCGNWVGSI
CGPSLPKSQVFTRVAVICMVLGFITLIC
MIFIAVYKSKQQKPVLKGSSKQPEGSTKLVILHMDMAIHTFDDIMRVTENLDEKYIIGYGASSTVYKCTSKT
SRPIAIKRIYNQYPSNFREFETELETIG
SIRHRNIVSLHGYALSPFGNLLFYDYMENGSLWDLLHGPGKKVKLDWETRLKIAVGAAQGLAYLHHDCTP
RIIHRDIKSSNILLDGNFEARLSDFGIAKS
IPATKTYASTYVLGTIGYIDPEYARTSRLNEKSDIYSFGIVLLELLTGKKAVDNEANLHQMILSKADDNTVME
AVDAEVSVTCMDSGHIKKTFQLALLCT
KRNPLERPTMQEVSRVLLSLVPSPPPKKLPSPAKVQEGEERRESHSSDTTTPQWFVQFREDISKSSL*
>AT5G07280
MAFLTALFLFLFFSFSSSAIVDLSSETTSLISFKRSLENPSLLSSWNVSSSASHCDWVGVTCLLGRVNSLSLPS
LSLRGQIPKEISSLKNLRELCLAGNQ
FSGKIPPEIWNLKHLQTLDLSGNSLTGLLPRLLSELPQLLYLDLSDNHFSGSLPPSFFISLPALSSLDVSNNSLS
GEIPPEIGKLSNLSNLYMGLNSFSG

QIPSEIGNISLLKNFAAPSCFFNGPLPKEISKLKHLAKLDLSYNPLKCSIPKSFGELHNLSILNLVSAELIGLIPPE
LGNCKSLKSLMLSFNSLSGPLPL
ELSEIPLLTFSAERNQLSGSLPSWMGKWKVLDSLLLANNRFSGEIPHEIEDCPMLKHLSLASNLLSGSIPREL
CGSGSLEAIDLSGNLLSGTIEEVFDGC
SSLGELLLTNNQINGSIPEDLWKLPLMALDLDSNNFTGEIPKSLWKSTNLMEFTASYNRLEGYLPAEIGNA
ASLKRLVLSDNQLTGEIPREIGKLTSLSV
LNLNANMFQGKIPVELGDCTSLTTLDLGSNNLQGQIPDKITALAQLQCLVLSYNNLSGSIPSKPSAYFHQIE
MPDLSFLQHHGIFDLSYNRLSGPIPEEL
GECLVLVEISLSNNHLSGEIPASLSRLTNLTILDLSGNALTGSIPKEMGNSLKLQGLNLANNQLNGHIPESFG
LLGSLVKLNLTKNKLDGPVPASLGNLK
ELTHMDLSFNNLSGELSSELSTMEKLVGLYIEQNKFTGEIPSELGNLTQLEYLDVSENLLSGEIPTKICGLPNL
EFLNLAKNNLRGEVPSDGVCQDPSKA
LLSGNKELCGRVVGSDCKIEGTKLRSAWGIAGLMLGFTIIVFVFVFSLRRWAMTKRVKQRDDPERMEESR
LKGFVDQNLYFLSGSRSREPLSINIAMFEQ
PLLKVRLGDIVEATDHFSKKNIIGDGGFGTVYKACLPGEKTVAVKKLSEAKTQGNREFMAEMETLGKVKH
PNLVSLLGYCSFSEEKLLVYEYMVNGSLDH
WLRNQTGMLEVLDWSKRLKIAVGAARGLAFLHHGFIPHIIHRDIKASNILLDGDFEPKVADFGLARLISAC
ESHVSTVIAGTFGYIPPEYGQSARATTKG
DVYSFGVILLELVTGKEPTGPDFKESEGGNLVGWAIQKINQGKAVDVIDPLLVSVALKNSQLRLLQIAMLC
LAETPAKRPNMLDVLKALKEI*
>AT5G44700
MQQNSVLLALFFLCFSSGLGSGQPGQRDDLQTLLELKNSFITNPKEEDVLRDWNSGSPSYCNWTGVTCG
GREIIGLNLSGLGLTGSISPSIGRFNNLIHI
DLSSNRLVGPIPTTLSNLSSSLESLHLFSNLLSGDIPSQLGSLVNLKSLKLGDNELNGTIPETFGNLVNLQMLA
LASCRLTGLIPSRFGRLVQLQTLILQ
DNELEGPIPAEIGNCTSLALFAAAFNRLNGSLPAELNRLKNLQTLNLGDNSFSGEIPSQLGDLVSIQYLNLIG
NQLQGLIPKRLTELANLQTLDLSSNNL
TGVIHEEFWRMNQLEFLVLAKNRLSGSLPKTICSNNTSLKQLFLSETQLSGEIPAEISNCQSLKLLDLSNNTL
TGQIPDSLFQLVELTNLYLNNNSLEGT
LSSSISNLTNLQEFTLYHNNLEGKVPKEIGFLGKLEIMYLYENRFSGEMPVEIGNCTRLQEIDWYGNRLSGEI
PSSIGRLKDLTRLHLRENELVGNIPAS
LGNCHQMTVIDLADNQLSGSIPSSFGFLTALELFMIYNNSLQGNLPDSLINLKNLTRINFSSNKFNGSISPLC
GSSSYLSFDVTENGFEGDIPLELGKST
NLDRLRLGKNQFTGRIPRTFGKISELSLLDISRNSLSGIIPVELGLCKKLTHIDLNNNYLSGVIPTWLGKLPLLG
ELKLSSNKFVGSLPTEIFSLTNILT
LFLDGNSLNGSIPQEIGNLQALNALNLEENQLSGPLPSTIGKLSKLFELRLSRNALTGEIPVEIGQLQDLQSAL
DLSYNNFTGRIPSTISTLPKLESLDL
SHNQLVGEVPGQIGDMKSLGYLNLSYNNLEGKLKKQFSRWQADAFVGNAGLCGSPLSHCNRAGSKNQ
RSLSPKTVVIISAISSLAAIALMVLVIILFFKQ
NHDLFKKVRGGNSAFSSNSSSSQAPLFSNGGAKSDIKWDDIMEATHYLNEEFMIGSGGSGKVYKAELKN
GETIAVKKILWKDDLMSNKSFNREVKTLGTI
RHRHLVKLMGYCSSKADGLNLLIYEYMANGSVWDWLHANENTKKKEVLGWETRLKIALGLAQGVEYLH
YDCVPPIVHRDIKSSNVLLDSNIEAHLGDFGL
AKILTGNYDTNTESNTMFAGSYGYIAPEYAYSLKATEKSDVYSMGIVLMEIVTGKMPTEAMFDEETDMV
RWVETVLDTPPGSEAREKLIDSELKSLLPCE
EEAAYQVLEIALQCTKSYPQERPSSRQASEYLLNVFNNRAASYREMQTDTDK*
>AT5G48940

MSLQMPIPRKKALTVSHFSITLSLFLAFFISSTSASTNEVSALISWLHSSNSPPPSVFSGWNPSDSDPCQWP
YITCSSSDNKLVTEINVVSVQLALPFPP
NISSFTSLQKLVISNTNLTGAISSEIGDCSELIVIDLSSNSLVGEIPSSLGKLKNLQELCLNSNGLTGKIPPELGD
CVSLKNLEIFDNYLSENLPLELGK
ISTLESIRAGGNSELSGKIPEEIGNCRNLKVLGLAATKISGSLPVSLGQLSKLQSLSVYSTMLSGEIPKELGNCS
ELINLFLYDNDLSGTLPKELGKLQN
LEKMLLWQNNLHGPIPEEIGFMKSLNAIDLSMNYFSGTIPKSFGNLSNLQELMLSSNNITGSIPSILSNCTK
LVQFQIDANQISGLIPPEIGLLKELNIF
LGWQNKLEGNIPDELAGCQNLQALDLSQNYLTGSLPAGLFQLRNLTKLLLISNAISGVIPLEIGNCTSLVRLR
LVNNRITGEIPKGIGFLQNLSFLDLSE
NNLSGPVPLEISNCRQLQMLNLSNNTLQGYLPLSLSSLTKLQVLDVSSNDLTGKIPDSLGHLISLNRLILSKN
SFNGEIPSSLGHCTNLQLLDLSSNNIS
GTIPEELFDIQDLDIALNLSWNSLDGFIPERISALNRLSVLDISHNMLSGDLSALSGLENLVSLNISHNRFSGY
LPDSKVFRQLIGAEMEGNNGLCSKGF
RSCFVSNSSQLTTQRGVHSHRLRIAIGLLISVTAVLAVLGVLAVIRAKQMIRDDNDSETGENLWTWQFTPF
QKLNFTVEHVLKCLVEGNVIGKGCSGIVY
KAEMPNREVIAVKKLWPVTVPNLNEKTKSSGVRDSFSAEVKTLGSIRHKNIVRFLGCCWNKNTRLLMYDY
MSNGSLGSLLHERSGVCSLGWEVRYKIILG
AAQGLAYLHHDCVPPIVHRDIKANNILIGPDFEPYIGDFGLAKLVDDGDFARSSNTIAGSYGYIAPEYGYSM
KITEKSDVYSYGVVVLEVLTGKQPIDPT
IPDGLHIVDWVKKIRDIQVIDQGLQARPESEVEEMMQTLGVALLCINPIPEDRPTMKDVAAMLSEICQER
EESMKVDGCSGSCNNGRERGKDDSTSSVMQ
QTAKYLRSSSTSFSASSLLYSSSSSATSNVRPNLK*
>AT5G49660
MRLKNFPFFVLFFFFCFNSNQSWGLMSSNQQPQFFKLMKNSLFGDALSTWNVYDVGTNYCNFTGVRC
DGQGLVTDLDLSGLSLSGIFPDGVCSYFPNLRV
LRLSHNHLNKSSSFLNTIPNCSLLRDLNMSSVYLKGTLPDFSQMKSLRVIDMSWNHFTGSFPLSIFNLTDLE
YLNFNENPELDLWTLPDSVSKLTKLTHM
LLMTCMLHGNIPRSIGNLTSLVDLELSGNFLSGEIPKEIGNLSNLRQLELYYNYHLTGSIPEEIGNLKNLTDIDI
SVSRLTGSIPDSICSLPNLRVLQLY
NNSLTGEIPKSLGNSKTLKILSLYDNYLTGELPPNLGSSSPMIALDVSENRLSGPLPAHVCKSGKLLYFLVLQ
NRFTGSIPETYGSCKTLIRFRVASNRL
VGTIPQGVMSLPHVSIIDLAYNSLSGPIPNAIGNAWNLSELFMQSNRISGVIPHELSHSTNLVKLDLSNNQL
SGPIPSEVGRLRKLNLLVLQGNHLDSSI
PDSLSNLKSLNVLDLSSNLLTGRIPENLSELLPTSINFSSNRLSGPIPVSLIRGGLVESFSDNPNLCIPPTAGSSD
LKFPMCQEPHGKKKLSSIWAILVS
VFILVLGVIMFYLRQRMSKNRAVIEQDETLASSFFSYDVKSFHRISFDQREILESLVDKNIVGHGGSGTVYR
VELKSGEVVAVKKLWSQSNKDSASEDKM
HLNKELKTEVETLGSIRHKNIVKLFSYFSSLDCSLLVYEYMPNGNLWDALHKGFVHLEWRTRHQIAVGVA
QGLAYLHHDLSPPIIHRDIKSTNILLDVNY
QPKVADFGIAKVLQARGKDSTTTVMAGTYGYLAPEYAYSSKATIKCDVYSFGVVLMELITGKKPVDSCFGE
NKNIVNWVSTKIDTKEGLIETLDKRLSES
SKADMINALRVAIRCTSRTPTIRPTMNEVVQLLIDATPQGGPDMTSKPTTKIKDSIVSDHLTQTRL*
>AT5G61480
MKKKNISPSLVLHPLLLLLLPFFAFNSLALKFSPQLLSLLSLKTSLSGPPSAFQDWKVPVNGQNDAVWCSW
SGVVCDNVTAQVISLDLSHRNLSGRIPIQ

IRYLSSLLYLNLSGNSLEGSFPTSIFDLTKLTTLDISRNSFDSSFPPGISKLKFLKVFNAFSNNFEGLLPSDVSRL
RFLEELNFGGSYFEGEIPAAYGGL
QRLKFIHLAGNVLGGKLPPRLGLLTELQHMEIGYNHFNGNIPSEFALLSNLKYFDVSNCSLSGSLPQELGNL
SNLETLFLFQNGFTGEIPESYSNLKSLK
LLDFSSNQLSGSIPSGFSTLKNLTWLSLISNNLSGEVPEGIGELPELTTLFLWNNNFTGVLPHKLGSNGKLET
MDVSNNSFTGTIPSSLCHGNKLYKLIL
FSNMFEGELPKSLTRCESLWRFRSQNNRLNGTIPIGFGSLRNLTFVDLSNNRFTDQIPADFATAPVLQYLN
LSTNFFHRKLPENIWKAPNLQIFSASFSN
LIGEIPNYVGCKSFYRIELQGNSLNGTIPWDIGHCEKLLCLNLSQNHLNGIIPWEISTLPSIADVDLSHNLLTG
TIPSDFGSSKTITTFNVSYNQLIGPI
PSGSFAHLNPSFFSSNEGLCGDLVGKPCNSDRFNAGNADIDGHHKEERPKKTAGAIVWILAAAIGVGFFV
LVAATRCFQKSYGNRVDGGGRNGGDIGPWK
LTAFQRLNFTADDVVECLSKTDNILGMGSTGTVYKAEMPNGEIIAVKKLWGKNKENGKIRRRKSGVLAEV
DVLGNVRHRNIVRLLGCCTNRDCTMLLYEY
MPNGSLDDLLHGGDKTMTAAAEWTALYQIAIGVAQGICYLHHDCDPVIVHRDLKPSNILLDADFEARVA
DFGVAKLIQTDESMSVVAGSYGYIAPEYAYT
LQVDKKSDIYSYGVILLEIITGKRSVEPEFGEGNSIVDWVRSKLKTKEDVEEVLDKSMGRSCSLIREEMKQM
LRIALLCTSRSPTDRPPMRDVLLILQEA
KPKRKTVGDNVIVVGDVNDVNFEDVCSVDVGHDVKCQRIGV*
>AT5G62230
MKEKMQRMVLSLAMVGFMVFGVASAMNNEGKALMAIKGSFSNLVNMLLDWDDVHNSDLCSWRGV
FCDNVSYSVVSLNLSSLNLGGEISPAIGDLRNLQSI
DLQGNKLAGQIPDEIGNCASLVYLDLSENLLYGDIPFSISKLKQLETLNLKNNQLTGPVPATLTQIPNLKRLD
LAGNHLTGEISRLLYWNEVLQYLGLRG
NMLTGTLSSDMCQLTGLWYFDVRGNNLTGTIPESIGNCTSFQILDISYNQITGEIPYNIGFLQVATLSLQGN
RLTGRIPEVIGLMQALAVLDLSDNELVG
PIPPILGNLSFTGKLYLHGNMLTGPIPSELGNMSRLSYLQLNDNKLVGTIPPELGKLEQLFELNLANNRLVG
PIPSNISSCAALNQFNVHGNLLSGSIPL
AFRNLGSLTYLNLSSNNFKGKIPVELGHIINLDKLDLSGNNFSGSIPLTLGDLEHLLILNLSRNHLSGQLPAEF
GNLRSIQMIDVSFNLLSGVIPTELGQ
LQNLNSLILNNNKLHGKIPDQLTNCFTLVNLNVSFNNLSGIVPPMKNFSRFAPASFVGNPYLCGNWVGSI
CGPLPKSRVFSRGALICIVLGVITLLCMIF
LAVYKSMQQKKILQGSSKQAEGLTKLVILHMDMAIHTFDDIMRVTENLNEKFIIGYGASSTVYKCALKSSR
PIAIKRLYNQYPHNLREFETELETIGSIR
HRNIVSLHGYALSPTGNLLFYDYMENGSLWDLLHGSLKKVKLDWETRLKIAVGAAQGLAYLHHDCTPRII
HRDIKSSNILLDENFEAHLSDFGIAKSIPA
SKTHASTYVLGTIGYIDPEYARTSRINEKSDIYSFGIVLLELLTGKKAVDNEANLHQLILSKADDNTVMEAVD
PEVTVTCMDLGHIRKTFQLALLCTKRN
PLERPTMLEVSRVLLSLVPSLQVAKKLPSLDHSTKKLQQENEVRNPDAEASQWFVQFREVISKSSI*
>AT5G63930
MVKEMMKLAVFFISLLLILLISETTGLNLEGQYLLEIKSKFVDAKQNLRNWNSNDSVPCGWTGVMCSNYS
SDPEVLSLNLSSMVLSGKLSPSIGGLVHLK
QLDLSYNGLSGKIPKEIGNCSSLEILKLNNNQFDGEIPVEIGKLVSLENLIIYNNRISGSLPVEIGNLLSLSQLVT
YSNNISGQLPRSIGNLKRLTSFRA
GQNMISGSLPSEIGGCESLVMLGLAQNQLSGELPKEIGMLKKLSQVILWENEFSGFIPREISNCTSLETLALY
KNQLVGPIPKELGDLQSLEFLYLYRNG

LNGTIPREIGNLSYAIEIDFSENALTGEIPLELGNIEGLELLYLFENQLTGTIPVELSTLKNLSKLDLSINALTGPI
PLGFQYLRGLFMLQLFQNSLSGT
IPPKLGWYSDLWVLDMSDNHLSGRIPSYLCLHSNMIILNLGTNNLSGNIPTGITTCKTLVQLRLARNNLVG
RFPSNLCKQVNVTAIELGQNRFRGSIPRE
VGNCSALQRLQLADNGFTGELPREIGMLSQLGTLNISSNKLTGEVPSEIFNCKMLQRLDMCCNNFSGTLP
SEVGSLYQLELLKLSNNNLSGTIPVALGNL
SRLTELQMGGNLFNGSIPRELGSLTGLQIALNLSYNKLTGEIPPELSNLVMLEFLLLNNNNLSGEIPSSFANL
SSLLGYNFSYNSLTGPIPLLRNISMSS
FIGNEGLCGPPLNQCIQTQPFAPSQSTGKPGGMRSSKIIAITAAVIGGVSLMLIALIVYLMRRPVRTVASSA
QDGQPSEMSLDIYFPPKEGFTFQDLVAA
TDNFDESFVVGRGACGTVYKAVLPAGYTLAVKKLASNHEGGNNNNVDNSFRAEILTLGNIRHRNIVKLHG
FCNHQGSNLLLYEYMPKGSLGEILHDPSCN
LDWSKRFKIALGAAQGLAYLHHDCKPRIFHRDIKSNNILLDDKFEAHVGDFGLAKVIDMPHSKSMSAIAGS
YGYIAPEYAYTMKVTEKSDIYSYGVVLLE
LLTGKAPVQPIDQGGDVVNWVRSYIRRDALSSGVLDARLTLEDERIVSHMLTVLKIALLCTSVSPVARPSM
RQVVLMLIESERSEGEQEHLDTEELTQTT
TP*
>AT5G65700
MKLFLLLLFLLHISHTFTASRPISEFRALLSLKTSLTGAGDDKNSPLSSWKVSTSFCTWIGVTCDVSRRHVTSL
DLSGLNLSGTLSPDVSHLRLLQNLSL
AENLISGPIPPEISSLSGLRHLNLSNNVFNGSFPDEISSGLVNLRVLDVYNNNLTGDLPVSVTNLTQLRHLHL
GGNYFAGKIPPSYGSWPVIEYLAVSGN
ELVGKIPPEIGNLTTLRELYIGYYNAFEDGLPPEIGNLSELVRFDGANCGLTGEIPPEIGKLQKLDTLFLQVNV
FSGPLTWELGTLSSLKSMDLSNNMFT
GEIPASFAELKNLTLLNLFRNKLHGEIPEFIGDLPELEVLQLWENNFTGSIPQKLGENGKLNLVDLSSNKLTG
TLPPNMCSGNKLETLITLGNFLFGSIP
DSLGKCESLTRIRMGENFLNGSIPKGLFGLPKLTQVELQDNYLSGELPVAGGVSVNLGQISLSNNQLSGPL
PPAIGNFTGVQKLLLDGNKFQGPIPSEVG
KLQQLSKIDFSHNLFSGRIAPEISRCKLLTFVDLSRNELSGEIPNEITAMKILNYLNLSRNHLVGSIPGSISSMQ
SLTSLDFSYNNLSGLVPGTGQFSYF
NYTSFLGNPDLCGPYLGPCKDGVAKGGHQSHSKGPLSASMKLLLVLGLLVCSIAFAVVAIIKARSLKKASES
RAWRLTAFQRLDFTCDDVLDSLKEDNII
GKGGAGIVYKGVMPNGDLVAVKRLAAMSRGSSHDHGFNAEIQTLGRIRHRHIVRLLGFCSNHETNLLVY
EYMPNGSLGEVLHGKKGGHLHWDTRYKIALE
AAKGLCYLHHDCSPLIVHRDVKSNNILLDSNFEAHVADFGLAKFLQDSGTSECMSAIAGSYGYIAPEYAYTL
KVDEKSDVYSFGVVLLELVTGRKPVGEF
GDGVDIVQWVRKMTDSNKDSVLKVLDPRLSSIPIHEVTHVFYVAMLCVEEQAVERPTMREVVQILTEIPK
LPPSKDQPMTESAPESELSPKSGVQSPPDL
LNL*
>AT5G65710
MLTNTNLFFFLSLLLLSCFLQVSSNGDAEILSRVKKTRLFDPDGNLQDWVITGDNRSPCNWTGITCHIRKG
SSLAVTTIDLSGYNISGGFPYGFCRIRTL
INITLSQNNLNGTIDSAPLSLCSKLQNLILNQNNFSGKLPEFSPEFRKLRVLELESNLFTGEIPQSYGRLTALQ
VLNLNGNPLSGIVPAFLGYLTELTRL
DLAYISFDPSPIPSTLGNLSNLTDLRLTHSNLVGEIPDSIMNLVLLENLDLAMNSLTGEIPESIGRLESVYQIEL
YDNRLSGKLPESIGNLTELRNFDVS

QNNLTGELPEKIAALQLISFNLNDNFFTGGLPDVVALNPNLVEFKIFNNSFTGTLPRNLGKFSEISEFDVSTN
RFSGELPPYLCYRRKLQKIITFSNQLS
GEIPESYGDCHSLNYIRMADNKLSGEVPARFWELPLTRLELANNNQLQGSIPPSISKARHLSQLEISANNFS
GVIPVKLCDLRDLRVIDLSRNSFLGSIP
SCINKLKNLERVEMQENMLDGEIPSSVSSCTELTELNLSNNRLRGGIPPELGDLPVLNYLDLSNNQLTGEIP
AELLRLKLNQFNVSDNKLYGKIPSGFQQ
DIFRPSFLGNPNLCAPNLDPIRPCRSKRETRYILPISILCIVALTGALVWLFIKTKPLFKRKPKRTNKITIFQRVG
FTEEDIYPQLTEDNIIGSGGSGLV
YRVKLKSGQTLAVKKLWGETGQKTESESVFRSEVETLGRVRHGNIVKLLMCCNGEEFRFLVYEFMENGSL
GDVLHSEKEHRAVSPLDWTTRFSIAVGAAQ
GLSYLHHDSVPPIVHRDVKSNNILLDHEMKPRVADFGLAKPLKREDNDGVSDVSMSCVAGSYGYIAPEYG
YTSKVNEKSDVYSFGVVLLELITGKRPNDS
SFGENKDIVKFAMEAALCYPSPSAEDGAMNQDSLGNYRDLSKLVDPKMKLSTREYEEIEKVLDVALLCTSS
FPINRPTMRKVVELLKEKKSLE*
>GRMZM2G039431
MPQRLGATTAARLVALLVCLSPALLAPCRGVNEQGQALLRWKGSSARGALDSSWRAADATPCRWLGV
GCDARGDVTSLTIRSVDLGGALPAGPELRPLSS
SLKTLVLSGTNLTGAIPRELGDLAELTTLDLSKNQLSGAIPHELCRLTKLQSLALNSNSLRGAIPGDIGNLTSL
TTLALYDNQLSGAIPASIGNLKKLQV
LRAGGNQALKGPLPPEIGRCTDLTMLGLAETGLSGSLPETIGQLKKIQTIAIYTAMLTGSIPESIGNCTELTSL
YLYQNSLSGPIPPQLGQLRKLQTVLL
WQNQLVGTIPPEIANCKDLVLIDLSLNSLTGPIPSSFGTLPNLQQLQLSTNKLTGVIPPELSNCTSLTDVEVD
NNELSGEIGIDFSRLRNLTLFYAWQNR
LTGPVPAGLAQCEGLQSLDLSYNNLTGPVPGDVFALQNLTKLLLLNNDLSGFIPPEIGNCTNLYRLRLNDN
RLSGTIPAEIGKLKNLNFLDLGSNRLVGP
LPAALSGCDNLEFMDLHSNALSGALPDELPRSLQFVDISDNKLTGMLGPGIGLLPELTKLNLGMNRISGGI
PPELGSCEKLQLLDLGDNALSGGIPPELG
KLPSLEISLNLSCNRLSGEIPAQFGELDKLGSLDISYNQLSGSLAPLARLENLVMLNISYNTFSGDLPDTPFFQ
KLPLSDIAGNHLLVVGAGGDEASRHA
AVSALKLAMTILVVVSALLLLTATYVLARSRRRNGAIHGHGADETWEVTLYQKLDFSVDEVVRALTSANVI
GTGSSGVVYRVALPNGDSLAVKKMWSSDE
AGAFRNEISALGSIRHRNIVRLLGWGANRSTKLLFYAYLPNGSLSGFIHRGGVKGAADWGARYDVALGVA
HAVAYLHHDCLPAILHGDIKAMNVLLGPRN
EPYLADFGLARVLSGAVASGSAKLDSSKAPRIAGSYGYIAPEYASMQRITEKSDVYSFGVVVLEILTGRHPL
DPTLPGGTHLVQWVREHVRAKRATAELL
DPRLRGKPEAQVQEMLQVFSVAMLCIAHRAEDRPAMKDVVALLKEIRRPAERSDEGKEQPACNTAAAA
TAAAAEPLDGQAQRSPPRSPLPKGGSSSCSFA
MSDYSS*
>GRMZM2G043584
MAMAPSTAAASTLLPVLLLIATATQCTAADSFSSQDAAALLNLSAAVADPSGYLSTHWTPDTAVCSWPR
VSCDATDTRVISLDLSGLNLSGPIPAAALSS
FPYLQSLNLSNNILNSTAFPDEIIASLKSLRVLDLYNNNLTGSLPAALPNLTDLVHVHLGGNFFSGSIPRSYG
QWSRIRYLALSGNELTGEIPEELGNLT
TLRELYLGYYNNFTGGIPPELGRLRALVRLDMANCGISEEIPPELANLTSLDTLFLQINALSGRLPTEIGAMG
SLKSLDLSNNLFVGEIPASFASLKNLT
LLNLFRNRLAGEIPEFIGDLPNLEVLQLWENNFTGGIPTNLGVAATRLRIVDVSTNKLTGVLPSELCAGQRL
ETFIALGNSLFGDVPDGLAGCPSLTRIR

LGENFLNGTIPAKLFTLPNLTQVELHNNLLSGELRLDGGKVSSSIGELSLFNNRLTGQVPTGIGGLLGLQKLL
LAGNMLSGELPPEVGKLQQLSKADLSG
NLLSGAVPPAIGRCRLLTFLDISSNKLSGSIPPELGSLRILNYLNVSHNALQGEIPPAIAGMQSLTAVDFSYN
NLSGEVPSTGQFGYFNATSFAGNAGLC
GAFLSPCRSVGVATSALGSLSSTSKLLLVLGLLALSVVFAGAAVLKARSLKRSAEARAWRLTAFQRLDFAVD
DVLDCLKEENVIGKGGSGIVYKGAMPGG
AVVAVKRLPAIGRAGAAHDDYGFSAEIQTLGRIRHRHIVRLLGFAANRETNLLVYEYMPNGSLGEVLHGK
KGGHLQWATRFKIAVEAAKGLCYLHHDCSP
PILHRDVKSNNILLDADFEAHVADFGLAKFLRGNAGGSECMSAIAGSYGYIAPEYAYTLKVDEKSDVYSFG
VVLLELIAGRKPVGEFGDGVDIVHWVRTV
TGSSKEGVMKIADPRLSTVPLYELTHVFYVAMLCVAEQSVERPTMREVVQILADMPGSTSTTSIDVPLVIE
PKEEDGGPEKKQQQQQEGPHDSPPQQDLL
SI*
>GRMZM2G082191
MEHRALLLGVALAFLLASGSQGLNHEGWLLLALKSQMNDTLHHLDNWDARDLTPCIWKGVSCSSTPNP
VVVSLDLSNMNLSGTVAPSIGSLSELTLLDLS
FNGFYGTIPPEIGNLSKLEVLNLYNNSFVGTIPPELGKLDRLVTFNLCNNKLHGPIPDEVGNMTALQELVGY
SNNLTGSLPRSLGKLKNLKNIRLGQNLI
SGNIPVEIGACLNITVFGLAQNKLEGPLPKEIGRLTLMTDLILWGNQLSGVIPPEIGNCTSLSTIALYDNNLV
GPIPATIVKITNLQKLYLYRNSLNGTI
PSDIGNLSLAKEIDFSENFLTGGIPKELADIPGLNLLYLFQNQLTGPIPTELCGLKNLSKLDLSINSLNGTIPVG
FQYMRNLIQLQLFNNMLSGNIPPRF
GIYSRLWVVDFSNNSITGQIPKDLCRQSNLILLNLGSNMLTGNIPRGITNCKTLVQLRLSDNSLTGSFPTDL
CNLVNLTTVELGRNKFSGPIPPQIGSCK
SLQRLDLTNNYFTSELPREIGNLSKLVVFNISSNRLGGNIPLEIFNCTVLQRLDLSQNSFEGSLPNEVGRLPQ
LELLSFADNRLTGQIPPILGELSHLTA
LQIGGNQLSGEIPKELGLLSSLQIALNLSYNNLSGDIPSELGNLALLESLFLNNNKLMGEIPTTFANLSSLLEL
NVSYNYLSGALPPIPLFDNMSVTCFI
GNKGLCGGQLGRCGSRPSSSSQSSKSVSPPLGKIIAIVAAVIGGISLILIAIIVHHIRKPMETVAPLQDKQPFP
ACSNVHVSAKDAYTFQELLTATNNFD
ESCVIGRGACGTVYRAILKAGQTIAVKKLASNREGSNTDNSFRAEIMTLGKIRHRNIVKLYGFVYHQGSNLL
LYEYMSRGSLGELLHGQSSSSLDWETRF
LIALGAAEGLSYLHHDCKPRIIHRDIKSNNILLDENFEAHVGDFGLAKVIDMPYSKSMSAIAGSYGYIAPEYA
YTMKVTEKCDIYSYGVVLLELLTGRAP
VQPLELGGDLVTWVKNYIKDNCLGPGILDKKMDLQDQSVVDHMIEVMKIALVCTSLTPYERPPMRHVV
VMLSESKDRTRVSSASSPASDDSSKKDDS*
>GRMZM2G082855
MAARSSAAAVAVLLLAAAGAAAAAGGAEGDGDGQALMAVKAGFRNAANALADWDGGRDHCAWRG
VACDAASFAVVGLNLSNLNLGGEISPAIGQLKSLQF
VDLKLNKLTGQIPDEIGDCVSLKYLDLSGNLLYGDIPFSISKLKQLEDLILKNNQLTGPIPSTLSQIPNLKTLDL
AQNKLTGDIPRLIYWNEVLQYLGLR
GNSLTGTLSPDMCQLTGLWYFDIRGNNLTGTIPEGIGNCTSFEILDISYNQISGEIPYNIGYLQVATLSLQGN
RLIGKIPEVIGLMQALAVLDLSENELV
GPIPPILGNLSYTGKLYLHGNKLTGHIPPELGNMSKLSYLQLNDNELVGTIPAELGKLTELFELNLANNNLEG
HIPANISSCSALNKFNVYGNRLNGSIP
AGFQKLESLTYLNLSSNSFKGQIPSELGHIVNLDTLDLSYNEFSGPVPPTIGDLEHLLELNLSKNHLTGSVPAE
FGNLRSVQVIDMSSNNLSGYLPEELG

QLQNLDSLILNNNSLAGEIPAQLANCFSLVSLNLSYNNFSGHVPSSKNFSKFPMESFMGNLMLHVYCQDS
SCGHSHGTKVSISRTAVACMILGFVILLCI
VLLAIYKTNQPQLPEKASDKPVQGPPKLVVLQMDMAVHTYEDIMRLTENLSEKYIIGYGASSTVYRCDLKS
GKAIAVKRLYSQYNHSLREFETELETIGS
IRHRNLVSLHGFSLSPHGNLLFYDYMENGSLWDLLHGPSKKVKLDWDTRLRIAVGAAQGLAYLHHDCNP
RIVHRDVKSSNILLDGSFEAHLSDFGIAKCV
PAAKSHASTYVLGTIGYIDPEYARTSRLNEKSDVYSFGVVLLELLTGRKAVDNESNLHQLILSKADDDTVME
AVDPEVSVTCTDMNLVRKAFQLALLCTK
RHPADRPTMHEVARVLLSLLPPAAKPPASKAAAASAAAGDYTRFLATAADLRRGGVADDDTGDNSSSDE
QWFVRFGEVISKHTLS*
>GRMZM2G112309
MPTPPSAPARFLLASLLFAILALAAVSANAAAPPSPAEALLAWKSSLGDPAMLSTWTNATQVSICTTWRG
VACDAAGRVVSLRLRGLGLTGGLDALDPAA
FPSLTSLDLKDNNLAGAIPPSLSQLRTLATLDLGSNGLNGTIPPQLGDLSGLVELRLFNNNLAGAIPNQLSKL
PKIVQMDLGSNYLTSVPFSPMPTVEFL
SLSVNYINGSFPEFVLRSGNVTYLDLSQNGFSGPIPDALPERLPNLRWLNLSANAFSGRIPASLARLTRLRDL
HLGGNNLTGGVPDFLGSMSQLRVLELG
SNPLGGALPPVLGQLKMLQQLDVKNASLVSTLPPELGGLSNLDFLDLSINQLYGSLPASFAGMQRMREFG
ISSNNLTGEIPGQLFMSWPELISFQVQTNS
LRGKIPPELGKVTKIRFLYLFSNNLTGEIPSELGRLVNLVELDLSVNSLIGPIPSTFGNLKQLTRLALFFNELTGK
IPSEIGNMTALQTLDLNTNNLEGE
LPPTISLLRNLQYLSVFDNNMTGTVPPDLGAGLALTDVSFANNSFSGELPQRLCDGFALTNFTAHHNNFS
GKLPPCLKNCSGLYRVRLEGNHFTGDISEA
FGVHPIMDYLDISGNKLTGRLSDDWGQCTKLTRLKMDGNSISGAIPEAFGNITSLQDLSLAANNLTGAIPP
ELGDLNFLFDLNLSHNSFSGPIPTSLGHS
SKLQKVDLSENMLNGTIPVSVGNLGSLTYLDLSKNKLSGQIPSEIGNLFQLQALLDLSSNSLSGPIPSNLVKL
SNLQKLNLSRNELNGSIPASFSRMSSL
ETVDFSYNQLTGEVPSGNVFQNSSAEAYIGNLGLCGDAQGIPSCGRSSSPPGHHERRLIAIVLSVVGTVLL
AAIVVVACLILACRRRPRERKVLEASTSD
PYESVIWEKGGNITFLDIVNATDGFSEVFCIGKGGFGSVYKAELPGGQVVAVKRFHVAETGDISEASRKSF
ENEVRALTEVRHRNIVKLHGFCTSGGYMH
LVYEYLERGSLGKTLYGEDGKRKLGWGTRVKVVQGVAHALAYLHHDGSQPIVHRDITVSNILLESEFEPRL
SDFGTAKLLGSASTNWTSVAGSYGYMAPE
LAYTMNVTEKCDVYSFGVVALEVMMGKHPGDLLSSLPAISSSSSGEGLLLQDILDQRLEPPTGDLAEQVVL
VVRIALACTRANPDSRPSMRSVAQEMSAR
TQASHLSEPFRQITVSKLTDYQK*
>GRMZM2G132763
MSCAAALSLLVALAASVAPAAASQGAGNGDGDAAVLRAFLVSLPPSSQRILLPSWNATNSSSSTGSSHCA
FRGVECTAAGAVAAVNLSGLALSGALAASA
PGLCALPALAALDLSLNSFTGAVPAALAACSALATLDLSNNSLSGAVPRELAALPALTDLRLSGNGLTGPVP
EFPARCGLRYLSLYGNRISGALPRSLGN
CVNLTVLFLSSNRIGGALPDVFGSLPMLQKLYLDSNLFAGALPESVGELGSLERFVASTNCFNGSIPASIGRC
GSLTTLLLHNNQFTGPIPASIGNLSRL
QWLTIKDTFVTGAIPPEIGRCQELVILDLQNNNLTGTIPPELAELKKLRSLSLYRNMLHGPVPAALWQMPE
LEKLALYNNSLSGEIPEEINHMRNLRELL
LAFNNFTGELPQGLGSNTTHGLVWVDVMGNHFHGAIPPGLCTGGQLAILDLALNRFSGGIPSEIIKCQSL
WRARLANNLFSGSFPSDLGINTGWSYVELG

GNRFDGRIPSVLGSWRNLTVLDLSRNSFSGPIPPELGALAHLGDLNLSSNKLSGRIPHELGNCRGLVRLDLE
NNLLNGSIPAEIVSLGSLQHLVLGGNKL
SGEIPDAFTSTQGLLELQLGGNSLEGAVPWSLGKLQFISQIINMSSNMLSGTIPSSLGNLRMLEMLDLSEN
SLSGPIPSQLSNMVSLSAANVSFNRLSGP
LPVGWANKLPADGFLGNPQLCVRPEDAACSKNQYRSRTRRNTRIIVALLLSSLAVMASGLCAVRYAVKTS
RRRLLAKRVSVRGLDATTTEELPEDLSYDD
IIRATDNWSEKYVIGRGRHGTVYRTELAPGRRWAVKTVDLSRVKFPIEMKILNMVRHRNIVKMEGYCIRG
NFGVILSEYMPRGTLFELLHGRKPQVVALD
WKARHQIALGAAQGLSYLHHDCVPMVVHRDVKSSNILMDADLVPKIADFGMGKIVGDEDADATVSVV
VGTLGYIAPEHGYNTRLTEKSDVYSYGVVLLEL
LCRRMPVDPAFGDGVDIVAWMRLNLKHADCCSVMTFLDEEIMYWPEDEKAKALDVLDMAISCTQVAF
ESRPSMREVVGALMRIDDQYI*
>GRMZM2G148702
MAHHRRARLFSAAVVVAAALVALSVGTAQPAAALSPDGKALLSLLPGAAPSPVLPSWDPRAATPCSWQ
GVTCSPQSRVVSLSLPDTFLNLSSLPPALATL
SSLQLLNLSACNVSGAIPPSYASLSALRVLDLSSNALTGDIPDGLGALSGLQFLLLNSNRLTGGIPRSLANLSA
LQVLCVQDNLLNGTIPASLGALAALQ
QFRVGGNPALSGPIPASLGALSNLTVFGAAVTALSGPIPEEFGSLVNLQTLALYDTSVSGSIPAALGGCVEL
RNLYLHMNKLTGPIPPELGRLQKLTSLL
LWGNALSGKIPPELSNCSALVVLDLSGNRLTGEVPGALGRLGALEQLHLSDNQLTGRIPPELSNLSSLTALQ
LDKNGFSGAIPPQLGELKALQVLFLWGN
ALSGAIPPSLGNCTDLYALDLSKNRFSGGIPDEVFGLQKLSKLLLLGNELSGPLPPSVANCLSLVRLRLGENK
LVGQIPREIGKLQNLVFLDLYSNRFTG
KLPGELANITVLELLDVHNNSFTGGIPPQFGELMNLEQLDLSMNELTGEIPASFGNFSYLNKLILSGNNLSG
PLPKSIRNLQKLTMLDLSNNSFSGPIPP
EIGALSSLGISLDLSLNKFVGELPDEMSGLTQLQSLNLASNGLYGSISVLGELTSLTSLNISYNNFSGAIPVTPF
FKTLSSNSYIGNANLCESYDGHSCA
ADTVRRSALKTVKTVILVCGVLGSVALLLVVVWILINRSRKLASQKAMSLSGACGDDFSNPWTFTPFQKLN
FCIDHILACLKDENVIGKGCSGVVYRAEM
PNGDIIAVKKLWKAGKDEPIDAFAAEIQILGHIRHRNIVKLLGYCSNRSVKLLLYNYIPNGNLLELLKENRSLD
WDTRYKIAVGTAQGLAYLHHDCIPAI
LHRDVKCNNILLDSKYEAYLADFGLAKLMNSPNYHHAMSRIAGSYGYIAPEYAYTSNITEKSDVYSYGVVLL
EILSGRSAIEPVLGEASLHIVEWAKKKM
GSYEPAVNILDPKLRGMPDQLVQEMLQTLGVAIFCVNTAPHERPTMKEVVALLKEVKSPPEEWAKTSQQ
PLIKPGSQQG*
>GRMZM2G168603
MEARVTVLALLLVTVWSISCTRAGAAGDERAALLALKAGFVDSLGALADWTDGAKAAPHCRWTGVRCN
AAGLVDELDLSGKNLSGKVTGDVLRLPSLAVL
NLSSNAFATALPKSLAPLSSLRVLDVSQNSFEGAFPAGLGACAGLDTVNASGNNFVGALPADLANATSLQ
TVDLRGSFFGGGIPAAYRSLTKLRFLGLSG
NNITGKIPPELGELESLESLIIGYNALEGTIPPELGGLANLQYLDLAVGNLDGPIPAELGRLPALTALYLYKNNL
EGKIPPELGNISTLVFLDLSDNSLT
GPIPDEIAQLSHLRLLNLMCNHLDGTVPATIGDMPSLEVLELWNNSLTGQLPASLGNSSPLQWVDVSSNS
FTGPVPAGICDGKELAKLIMFNNGFTGGIP
AGLASCASLVRVRMQSNRLTGTIPVGFGKLPSLQRLELAGNDLSGEIPGDLASSTSLSFIDLSHNHLQYTLP
SSLFTIPTLQSFLASDNLISGELPDQFQ

DCPALAALDLSNNRLAGAIPSSLASCQRLVKLNLRHNRLTGEIPKALAMMPAMAILDLSSNSLTGHIPENF
GSSPALETLNLSYNNLTGPVPGNGVLRSI
NPDELAGNAGLCGGVLPPCFGSRDTGVAAARPRGSARLRRIAASWLAAMLAAVAAFTALVGGRYAYRR
WYAGRCDDESLGAESGAWAWRLTAFQRLGFTS
ADVLACVKEANVVGMGATGVVYKAELPRARAVIAVKKLWRPAPVDGDAASEPTADVLKEVALLGRLRH
RNIVRLLGYVHNGAADAMMLYEFMPNGSLWEA
LHGPPGKRALLDWVSRYDVAAGVAQGLAYLHHDCHPPVIHRDIKSNNILLDADMEARIADFGLARALAR
SNESVSVVAGSYGYIAPEYGYTLKVDQKSDI
YSYGVVLMELITGHRAVEAEFGEGQDIVGWVRDKIRSNTVEEHLDPHVGGRCAHVREEMLLVLRIAVLCT
AKAPRDRPSMRDVITMLGEAKPRRKSGSSS
GGGTASGKDSAAPAVAVDRDRPVFSTTPDSDYA*
>GRMZM2G452142
MGGSRWLLHFLLVSVLLHVVRSLESQACHPADLRALLDFSGGWDSKAAGLVGWGPGAAACCSWTGVA
CDLGRVVALDLSNRSLHGVISPAVASLDGLAAL
NLSRNALRGAAPEALARLPRLRALDLSANALSGPFPAAGFPAIEELNISFNSFDGPHPAFPAAANLTALDVS
ANNFSGGINSSALCLSPLQVLRFSGNAL
SGEIPSGLSQCRALTDLSLDGNCFTGNVPGDLYTLPNLRRLSLQENQLTGNLGSDLGNLSQIVQLDLSYNK
FTGSIPDVFGNMRWLESVNLATNRLDGEL
PASLSSCPLLRVISLRNNSLSGEIAIDFSRLPNLNTFDIGTNYLSGAIPPGIAVCTELRTLNLARNKLVGEIPESF
KELTSLSYLSLTGNSFTNLASALQ
VLQHLPNLTSLVLTRNFRGGETIPVDGISGFKSMQVLVLANCLLTGVIPPWLQSLGSLNVLDISWNKLNGN
IPPWLGKLDNLFYIDLSNNSFSGELPISF
TQMRSLTSTNGSSERSPTEDLPLFIKRNSTGKGLQYNQVSSFPPSLILSNNLLVGPVLSSFGYLVKLHVLDLS
WNNFSGPIPDELSNMSSLEVLNLAHND
LDGTIPSSLTRLNFLSMFDVSYNNLTGDIPTGGQFSTFAPENFDGNPALCLRNSSCAEKDSSVGAAGHSNK
KRKAATVALGLGTAVGVLLLVLCAYVIVS
RIVHSRMQERNPKAVANAEDSECSSNSCLVLLFQNNKELSIEDILKSTNNFDQAYIVGCGGFGLVYRSTLP
DGRRVAIKRLSGDYSQIEREFQAEVETLS
RAQHENLVLLQGYCKVGSDRLLIYSYMENGSLDYWLHERADDSGVLLDWRKRLRIAQGSARGLAYLHMS
CDPHILHRDIKSSNILLDDNFEAHLADFGLA
RLICAYETHVTTDVVGTLGYIPPEYGQSPVATYKGDVYSFGIVLLELLTGRRPVDMCRPKGTRDVVSWVLR
MKEEGREAEVFHPSIHHEDNQGQLVRILD
IACLCVTAAPKSRPTSQQLVAWLDDIAEG*
>GRMZM2G463904
MQALAVLDLSFNELSGPIPSILGNLTYTEKLYLQGNRLTGLIPPELGNMSTLHYLELNDNLLTGFIPPDLGKL
TELFELNLANNNLIGPIPENLSSCANL
ISFNAYGNKLNGTIPRSFHKLESLTYLNLSSNHLSGALPIEVARMRNLDTLDLSCNMITGSIPSAIGKLEHLLR
LNLSKNNVAGHIPAEFGNLRSIMEID
LSYNHLSGLIPQEVGMLQNLILLKLESNNITGDVSSLIYCLSLNILNVSYNHLYGTVPTDNNFSRFSPDSFLG
NPGLCGYWLHSASCTQLSNAEQMKRSS
SAKASMFAAIGVGAVLLVIMLVILVVICWPHNSPVLKDVSVNKPDNLASASNNIHPKLVILHMNMALYVY
DDIMRMTENLSEKYIIGYGASSTVYRCDLK
NCKPIAIKKLYAHYPQSLKEFETELETVGSIKHRNLVSLQGYSLSPSGNLLFYDYMENGSLWDILHAASSKKK
KLDWEARLKIALGAAQGLAYLHHECSP
RIIHRDVKSKNILLDKDYEAHLADFGIAKSLCVSKTHTSTYVMGTIGYIDPEYARTSRINEKSDVYSYGIVLLEL
LTGKKPVDDECNLHHLILSKAAENT

VMETVDQDITDTCKDLGEVKKVFQLALLCSKRQPSDRPTMHEVARVLDSLVCPAGPPPKQAQAQAQAQ
ASEKPSTTAPSYVSEYVGLRGGGGGSALSCTN
SSSASDAELFMKFGEVISRSTE*
>GRMZM5G809695
MPVRSSVAMTTTAARALAALVLVTAAAAAAAVADDGAALVEIKKSFRNVGNVLYDWAGDDYCSWRGV
LCDNVTFAVAALNLSGLNLEGEISPAVGSLKSL
VSIDLKSNGLSGQIPDEIGDCSSLRTLDFSFNNLDGDIPFSISKLKHLENLILKNNQLIGAIPSTLSQLPNLKILD
LAQNKLTGEIPRLIYWNEVLQYLG
LRGNHLEGSLSPDMCQLTGLWYFDVKNNSLTGAIPDTIGNCTSFQVLDLSYNRFTGPIPFNIGFLQVATLS
LQGNKFTGPIPSVIGLMQALAVLDLSYNQ
LSGPIPSILGNLTYTEKLYMQGNRLTGSIPPELGNMSTLHYLELNDNQLTGSIPPELGRLTGLFDLNLANNH
LEGPIPDNLSSCVNLNSFNAYGNKLNGT
IPRSLRKLESMTYLNLSSNFISGSIPIELSRINNLDTLDLSCNMMTGPIPSSIGNLEHLLRLNLSKNDLVGFIPA
EFGNLRSVMEIDLSYNHLGGLIPQE
LGMLQNLMLLKLENNNITGDVSSLMNCFSLNILNVSYNNLAGAVPTDNNFTRFSHDSFLGNPGLCGYWL
GSSCRSTGHRDKPPISKAAIIGVAVGGLVIL
LMILVAVCRPHHPPAFKDATVSKPVSNGPPKLVILHMNMALHVFDDIMRMTENLSEKYIIGYGASSTVYK
CVLKNCKPVAIKKLYAHYPQSLKEFETELE
TVGSIKHRNLVSLQGYSLSPVGNLLFYDYMESGSLWDVLHEGSSKKNKLDWVTRLRIALGAAQGLAYLHH
DCSPRIIHRDVKSKNILLDKDYEAHLTDFG
IAKSLCVSKTHTSTYVMGTIGYIDPEYARTSRLNEKSDVYSYGIVLLELLTGKKPVDNECNLHHLVTILQSPAF
VELEAGSRFLLFTSLWIPAVLNNC*
>LOC_Os02g02140
MEATVPVLLLVTVLSLILPSGIGAAAAGDERSALLALKAGFVDTVSALADWTDGGKASPHCKWTGVGCN
AAGLVDRLELSGKNLSGKVADDVFRLPALAV
LNISNNAFATTLPKSLPSLPSLKVFDVSQNSFEGGFPAGLGGCADLVAVNASGNNFAGPLPEDLANATSLE
TIDMRGSFFGGAIPAAYRSLTKLKFLGLS
GNNITGKIPPEIGEMESLESLIIGYNELEGGIPPELGNLANLQYLDLAVGNLDGPIPPELGKLPALTSLYLYKN
NLEGKIPPELGNISTLVFLDLSDNAF
TGAIPDEVAQLSHLRLLNLMCNHLDGVVPAAIGDMPKLEVLELWNNSLTGSLPASLGRSSPLQWVDVSS
NGFTGGIPAGICDGKALIKLIMFNNGFTGGI
PAGLASCASLVRVRVHGNRLNGTIPVGFGKLPLLQRLELAGNDLSGEIPGDLASSASLSFIDVSRNHLQYSI
PSSLFTIPTLQSFLASDNMISGELPDQF
QDCPALAALDLSNNRLAGAIPSSLASCQRLVKLNLRRNKLAGEIPRSLANMPALAILDLSSNVLTGGIPENF
GSSPALETLNLAYNNLTGPVPGNGVLRS
INPDELAGNAGLCGGVLPPCSGSRSTAAGPRSRGSARLRHIAVGWLVGMVAVVAAFAALFGGHYAYRR
WYVDGAGCCDDENLGGESGAWPWRLTAFQRLG
FTCAEVLACVKEANVVGMGATGVVYKAELPRARAVIAVKKLWRPAAAAEAAAAAPELTAEVLKEVGLLG
RLRHRNIVRLLGYMHNEADAMMLYEFMPNGS
LWEALHGPPERRTLVDWVSRYDVAAGVAQGLAYLHHDCHPPVIHRDIKSNNILLDANMEARIADFGLAR
ALGRAGESVSVVAGSYGYIAPEYGYTMKVDQ
KSDTYSYGVVLMELITGRRAVEAAFGEGQDIVGWVRNKIRSNTVEDHLDGQLVGAGCPHVREEMLLVLR
IAVLCTARLPRDRPSMRDVITMLGEAKPRRK
SGSSTGSASAKAPTPAPPAVAAVVDKDKPVFTTTPDSDYA*
>LOC_Os02g53720
MILAAACWPHWAQVPKDVSLCKPDIHALPSSNVPPKLVILHMNMAFLVYEDIMRMTENLSEKYIIGYGA
SSTVYKCVLKNCKPVAIKKLYAHYPQSLKEF

ETELETVGSIKHRNLVSLQGYSLSPAGNLLFYDYLENGSLWDVLHAGSSKKQKLDWEARLRIALGAAQGLA
YLHHDCNPRIIHRDVKSKNILLDKDYEAH
LADFGIAKSLCTSKTHTSTYVMGTIGYIDPEYACTSRLNEKSDVYSYGIVLLELLTGKKPVDNECNLHHLILSK
AADNTVMEMVDPDIADTCKDLGEVKK
VFQLALLCSKRQPSDRPTMHEVVRVLDCLVYPDPPSKPALPPALPQSSTVPSYVNEYVSLRGGSTLSCENS
SSASDAELFLKFGEVISQNTE*
>LOC_Os03g56270
MHLRLLLLLVGVAAAAADADADADALLAAKAAMSDPTGALASWGGNGTRTNTTAAAAAHCAWAGVT
CSSRGAVVGLDVSGLNLSGALPAELTGLRGLMRL
SVGANAFSGPIPASLGRLQFLTYLNLSNNAFNGSFPAALARLRGLRVLDLYNNNLTSPLPMEVVQMPLLR
HLHLGGNFFSGEIPPEYGRWGRMQYLAVSG
NELSGKIPPELGNLTSLRELYIGYYNSYSGGLPPELGNLTELVRLDAANCGLSGEIPPELGKLQNLDTLFLQV
NSLAGGIPSELGYLKSLSSLDLSNNVL
TGEIPASFSELKNLTLLNLFRNKLRGDIPDFVGDLPSLEVLQLWENNFTGGVPRRLGRNGRLQLLDLSSNRL
TGTLPPELCAGGKMHTLIALGNFLFGAI
PDSLGECKSLSRVRLGENYLNGSIPKGLFELPKLTQVELQDNLLTGNFPAVSGAAAPNLGEISLSNNQLTGA
LPASIGNFSGVQKLLLDRNSFSGVVPPE
IGRLQKLSKADLSSNALEGGVPPEIGKCRLLTYLDLSRNNISGKIPPAISGMRILNYLNLSRNHLDGEIPPSIAT
MQSLTAVDFSYNNLSGLVPGTGQFS
YFNATSFVGNPGLCGPYLGPCRPGVAGTDHGGHGHGGLSNGVKLLIVLGLLACSIAFAVGAILKARSLKKA
SEARVWKLTAFQRLDFTCDDVLDCLKEEN
VIGKGGAGIVYKGAMPNGDHVAVKRLPAMGRGSSHDHGFSAEIQTLGRIRHRHIVRLLGFCSNNETNLL
VYEYMPNGSLGELLHGKKGGHLHWDTRYKIA
IEAAKGLCYLHHDCSPLILHRDVKSNNILLDSDFEAHVADFGLAKFLQDTGASECMSAIAGSYGYIAPEYAY
TLKVDEKSDVYSFGVVLLELVTGRKPVG
EFGDGVDIVQWVRMMTDSNKEQVMKVLDPRLSTVPLHEVMHVFYVALLCIEEQSVQRPTMREVVQILS
ELPKLAPRQGEVLSHAVDGFASNPPAPVPSGS
AEALTGDAKDQQQQQTNSESTTPPDLISI*
>LOC_Os04g42700
MGSAAAARTPWALQLGVALAFLLATTCHGLNHEGWLLLTLRKQIVDTFHHLDDWNPEDPSPCGWKGV
NCSSGSTPAVVSLNLSNMNLSGTVDPSIGGLAE
LTNLDLSFNGFSGTIPAEIGNCSKLTGLNLNNNQFQGTIPAELGKLAMMITFNLCNNKLFGAIPDEIGNMA
SLEDLVGYSNNLSGSIPHTIGRLKNLKTV
RLGQNAISGNIPVEIGECLNLVVFGLAQNKLGGPLPKEIGKLTNMTDLILWGNQLSSVIPPEIGNCINLRTIA
LYDNNLVGPIPATIGNIQNLQRLYLYR
NLLNGTIPLEIGNLSLAEEIDFSENVLTGGVPKEFGKIPRLYLLYLFQNQLTGPIPTELCVLRNLSKLDLSINTLS
GPIPACFQYMSRLIQLQLFNNMLS
GDIPPRFGIYSRLWVVDFSNNNITGQIPRDLCRQSNLILLNLGANKLIGNIPHGITSCKSLVQLRLADNSLTG
SFPTDLCNLVNLTTIELGRNKFNGPIP
PQIGNCKSLQRLDLTNNYFTSELPQEIGNLSKLVVFNISSNRLGGSIPLEIFNCTMLQRLDLSQNSFEGSLPN
EVGSLPQLELLSFADNRLSGEIPPILG
KLSHLTALQIGGNQFSGGIPKELGLLSSLQIAMNLSYNNLSGNIPSELGNLALLENLFLNNNKLTGEIPDTFA
NLSSLLEFNVSYNNLTGALPTIPLFDN
MASTSFLGNKGLCGGQLGKCGSESISSSQSSNSGSPPLGKVIAIVAAVIGGISLILIVIIVYHMRKPLETVAPL
QDKQIFSAGSNMQVSTKDAYTFQELV
SATNNFDESCVIGRGACGTVYRAILKAGQTIAVKKLASNREGSNTDNSFRAEILTLGKIRHRNIVKLYGFIYH
QGSNLLLYEYMPRGSLGELLHGQSSSS

LDWETRFMIALGSAEGLSYLHHDCKPRIIHRDIKSNNILLDENFEAHVGDFGLAKVIDMPYSKSMSAIAGS
YGYIAPEYAYTMKVTEKSDIYSYGVVLLE
LLTGRAPVQPLELGGDLVTWVKNYIRDNSLGPGILDKNLNLEDKTSVDHMIEVLKIALLCTSMSPYDRPP
MRNVVVMLSESRDRARMSSSSSPASDHSSK
KDNL*
>LOC_Os06g03970
MAAARAPWLWWWVVVVVGVAVAEAASGGGGGGDGEGKALMGVKAGFGNAANALVDWDGGAD
HCAWRGVTCDNASFAVLALNLSNLNLGGEISPAIGELKN
LQFVDLKGNKLTGQIPDEIGDCISLKYLDLSGNLLYGDIPFSISKLKQLEELILKNNQLTGPIPSTLSQIPNLKTL
DLAQNQLTGDIPRLIYWNEVLQYL
GLRGNSLTGTLSPDMCQLTGLWYFDVRGNNLTGTIPESIGNCTSFEILDISYNQISGEIPYNIGFLQVATLSL
QGNRLTGKIPDVIGLMQALAVLDLSEN
ELVGPIPSILGNLSYTGKLYLHGNKLTGVIPPELGNMSKLSYLQLNDNELVGTIPAELGKLEELFELNLANNN
LQGPIPANISSCTALNKFNVYGNKLNG
SIPAGFQKLESLTYLNLSSNNFKGNIPSELGHIINLDTLDLSYNEFSGPVPATIGDLEHLLELNLSKNHLDGPV
PAEFGNLRSVQVIDMSNNNLSGSLPE
ELGQLQNLDSLILNNNNLVGEIPAQLANCFSLNNLAFQEFVIQQFIWTCPDGKELLEIPNGKHLLISDCNQY
INHKCSFLGNPLLHVYCQDSSCGHSHGQ
RVNISKTAIACIILGFIILLCVLLLAIYKTNQPQPLVKGSDKPVQGPPKLVVLQMDMAIHTYEDIMRLTENLSE
KYIIGYGASSTVYKCELKSGKAIAVK
RLYSQYNHSLREFETELETIGSIRHRNLVSLHGFSLSPHGNLLFYDYMENGSLWDLLHGPSKKVKLNWDTR
LRIAVGAAQGLAYLHHDCNPRIIHRDVKS
SNILLDENFEAHLSDFGIAKCVPSAKSHASTYVLGTIGYIDPEYARTSRLNEKSDVYSFGIVLLELLTGKKAVD
NESNLHQLILSKADDNTVMEAVDSEV
SVTCTDMGLVRKAFQLALLCTKRHPSDRPTMHEVARVLLSLLPASAMTTPKTVDYSRLLASTTTAADMRG
HDVTDIGDNSSSDEQWFVRFGEVISKHTM*
>LOC_Os06g10230
MTPAPAAASYRALVALLLVAVAVADDGSTLLEIKKSFRNVDNVLYDWAGGDYCSWRGVLCDNVTFAVA
ALNLSGLNLGGEISPAVGRLKGIVSIDLKSNG
LSGQIPDEIGDCSSLKTLDLSFNSLDGDIPFSVSKLKHIESLILKNNQLIGVIPSTLSQLPNLKILDLAQNKLSGE
IPRLIYWNEVLQYLGLRGNNLEGS
ISPDICQLTGLWYFDVKNNSLTGPIPETIGNCTSFQVLDLSYNKLSGSIPFNIGFLQVATLSLQGNMFTGPIP
SVIGLMQALAVLDLSYNQLSGPIPSIL
GNLTYTEKLYMQGNKLTGPIPPELGNMSTLHYLELNDNQLSGFIPPEFGKLTGLFDLNLANNNFEGPIPDN
ISSCVNLNSFNAYGNRLNGTIPPSLHKLE
SMTYLNLSSNFLSGSIPIELSRINNLDTLDLSCNMITGPIPSTIGSLEHLLRLNLSNNGLVGFIPAEIGNLRSIM
EIDMSNNHLGGLIPQELGMLQNLML
LNLKNNNITGDVSSLMNCFSLNILNVSYNNLAGVVPTDNNFSRFSPDSFLGNPGLCGYWLGSSCRSSGH
QQKPLISKAAILGIAVGGLVILLMILVAVCR
PHSPPVFKDVSVSKPVSNVPPKLVILHMNLSLLVYEDIMTMTENLSEKYIIGYGASSTVYKCVSKNRKPVAV
KKLYAHYPQSFKEFETELETVGSIKHRN
LVSLQGYSLSPVGNLLFYDYMENGSLWDVLHEGPTKKKKLDWETRLRIALGAAQGLAYLHHDCSPRIIHR
DVKSKNILLDKDYEAHLTDFGIAKSLCVSK
THTSTYVMGTIGYIDPEYARTSRLNEKSDVYSYGIVLLELLTGKKPVDNECNLHHLILSKTANNAVMETVDP
DIADTCKDLGEVKKVFQLALLCTKRQPS
DRPTMHEVVRVLDCLVRPDPPPKSAQQLAMPQRPAVPSYINEYVSLRGTSVLSCANSSCTSDAELFLKFG
EVISQNTE*

>LOC_Os06g38990
MAHRGDRFLVVVVVVVVLGVVVRPAAALSADGKALLSLLPAAAPSPVLPSWDPTAATPCSWQGVTCSP
QSRVVSLSLPNTFLNLSSLPPQLASLSSLQLL
NLSTCNISGAIPPAYASLAALRVLDLSSNALYGDIPASLGALSGLQYLLLNSNRLTGAIPRSLASLAALQVLCV
QDNLLNGTIPASLGALTALQQFRVGG
NPGLSGPIPASLGALSNLTVFGAAATALSGAIPEELGNLANLQTLALYDTGVSGPIPAALGGCAELRNLYLH
MNKLTGPIPPELGRLQKLTSLLLWGNAL
SGRIPPELSNCSALVVLDLSGNRLAGEVPGALGRLAALEQLHLSDNQLAGRIPAELSNCSSLTALQLDKNGL
TGAIPPQLGELRALQVLFLWGNALSGAI
PPSLGNCTELYALDLSRNRLAGGIPDEVFALQKLSKLLLLGNALSGRLPPSVADCSSLVRLRLGENQLAGEIP
REIGKLPNLVFLDLYSNKFTGALPGEL
ANITVLELLDVHNNSFTGAIPPQFGELMNLEQLDLSMNKLTGEIPASFGNFSYLNKLILSGNMLSGTLPKSI
RNLQKLTMLELSNNSFSGPIPPEIGALS
SLSISLDLSSNRFTGELPDEMSSLTQLQSLDLSSNGLYGSISVLSGLTSLTSLNISYNNFSGAIPVTPFFKTLSSS
SYINNPNLCESYDGHTCASDMVRR
TALKTVKTVILVCAVLGSITLLLVVVWILINRSRTLAGKKAMSMSVAGGDDFSHPWTFTPFQKLNFCVDNI
LECLRDENVIGKGCSGVVYRAEMPNGEII
AVKKLWKTSKEEPIDAFAAEIQILGHIRHRNIVKLLGYCSNKYVKLLLYNYIPNGNLQQLLKDNRSLDWDTR
YKIAVGAAQGLAYLHHDCVPAILHRDVK
CNNILLDTKYEAYLADFGLAKLMNSPNYHHAMSRIAGSYGYIAPEYGYTTKITEKSDVYSYGVVLLEILSGRS
AVEAVVGDSLHIVEWAKKKMGSYEPAV
NILDPKLRGMPDQLVQEMLQTLGIAIFCVNPAPAERPTMKEVVAFLKEVKCSPEEWGKISQQPLIKPGSQ
QG*
>LOC_Os07g04190
MRLHYHHLAVVLLAAVAAAATAAAGGEADALLAVKAALDDPTGALASWTTNTTSSPCAWSGVACNAR
GAVVGLDVSGRNLTGGLPGAALSGLQHLARLDL
AANALSGPIPAALSRLAPFLTHLNLSNNGLNGTFPPQLSRLRALRVLDLYNNNLTGALPLEVVSMAQLRHL
HLGGNFFSGGIPPEYGRWGRLQYLAVSGN
ELSGKIPPELGNLTSLRELYIGYFNSYSGGIPPELGNMTDLVRLDAANCGLSGEIPPELGNLANLDTLFLQVN
GLAGGIPRELGKLASLSSLDLSNNALA
GEIPATFADLKNLTLLNLFRNKLRGDIPEFVGDLPSLEVLQLWENNFTGGIPRRLGRNGRFQLLDLSSNRLT
GTLPPDLCAGGKLETLIALGNSLFGAIP
ASLGKCTSLTRVRLGDNYLNGSIPEGLFELPNLTQVELQDNLISGGFPAVSGTGAPNLGQISLSNNQLTGAL
PAFIGSFSGVQKLLLDQNAFTGEIPPEI
GRLQQLSKADLSGNSFDGGVPPEIGKCRLLTYLDLSRNNLSGEIPPAISGMRILNYLNLSRNQLDGEIPATIA
AMQSLTAVDFSYNNLSGLVPATGQFSY
FNATSFVGNPGLCGPYLGPCHPGAPGTDHGGRSHGGLSNSFKLLIVLGLLALSIAFAAMAILKARSLKKASE
ARAWKLTAFQRLEFTCDDVLDSLKEENI
IGKGGAGTVYKGTMPDGEHVAVKRLPAMSRGSSHDHGFSAEIQTLGRIRHRYIVRLLGFCSNNETNLLVY
EYMPNGSLGELLHGKKGGHLHWDTRYKVAV
EAAKGLCYLHHDCSPPILHRDVKSNNILLDSDFEAHVADFGLAKFLQDSGTSECMSAIAGSYGYIAPEYAYT
LKVDEKSDVYSFGVVLLELITGKKPVGE
FGDGVDIVQWVKTMTDSNKEHVIKILDPRLSTVPVHEVMHVFYVALLCVEEQSVQRPTMREVVQILSEL
PKPTSKQGEEPPSGEGAVFDLVVPAESAEAN
EAKEQQQQQLNSPSSPPPDLISI*
>LOC_Os07g05740

MAASVARVLLAAAVFFAAVAAAAAASSSAAAVAALMEFKTKLDDVDGRLSSWDAAGGSGGGDPCGW
PGIACSAAMEVTAVTLHGLNLHGELSAAVCALPR
LAVLNVSKNALAGALPPGLAACRALEVLDLSTNSLHGGIPPSLCSLPSLRQLFLSENFLSGEIPAAIGNLTALE
ELEIYSNNLTGGIPTTIAALQRLRII
RAGLNDLSGPIPVEISACASLAVLGLAQNNLAGELPGELSRLKNLTTLILWQNALSGEIPPELGDIPSLEMLA
LNDNAFTGGVPRELGALPSLAKLYIYR
NQLDGTIPRELGDLQSAVEIDLSENKLTGVIPGELGRIPTLRLLYLFENRLQGSIPPELGELTVIRRIDLSINNLT
GTIPMEFQNLTDLEYLQLFDNQIH
GVIPPMLGAGSNLSVLDLSDNRLTGSIPPHLCKFQKLIFLSLGSNRLIGNIPPGVKACRTLTQLQLGGNMLT
GSLPVELSLLRNLSSLDMNRNRFSGPIP
PEIGKFRSIERLILSENYFVGQIPPGIGNLTKLVAFNISSNQLTGPIPRELARCTKLQRLDLSKNSLTGVIPQEL
GTLVNLEQLKLSDNSLNGTVPSSFG
GLSRLTELQMGGNRLSGQLPVELGQLTALQIALNVSYNMLSGEIPTQLGNLHMLEFLYLNNNELEGEVPS
SFGELSSLLECNLSYNNLAGPLPSTTLFQH
MDSSNFLGNNGLCGIKGKSCSGLSGSAYASREAAVQKKRLLREKIISISSIVIAFVSLVLIAVVCWSLKSKIPD
LVSNEERKTGFSGPHYFLKERITFQE
LMKVTDSFSESAVIGRGACGTVYKAIMPDGRRVAVKKLKCQGEGSNVDRSFRAEITTLGNVRHRNIVKLY
GFCSNQDCNLILYEYMANGSLGELLHGSKD
VCLLDWDTRYRIALGAAEGLRYLHSDCKPKVIHRDIKSNNILLDEMMEAHVGDFGLAKLIDISNSRTMSAI
AGSYGYIAPEYAFTMKVTEKCDIYSFGVV
LLELVTGQSPIQPLEQGGDLVNLVRRMTNSSTTNSEIFDSRLNLNSRRVLEEISLVLKIALFCTSESPLDRPS
MREVISMLMDARASAYDSFSSPASEAP
IEDDSSLKH*
>LOC_Os08g38560
MPPRWRATATRLLVLLACACAVFVPRCHCVGDQGEALLRWKASLLNGTGGGGGGLDSWRASDASPCR
WLGVSCDARGDVVAVTIKTVDLGGALPAASVLP
LARSLKTLVLSGTNLTGAIPKELGDLAELSTLDLTKNQLTGAIPAELCRLRKLQSLALNSNSLRGAIPDAIGNL
TGLTSLTLYDNELSGAIPASIGNLKK
LQVLRAGGNQALKGPLPPEIGGCTDLTMLGLAETGISGSLPATIGNLKKIQTIAIYTAMLTGSIPESIGNCTE
LTSLYLYQNTLSGGIPPQLGQLKKLQT
VLLWQNQLVGTIPPEIGNCKELVLIDLSLNELTGPIPRSFGGLPNLQQLQLSTNKLTGVIPPELSNCTSLTDIE
VDNNQLTGAIGVDFPRLRNLTLFYAW
QNRLTGGIPASLAQCEGLQSLDLSYNNLTGAIPRELFALQNLTKLLLLSNDLAGFIPPEIGNCTNLYRLRLNG
NRLSGTIPAEIGNLKNLNFLDLGGNRL
TGPLPAAMSGCDNLEFMDLHSNALTGTLPGDLPRSLQFVDVSDNRLTGVLGAGIGSLPELTKLNLGKNRI
SGGIPPELGSCEKLQLLDLGDNALSGGIPP
ELGKLPFLEISLNLSCNRLSGEIPSQFAGLDKLGCLDVSYNQLSGSLEPLARLENLVTLNISYNAFSGELPDTA
FFQKLPINDIAGNHLLVVGSGGDEAT
RRAAISSLKLAMTVLAVVSALLLLSATYVLARSRRSDSSGAIHGAGEAWEVTLYQKLDFSVDEVVRSLTSAN
VIGTGSSGVVYRVGLPSGDSVAVKKMWS
SDEAGAFRNEIAALGSIRHRNIVRLLGWGANRSTKLLFYTYLPNGSLSGFLHRGGVKGAAEWAPRYDIALG
VAHAVAYLHHDCLPAILHGDIKAMNVLLG
PRNEPYLADFGLARVLSGAVDSGSAKVDSSKPRIAGSYGYIAPEYASMQRISEKSDVYSFGVVVLEILTGRH
PLDPTLPGGTHLVQWVRDHLQAKRAVAE
LLDPRLRGKPEAQVQEMLQVFSVAVLCIAHRADDRPAMKDVVALLKEIRRPVEGGATGGGEGKEQNAA
AAAAAPPAAERRSPARSTLPKGGSSSCSFAMS
DYSS*

>LOC_Os10g06740
MSRHLLLLLALLLLLLLFPLATSAQPPPPSNTSASTSAAAVLLSFLDSLPPASQRLLLPSWRQSRSSSSSGNAT
APPPHCAFLGVTCSDTGAVAALNLSG
VGLTGALSASAPRLCALPASALPVLDLSGNGFTGAVPAALAACAGVATLLLGGNNLSGGVPPELLSSRQLV
EVDLNGNALTGEIPAPAGSPVVLEYLDLS
GNSLSGAVPPELAALPDLRYLDLSINRLTGPMPEFPVHCRLKFLGLYRNQIAGELPKSLGNCGNLTVLFLSY
NNLTGEVPDFFASMPNLQKLYLDDNHFA
GELPASIGELVSLEKLVVTANRFTGTIPETIGNCRCLIMLYLNSNNFTGSIPAFIGNLSRLEMFSMAENGITG
SIPPEIGKCRQLVDLQLHKNSLTGTIP
PEIGELSRLQKLYLYNNLLHGPVPQALWRLVDMVELFLNDNRLSGEVHEDITQMSNLREITLYNNNFTGEL
PQALGMNTTSGLLRVDFTRNRFRGAIPPG
LCTRGQLAVLDLGNNQFDGGFSSGIAKCESLYRVNLNNNKLSGSLPADLSTNRGVTHLDISGNLLKGRIPG
ALGLWHNLTRLDVSGNKFSGPIPHELGAL
SILDTLLMSSNRLTGAIPHELGNCKRLAHLDLGNNLLNGSIPAEITTLSGLQNLLLGGNKLAGPIPDSFTATQ
SLLELQLGSNNLEGGIPQSVGNLQYIS
QGLNISNNRLSGPIPHSLGNLQKLEVLDLSNNSLSGPIPSQLSNMISLSVVNISFNELSGQLPDGWDKIATR
LPQGFLGNPQLCVPSGNAPCTKYQSAKN
KRRNTQIIVALLVSTLALMIASLVIIHFIVKRSQRLSANRVSMRNLDSTEELPEDLTYEDILRATDNWSEKYVI
GRGRHGTVYRTELAVGKQWAVKTVDL
SQCKFPIEMKILNTVKHRNIVRMAGYCIRSNIGLILYEYMPEGTLFELLHERTPQVSLDWNVRHQIALGVA
ESLSYLHHDCVPMIIHRDVKSSNILMDAE
LVPKLTDFGMGKIIDDDDADATVSVVVGTLGYIAPEHGYSTRLSEKSDVYSYGVVLLELLCRKMPVDPAFG
DGVDIVTWMGSNLNQADHSNIMRFLDEEI
IYWPEHEKAKVLDLLDLAMTCTQVSCQLRPSMREVVSILMRIERSNHVQFFEEAP*
>LOC_Os10g06760
MSHAPRFLLFLLQLLAASVVARPPPPERADSAAVLRSFLASLPPPSRRVLRPSWRRGGGGGAPHCAFLGV
TCDAAGAVAALNLSGAGLAGELAASAPRLC
ALPALAALDLSRNGFTGSVPAALAACSCIATLVLSFNSLSGAVPPEILSSRRLRKVDLNSNALTGEIPTTGLA
AGSSVLEYLDLCVNSLSGAIPPELAAA
LPELTYLDLSSNNLSGPMPEFPPRCGLVYLSLYSNQLAGELPRSLTNCGNLTVLYLSYNKIGGEVPDFFASM
ANLQTLYLDDNAFVGELPASIGELVNLE
ELVVSENAFTGTIPEAIGRCRSLTMLYLNGNRFTGSIPKFIGDLTRLQLFSIADNGITGEIPPEIGKCRGLVEIA
LQNNSLSGMIPPDIAELNQLQKLSL
FDNILRGPVPLALWRLSNMAVLQLNNNSFSGEIHSDITQMRNLTNITLYNNNFTGELPQELGLNTTPGLL
HIDLTRNHFRGAIPPGLCTGGQLAVLDLGY
NQFDGGFPSEIAKCQSLYRVNLNNNQINGSLPADFGTNWGLSYIDMSSNLLEGIIPSALGSWSNLTKLDLS
SNSFSGPIPRELGNLSNLGTLRMSSNRLT
GPIPHELGNCKKLALLDLGNNFLSGSIPAEITTLGSLQNLLLAGNNLTGTIPDSFTATQALLELQLGDNSLEG
AIPHSLGSLQYISKALNISNNQLSGQI
PSSLGNLQDLEVLDLSNNSLSGIIPSQLINMISLSVVNLSFNKLSGELPAGWAKLAAQSPESFLGNPQLCVH
SSDAPCLKSQSAKNRTWKTRIVVGLVIS
SFSVMVASLFAIRYILKRSQRLSTNRVSVRNMDSTEELPEELTYEDILRGTDNWSEKYVIGRGRHGTVYRTE
CKLGKQWAVKTVDLSQCKLPIEMKILNT
VKHRNIVRMAGYCIRGSVGLILYEYMPEGTLFELLHRRKPHAALDWTVRHQIAFGVAQGLSYLHHDCVP
MIVHRDVKSSNILMDTELVPKLTDFGMGKIV
EDDDLDATVSVVVGTLGYIAPEHGYYTRLTEKSDVYSYGVVLLELLCRKMPVDPAFGDSVDIVTWMRSNL
TQADRRVIMECLDEEIMYWPEDEQAKALDL

LDLAMYCTQLACQSRPSMREVVNNLMRMDK*
>LOC_Os10g33040
MPRRHDALRLLLLVVVVAAADAATEADALLAWKAGLQDGAAALSGWSRAAPVCAWRGVACDAAAGG
ARVTSLRLRGAGLGGGLDALDFAALPALAELDLN
GNNFTGAIPASISRLRSLASLDLGNNGFSDSIPPQLGDLSGLVDLRLYNNNLVGAIPHQLSRLPKVAHFDLG
ANYLTDEDFAKFSPMPTVTFMSLYLNSF
NGSFPEFILKSGNVTYLDLSQNTLFGKIPDTLPEKLPNLRYLNLSINAFSGPIPASLGKLTKLQDLRMAANNL
TGGVPEFLGSMPQLRILELGDNQLGGP
IPPVLGQLQMLQRLDIKNSGLSSTLPSQLGNLKNLIFFELSLNQLSGGLPPEFAGMRAMRYFGISTNNLTG
EIPPVLFTSWPELISFQVQNNSLTGKIPP
ELGKASKLNILYLFTNKFTGSIPAELGELENLTELDLSVNSLTGPIPSSFGNLKQLTKLALFFNNLTGVIPPEIG
NMTALQSLDVNTNSLHGELPATITA
LRSLQYLAVFDNHMSGTIPADLGKGLALQHVSFTNNSFSGELPRHICDGFALDHLTANYNNFTGALPPCL
KNCTALVRVRLEENHFTGDISEAFGVHPKL
VYLDVSGNKLTGELSSAWGQCINLTLLHLDGNRISGGIPAAFGSMTSLKDLNLAGNNLTGGIPPVLGNIRV
FNLNLSHNSFSGPIPASLSNNSKLQKVDF
SGNMLDGTIPVAISKLDALILLDLSKNRLSGEIPSELGNLAQLQILLDLSSNSLSGAIPPNLEKLITLQRLNLSH
NELSGSIPAGFSRMSSLESVDFSYN
RLTGSIPSGNVFQNASASAYVGNSGLCGDVQGLTPCDISSTGSSSGHHKRVVIATVVSVVGVVLLLAVVTC
IILLCRRRPREKKEVESNTNYSYESTIWE
KEGKFTFFDIVNATDNFNETFCIGKGGFGSVYRAELSSGQVVAVKRFHVADTGDIPDVNKKSFENEIKALT
EVRHRNIVKLHGFCTSGDYMYLVYEYLER
GSLGKTLYGEEGKKKMDWGMRVKVVQGLAHALAYLHHDCNPAIVHRDITVNNILLESDFEPRLCDFGTA
KLLGGASTNWTSVAGSYGYMAPEFAYTMRVT
EKCDVYSFGVVALEVMMGKHPGDLLTSLPAISSSEEDDLLLKDILDQRLDAPTGQLAEEVVFIVRIALGCTR
VNPESRPSMRSVAQEISAHTQAYLSEPF
KLITISKLTDYQK*
>Sevir.1G050100
MAPKRHAASRRLSPLDGPPSPPSLRDRLVSPHHCLPAFIFMCPPGGVKVKLGVEMERKRGLSSATSRAGA
PGEPITSQIWSRSAFAGSSDIENLYDLRDA
VAKSKDSLSDWFGTETCPCNWRGITCEGDTVVAINLSSVRLHIPFPLCITAFRSLGMLNLSGCDLSGQIPEA
LGNLQQLQYLDLSSNQLAGPIPFSLYDL
KTLKEIVLDRNSVSGQLSPAIGQLQNLTKLSISRNNISGELPPELGSLKNLEVLDLQLNRFNGSIPEAFGNLTR
LFYLDASRNKLTGSIFPGISALLNLL
TIDFSSNSLVGPIPNEITHLKMLERLALGFNHFTGGIPKEIGELPGKLFESSTFLELSLDNNNLTGHIPESIGKL
HSLQRLRMGSNHLEGPIPLAVGALE
NLTEISLDGNRLSGSIPQELFNCRNLVKLNLSSNSLMGPIPRSISQLTSVTGLVLSHNQLSGSIPAEICGGFTN
PTHPESEYVQHHGFLDLSYNLLSGRI
PPAIKNCVILEELLLQGNLLNGSIPAEVAELKNITKIDLSFNALVGPMLPWSAPLLKLQGLFLSNNHLSGNIP
AEIGRILPNIAVLNLSGICAADSINRR
GSHTPHVILTVVAICVAVTVVIVVLLVFFLRWKLLRNNRSLPLVPTTASQSSATTEPSSMEPPSINLATFEHA
LLRFTLEDILKATNNFSNVHIIGQGGF
GTVYKAALPEGRRVAIKRLYGSHQFLGDRQFLAEMETIGKVKHRNLVPLLGYCARGDERFLIYEHMSHGSL
ETWLRDRANAPKAIGWPDRLRICIGSAHG
LMFLHHGFVPRIIHRDMKSSNILLDENMEPRISDFGLARIISAYDTHVSTNVAGTLGYIPPEYAMTMKCTA
KGDVFSFGVVMLEVLTGRPPTGQEVEEGG

GNIIDWVRWMIAQGREGELFDPSLPVSGLWREQMVRVLAIALDCTADEPRNRPTMPDVVKGLKIAELM
ESEPHDLPGRVAQP*
>Sevir.1G114100
MEARAPVLVLAVTLSLILATGVGAAAGDERAALLALKAGFVDSLGALADWKGSSHCSWTAVGCNAAGL
VDRLNLSGKNLSGKVTDDVLRLPSLTVLNLSS
NAFAVALPKSFAALSKLQVFDVSQNSFDGAFPAGLSSCADLAIVNASGNNFVGALPADLANATSLETIDLR
GSFFGGDIPAAYRSLIKLKFLGLSGNNIT
GKIPPELGELESLESLIIGYNALEGGIPPELGNLASLQYLDLAVGSLDGPIPAALGRLPALTSLYLYKNNLEGKI
PPELGNISTLVFLDLSDNLLTGPIP
DEVSQLSHLRLLNLMCNHLDGTVPAAIGDMPSLEVFELWNNSLTGQLPASLGKSSPLQWVDVSSNSFSG
PVPAGICDGKSLAKLIMFNNGFTGGIPAGLA
SCASLVRVRMQSNRLTGTIPIGFGKLPSLQRLELAGNDLSGELPGDLALSTSLSFIDVSHNHLQYSLPSSLFTI
PTLQSFLASDNIISGELPDQFQDCPA
LAALDLSNNRLAGTIPSSLASCQRLVKLNLRHNRLTGEIPKALAKMPAMAILDLSSNSLTGGIPENFGSSPA
LETLNLAYNNLTGPVPGNGVLRSINPDE
LAGNAGLCGGVLPPCSGSRDMGLAAARPHGSARLKRIAVGWLAGMLAVVAVFAAALGGRYAYRRWY
MGGGGCCGDDESLGAESGAWPWRLTAFQRLGFTS
ADVLACVKEANVVGMGATGVVYKAELPRARTVIAVKKLWRPAAIDGDAAAGNELTADVLKEVGLLGRLR
HRNIVRLLGYLHNDSDAMMLYEFMPNGSLWE
ALHGPPEKRALADWVSRYDVXGVAQGLAYLHHDCHPPVIHRDIKSNNILLDANMEARIADFGLARALAR
TNESVSVVAGSYGYIAPEYGYTLKVDQKSDI
YSYGVVLMELITGRRAVEAEFGEGQDIVGWVREKIRSNTVEEHLDANVGGRCAHVREEMLLVLRIAVLCT
ARAPRDRPSMRDVITMIGEAKPRRKSGSSG
AGKDAGAAVPAAVVVDKDKPVFSTTPDSDYA*
>Sevir.1G345500
MTRLLRALAAFLLLAAVAVADDGATLLEIKKSFSNGGNALHDWSGEGASPTYCSWRGVLCDNVTFAVAA
LNLSGLNLEGEISPAIGSLKRVISIDLKSNG
LSGQIPDEIGDCSLLETLDLSSNNLEGDIPFSISKLKHLENLILKNNQLVGVIPSTLSQLPNLKILDLAQNKLSG
EIPNLIYWNEVLQYLGLRSNNLEGS
LSPDMCQLTGLWYFDVKNNSLMGMIPETIGNCTSFQVLDLSNNQLTGEIPFNIGFLQVATLSLQGNKFSG
PIPSVIGLMQALAVLDLSFNELSGPIPSIL
GNLTYTEKLYLQGNRLTGSIPPELGNMTTLHYLELNDNLLTGFIPPDLGKLTELFDLNLANNNLGGPIPDNIS
SCINLISLNAYGNKLNGTIPRSFQKLE
SLTYLNLSSNHLSGALPIEVARMRNLDTLDLSCNMITGSIPSAIGRLEHLLRLNLSKNALVGHIPAEFGNLRSI
MEIDLSSNYLRGLIPQEVGMLQNLIL
LKLENNSITGDVSPLTNCFSLNNLNVSYNDLAGIVPTDNNFSRFSPDSFLGNPGLCGYWRGSRSSCSPLSSS
IERKRRSSISKAAFLGIGVGGLVILLVI
LAAACWPHNSPVLKDVSVSKPDNLAAASSSVPPKLVILHMNMALYVYDDIMRMTENLSEKYIIGYGASST
VYRCDLKNCKSVAIKKLYTHYPQSLKEFET
ELETVGSIKHRNLVSLQGYSLSPAGNLLFYDYMENGSLWDVLHVASSKKEKLDWEARLKIALGAAQGLAY
LHHECSPRIIHRDVKSKNILLDKDYEAHLA
DFGIAKSLCVSLTHTSTYVMGTIGYIDPEYARTSRLNEKSDVYSYGIVLLELLTGKKPVDDECNLHHLILSKAA
DNTVMEMVDPDITDTCKDLGEVKKVF
QLALLCSKRQPSDRPTMHEVVRVLDSLVCPDPPPKQAQPQGSEQSATAPSYVSEYVSLRGGTALSCANSS
SASDAELFMKFGEVISRNTE*
>Sevir.2G031700

MAMPMRLHYLLLPILLAAAAAAAAAATVNADADALLAAKAELADPAGALASWTANATASPCAWSGVT
CNARGAVIGVDLSGRNLSGPVPAALSRLPHLAR
LDLAANAFSGPIPTPLARLRYLTHLNLSNNVLNGTFPPPLARLRTLRVVDLYNNNLTGPLPLGVAALPALRH
LHLGGNFFSGEIPPEYGTWGRLQYLAVS
GNELSGRIPPELGNLTSLRELYIGYYNSYSGGIPPELGNMTELVRLDAANCGLSGEIPPELGNLANLDTLFLQ
VNGLAGGIPPELGRLRSLSSLDLSNNA
LTGEIPATFAALKNLTLLNLFRNKLRGSIPELVGDLPSLEVLQLWENNFTGGIPRRLGRNGRLQLVDLSSNRL
TGTLPPDLCAGGKLETLIALGNFLFGS
IPESLGKCEALSRIRLGENYLNGSIPEGLFELPNLVQVELQDNLLSGGFPAVAGAAASNLGSITLSNNQLTG
ALPASIGNFSGLQKLLLDQNAFNGAVPP
EIGRLQQLSKADLSGNSLDGGVPPEIGKCRLLTYLDLSRNNLSGEIPPAISGMRILNYLNLSRNHLDGEIPATI
AAMQSLTAVDFSYNNLSGLVPATGQF
SYFNATSFVGNPGLCGPYLGPCHSGGAGTDHGARSHGGISNTFKLLIVLGLLVCSIAFAAMAILKARSLKKA
SEARAWRLTAFQRLDFTCDDVLDSLKEE
NIIGKGGAGIVYKGTMPDGEHVAVKRLSAMSRGSSHDHGFSAEIQTLGRIRHRYIVRLLGFCSNNETNLLV
YEYMPNGSLGELLHGKKGGHLHWDTRYKI
AVEAAKGLSYLHHDCSPPILHRDVKSNNILLDSDFEAHVADFGLAKFLQDSGASQCMSAIAGSYGYIAPEY
AYTLKVDEKSDVYSFGVVLLELVTGKKPV
GEFGDGVDIVQWVKTMTDSNKEQVIKIMDPRLSTVPVHEVMHIFYVALLCVEEQSVQRPTMREVVQM
LSELPKPTSRQGDELPSGDDGAAPNPPVSADSV
EALNDEAKEHQQQKQPSSSQSSPTRDLISI*
>Sevir.2G037800
MASPVARFSLLPAVVLFLLAVLEPAGAGPEAAALLEFKRALVDVDGRLSGWNAAAAAGACEWAGIACSA
GGEVTGVTLHGLNLHGELSAAVCALPRLAVL
NVSKNALGGAIPPGLAACAALEVLDLSTNALRGSVPPELCALRGLRRLFLSENLLSGEIPPAVGGLAALEELE
IYSNNLTGRIPASIRALRRLRVIRAGL
NDISGPIPVELTECASLEVLGLAQNNLAGELPRELSRLKNLTTLILWQNAFSGEVPPELGNCTNLQMLALN
DNAFTGGVPRELAALPSLLKLYIYRNQLD
GTIPPELGNLQSVLEIDLSENKLTGIIPGELGRIPTLRLLYLFENRLQGSIPPELGQLSSIRKIDLSINNLTGVIPT
AFQNLSSLEYLELFDNQLHGDIP
PLLGANSNLSVLDLSDNQLTGSIPPHLCKYQKLMFLSLGSNRLVGNIPPGLKACRTLTQLRLGGNMLTGSL
PVELSLLQNLTSLEMNQNRFSGPIPPEIG
KFRSIERLILSNNHFVGQLPAAIGNLTELVAFNISLNQLSGPIPRELAQCKKLQRLDLSRNSLTGAIPQEIGGL
VNLELLKLSDNSLNGSIPSSFGGLSR
LIALEMGGNRLSGQVPVELGELTALQIALNVSHNMLSGEIPMQLGNLHMLQYLYLDNNELEGRVPSSFSE
LSSLLECNLSYNNLFGPLPSTPLFEHLDSS
NFLGNNGLCGIKGKACPGSSASSYSSKEAAAQKKRFLREKIISIASIVIALVSLVLIAVVCWAFRSKIPELVSSD
ERKTGFSGPHYCMKERVTYQELMKA
TEDFSESAVIGRGACGTVYKAVMPDGRKIAVKRLKSQGEGSNIDKSFRAEITTLGNVRHRNIVKLYGFCSH
QDSNLILYEYMENGSLGELLHGSKDAYLL
DWDTRYRIALGAAEGLRYLHSDCKPQVIHRDIKSNNILLDAMMEAHVGDFGLAKLIDISNSRTMSAVAGS
YGYIAPEYAFTMKVTEKCDVYSFGVVLLEL
LTGQSPIQPLEKGGDLVNLVRRMMNKMTPSREMFDSRLDLSSRRVVEEMSLVLKIALFCTNESPFDRPS
MREVISMLMDARASSYDSFSSPASEAPIEDG
SSPKV*
>Sevir.2G331800

MAAGRWFLASAWFLVVLALLSCFAAADDGDVLLEVKRAFVGDLEGVLAGWNASGAGAGAAGFCSWA
GVACDDAGLRVVSLNLSGAGLAGPVPRALARLDA
LQAIDLSSNALAGPIPAALGALASLQVLLLYSNQLTGEIPASLGKLAVLQVLRAGDNPGLSGAIPDALGELG
NLTVLGLASCNLTGPIPAGLGRLAALTA
LNLQQNALSGPIPRGLAGLASLQVLALAGNQLTGAIPPELGRLTGLQKLNLGNNSLVGAIPPELGELGELQ
YLNLMNNRLSGRVPRALAKLSSVRMIDLS
GNMLSGDLPAELGRLPELTFLVLSDNQLTGSVPGDLCGGAGASDEAESSSLEHLMLSTNNFTGEIPEGLSR
CQALTQLDLANNSLSGAIPAALGELGNLT
DLMLNNNSLSGELPPELFNLTELQTLALYHNKLTGRLPDAIGRLVNLEVLYLYENQFAGEIPESIGDCTSLQ
MIDIFGNRFNGSIPASMGNLSQLAFIDF
RQNELSGSIPPELGECRQLQVLDLADNALSGPIPETFGKLRSLQQFMLYNNSLSGTIPDSMFECRNITRVNI
AHNRLTGSLLPLCGTARLLSFDATNNSF
HGGIPAQLGRSSSLQRVRLGSNMLSGPIPPSLGGIAALTLLDVSNNALTGSIPATLAQCKQLSLIVLSHNRLS
GPVPDWLGSLPQLGELTLSNNEFAGPL
PVQLSNCSKLLKLSLDNNQINGTVPPEIGSLVSLNVLNLAHNQLSGLIPTTIAKLNNLYELNLSQNFLSGPIPP
DIGKLQELQSLLDLSSNNFSGHIPAS
LGSLSKLEDLNLSHNALVGAVPSQLAGMSSLVQLDLSSNQLEGRLGAEFGRWPQGAFADNVGLCGSPLR
ACSSGGGPSTLSSVTIALVSAAVTLSVVLLI
IVLALMVVRRRGRRSREVNCTAFSSSSANTNRQLVVKGSARREFRWEAIMEATANLSDQFAIGSGGSGT
VYRAELSTGETVAVKRIAHMDSDMLLHDKSF
TREIKILGRVRHRHLVKLLGFITSHDAGAGGSMLVYEYMENGSLYDWLHGGVGGDGSRKKRVLGWDAR
LKVAAGLAQGVEYLHHDCVPRIVHRDIKSSNV
LLDGDMEAHLGDFGLAKAVAENRQAAFGKDCTESASCFAGSYGYIAPECAYSLKATERSDVYSMGIVLM
ELVTGLLPTDKTFGGDMDMVRWVQSRMGAPL
PAREQVFDPALKPLAPREESSMAEVLEVALRCTRTASGERPTARQVSDLLLHVSLDYYRAGEKR*
>Sevir.3G021300
MGAFRWLFHFLLAAVLLHVHGGQSLNQTCHPTDRQALLNFSNGLDSKAAGLVGWGPDDDACCSWTG
VACDLGRVVGLDLSNKSLHGGISSSVASLDGLVT
LNLSRNSLRGAAPVALGQLARLRVLDLSANGLSGTFPASDGGFPAIEVVNISSNTFDGPHPAFPAAANLTV
LDISGNNFSGGINSSALCIAPVEVLRFSG
NGFSGEVPSGLSRCKALAELSLDGNCLTGNIPGDLYTLPKLTRLSLQENKLTGNLGNDLGNLSQLVQLDLSY
NRFSGSIPDVFGGMRRLECLNLASNMFH
GELPASLSRCPTLRVISLRNNSLSGEIAIDFKFLPKLNTFDVGSNNLIGAIPSGISSCPELRTLNLARNKLVGEIP
ETFKDLRSVSYLSLTGNGFTNLSS
ALQVLQHLPNLTSLVLTRNFRGGETMPVDGINGFKSMEVLVLANCLLTGTIPPWLQTLESLNVLDISWNK
LNGNIPPWLGKLNNLFYIDLSNNSFSGELP
VSFTQMRSLISSNGSSEQSPTEDLPLFIKKNSTGKGLQYNQVSSFPPSLILSNNLLIGPIWSSFGHLVKLQHM
DLSWNKFSGPIPDELSNMSSLEVLNLA
HNNLNGTIPSSLTKLNFLSKFDVSYNNLTGDVPTGGQFSTFTNEDFEGNSALCLLRNSSCSEKASLVEAAR
GKKSKGALVGLGLGTAVGVAAFLFCAYVI
VARIVHSRMQECNPKAVANAEDSESSNSCLVLLFQNNKEFSIEDILKSTNNFDQAYIVGCGGFGLVYKSTL
PDGRRVAIKRLSGDYSQIEREFQAEVETL
SRAQHENLVLLQGYCKVGNDRLLIYSYMENGSLDYWLHERADSGMLLDWRKRLRIAQGAARGLAYLHM
SCDPHILHRDIKSSNILLDENFEAHLADFGLA
RLICAYETHVTTDVVGTLGYIPPEYGQSPVATYKGDIYSFGIVLLELLTGRRPVDMCRPKGTRDVVSWVLQ
MKEEGRETEVFHPSIHHKENESQLMRVLE
IACLCVTAAPKSRPTSQQLVAWLDNIAEDGGLMQPEVSSGFDLLA*

>Sevir.4G019500
MAALRPWWSAAAAVLVLAAAAAGGGDGDGERRALMAVKAGFGNAANALVDWDGGRDHCAWRGV
ACDSASFAVVGLNLSNLNLGGEISPAIGELKSLQFVD
LKLNKLTGQIPDEIGDCVSLKYLDLSGNLLYGDIPFSISKLKQLEDLILKNNQLTGPIPSTLSQIPNLKTLDLAQ
NKLTGDIPRLIYWNEVLQYLGLRGN
SLTGTLSPDMCQLTGLWYFDVRGNNLTGTIPEGIGNCTSFEILDISYNQISGEIPYNIGYLQVATLSLQGNRL
TGRIPEVIGLMQALAVLDLSENDLVGP
IPPILGNLSYTGKLYLHGNKLTGHIPPELGNMSKLSYLQLNDNELTGTIPAELGKLTELFELNLANNNLEGHI
PANISSCSALNKFNVYGNRLNGSIPGG
FQKLESLTYLNLSSNNLKGQIPSELGHIVNLDTLDLSYNDFSGPVPPTIGDLEHLLELNLSKNHLIGSVPAEFG
NLRSVQVIDISSNNLSGYLPEELGQL
QNLDSLILNNNNLVGEIPAQLANCFSLITLNLSYNNFSGHVPSAKNFSKFPMDSFEGNPMLYVYCQDSSC
GHAHGTKVNISRTAVACIILGFIILLCIML
LAIYKTNKPLPPEKGSDKPVQGPPKLVVLQMDMASHTYEEIMRLTENFSEKYIIGYGASSTVYKCDLKSGK
AIAVKRLYSQYNHSLREFETELETIGSIR
HRNLVSLHGFSLSPHGNLLFYDYMENGSLWDLLHGPSKKVKLDWDTRLKIAVGAAQGLAYLHHDCNPRI
VHRDVKSSNILLDENFEAHLSDFGIAKCVPA
AKSHASTYVLGTIGYIDPEYARTSRLNEKSDVYSFGIVLLELLTGKKAVDNESNLHQLILAKADDNTVMEAV
DSEVSVTCTDMNLVRKAFQLALLCTKRH
PADRPTMHEVARVLLSLLPAPAVKPPTTKGAAGDYTRFLATTTADMKHDVSVDIGDNSSSDEQWFVRFG
EVISKHTMS*
>Sevir.4G085800
MLVRSSVAMRTTAAATSRALVALLLVAVAVADDGSTLLEIKKSFRNVGNVLYDWAGDDYCSWRGVVCD
NVTFAVAALNLSGLNLGGEISPAVGSLKSLSS
IDLKSNGLSGQIPDEIGDCSSLRTLDLSFNNLDGDIPFSISKLKQLENLILKNNQLVGAIPSTLSQLPNLKILDL
AQNKLTGEIPRLIYWNEVLQYLGLR
GNHLEGSLSPDMCQLTGLWYFDVKNNSLTGVIPDTIGNCTSFQVLDLSYNQFTGPIPFNIGFLQVATLSLQ
GNKFTGPIPSVIGLMQALAVLDLSYNQLS
GPIPSILGNLTYTEKLYMQGNRLTGSIPPELGNMSTLHYLELNDNQLTGSIPPELGKLTGLFDLNLANNNLE
GPIPDNLSSCVNLNSFNAYGNKLNGTIP
LSLRKLESMTYLNLSSNFLSGPIPIELSRINNLDTLDLSCNMMTGPIPPAIGSLEHLLRLNLSKNGFLGFIPAEF
GNLRSVMEIDLSYNHLGGLIPQELG
MLQNLMLLKLENNNITGDVSSLMNCFSLNILNVSYNNLAGVVPTDNNFSRFSPDSFLGNPGLCGYWLGS
SCRSTNHQEKPPISRAAILGIAVGGLVILLM
ILVAVCRPHRPPVFKDVTVSKPDMHAVAVSNGPPKLVILHMNMALHAYEDIMRMTENLSEKYIIGYGAS
STVYKCVLKNCKPVAIKKLYAHYPQSLKEFE
TELETVGSIKHRNLVSLQGYSLSPVGNLLFYDYMESGSLWDVLHESSSKKKKLDWESRLRIALGAAQGLAY
LHHDCSPRIIHRDVKSKNILLDKDYEAHL
TDFGIAKSLCVSKTHTSTYVMGTIGYIDPEYARTSRLNEKSDVYSYGIVLLELLTGKKPVDNECNLHHLILSKT
ASNEVMETVDPDIGDTCKDLGEVKKV
FQLALLCTKRQPSDRPTMHEVVRVLDCLVNPDPPPKPPAHQPSGQSPLPPPSSVPSYINEYVSLRGTGALS
CANSTSTSDAELFLKFGEAISQNTE*
>Sevir.4G240700
MACHRRARLLPVVAVLAAALVALSVGPAAALSPDGKALLSLLPGAAPSPVLPSWDPKAATPCSWQGVTC
SPQSRVVSLSLPNTFLNLSSLPPPLAALSSL
QLLNLSTCNISGTIPPSYASLSALRVLDLSSNALTGDIPDELGALSELQFLLLNSNRLTGGIPRSLANLSALQVL
CVQDNLLNGTIPASLGALAALQQFR

VGGNPALSGPIPPSLGALSNLTVFGAAATALSGPIPEELGNLVNLQTLALYDTAVSGSIPAALGGCVELRNL
YLHMNKLTGPIPPELGRLQKLTSLLLWG
NALSGKIPPELSNCSALVVLDLSGNRLAGEVPAALGRLGALEQLHLSDNQLTGRIPPELSNLSSLTALQLDK
NGFSGAIPPQLGELKSLQVLFLWGNALS
GTIPPSLGNCTELYALDLSKNRLSGGIPDEVFALQKLSKLLLLGNALSGPLPPTVANCVSLVRLRLGENKLAG
DIPREIGKLQNLVFLDLYSNRFTGTLP
AELANVTVLELLDVHNNSFTGSIPPQFGELMNLEQLDLSMNNLTGEIPASFGNFSYLNKLILSGNNLSGPL
PKSIRNLQKLTMLDLSNNSFSGPIPPEIG
ELSSLGISLDLSSNRFVGELPEEMSGLTQLQSLNLASNGLYGSISVLGALTSLTSLNISYNNFSGAIPVTPFFKT
LSSNSYIGNANLCESYDGHTCASDM
VRRSALKTVKTVILVCAVLGSVTLLLVVVWILINRNRKLAGEKAMSLSGAGGDDFSNPWTFTPFQKLNFSI
DNILACLRDENVIGKGCSGVVYRAEMPNG
EIIAVKKLWKAGKDEPIDAFAAEIQILGHIRHRNIVKLLGYCSNRSVKLLLYNYIPNGNLQQLLKENRSLDWD
TRYKIAVGTAQGLAYLHHDCVPAILHR
DVKCNNILLDSKYEAYLADFGLAKLMNSPNYHHAMSRIAGSYGYIAPEYAYTSNITEKSDVYSYGVVLLEILS
GRSAIEPVVGEASLHIVEWAKKKMGSY
EPAVNILDPKLRGMPDQLVQEMLQTLGVAIFCVNAAPAERPTMKEVVALLKEVKSPPEEWAKTSQQPLI
KPGSQQG*
>Sevir.5G116900
MPPGVMGGRRRGRWRSAWAAPPPLLLWCALAMAWVGCALAVDAQGAALLAWKRTLRGDAEEALG
DWRDSDASPCRWTGVSCDAAGRVTGLSLQFVDLHGG
APADLSAVGATLSRLVLTGTNLTGPIPPGLGDQLPVLTHLDLSNNALTGPIPVSLCRPGSKLESLYVNSNRLE
GAIPDAIGNLTALRELIFYDNQLEGTI
PASIGQMASLEVIRGGGNKNLQGALPPEIGDCSNLTMLGLAETSISGPLPASLGKLKSLDTIAIYTALLSGPI
PPELGDCSSLTNIYLYENALSGSIPPQ
LGKLRNLKNLLLWQNNLVGVIPPELGACTGLTVLDLSMNGLIGHIPASLGNLTSLQELQLSVNKVSGPIPAE
LARCINLTDLELDNNQISGGIPAEIGKL
TALRMLYLWANQLTGSIPPAIGGCVSLESLDLSQNALTGPIPRSLFQLPRLSKLLMIDNTLSGEIPPEIGNCT
SLVRFRASGNHLAGAIPPEVGKLGNLS
FLDLSSNRLSGAIPADIAGCRNLTFVDLHGNAITGVLPPGLFHDMPSLQYLDLSYNSISGVIPSDIGRLGSLT
KLVLGGNRLTGQIPPEIGSCSRLQLLD
LGGNALSGAIPASIGKIPGLEIALNLSCNGLSGAIPKEFAGLVRLGVLDVSHNQLSGDLQPLSALQNLVALNI
SFNSFAGRAPATAFFAKLPTSDVEGNP
GLCLTRCPGDASDRERASRRAAKVATAVLLSALVALLAAAAFLLVGRRRGSARGAGDGDDKDAEMLPP
WDVTLYQKVEISVGDVARSLTPANVIGKGWSG
SVYRAAVPSTGGVTIAVKKFRSCDEASAEAFACEVGVLPRVRHRNIVRLLGWAANRRTRLLFYDYLPNGTL
GGLLHGGGAVAEWEVRLAIAVGVAEGLAY
LHHDCVPAILHRDVKADNILLGERYEACLADFGLARVADDGANSSPPPFAGSYGYIAPEYGCMSKITTKSD
VYSFGVVLLEVITGRRPVEAAFGEGRSVV
QWVREHLHQKRDPAGVVDPRLQGRPDAQVQEMLQALGIALLCASARPEDRPTMKDVAALLRGLRNDD
GAEARKVSGGGSGGARLDSAKWAADAPSPTKPT
ALPRPAQAQSQSQSSSLAYSM*
>Sevir.7G168000
MEPRALLLGVALAFLLASGSQGLNHEGWLLLALKSQMVDTLHHLDSWDARHPTPCAWRGVNCSSAPV
PAVVSLDLNNMNLSGTIAPSIGGLAELTHLDLS
FNGFGGPIPAQIGNLSKLEVLNLFNNNFVGIIPPEVGKLAKLVTLNLCNNKLYGPIPDEIGNMASLEELVGY
SNNLTGSLPHSLGKLKNLKNIRLGQNLI

SGNIPVEIGECLNITVFGLAQNKLEGPLPKEIGRLSLMTDLILWGNQLSGVIPPEIGNCTSLGTVALYDNNLF
GPIPATIGNITNLQKLYLYRNSLNGTI
PSEIGNLSLAREIDFSENFLTGGIPKELGNIPELNLLYLFQNQLTGSIPTELCGLRNLSKLDLSINSLTGTIPSGF
QYMRTLIQLQLFNNKLSGNIPPRF
GIYSRLWVVDFSNNSITGQIPKDLCRQSNLILLNLGSNKLTGNIPRGITNCRPLVQLRLGDNSLTGSFPTDLC
NLVNLTTVELGRNKFSGPIPPQIGDCK
SLQRLDLTNNYFTSELPREIGNLSKLVVFNISSNRLGGNIPLEIFNCTVLQRLDLSQNNFEGSLPNEVGRLPQ
LELLSFSDNRLAGQIPPILGKLSHLTA
LQIGGNLLSGEIPKELGLLSSLQIAMNLSYNNLSGNIPSELGNLALLESLFLNNNKLTGEIPTTFANLSSLLELN
VSYNYLSGALPSIPLFDNMAATCFI
GNKGLCGGQLGRCGSQSSSSSQSSNSVGPPLGKIIAIVAAVIGGISLILIAIIVYHMRKPMETVAPLQDKQLF
SGGSNMHVSVKEAYTFQELVAATNNFD
ESCVIGRGACGTVYRAILKTGQTIAVKKLASNREGSNTDNSFRAEILTLGKIRHRNIVKLYGFIYHQGSNLLLY
EYMSRGSLGELLHGQSSSSLDWETRF
MIALGAAEGLTYLHHDCKPRIIHRDIKSNNILLDENFEAHVGDFGLAKVIDMPYSKSMSAIAGSYGYIAPEY
AYTMKVTEKCDIYSYGVVLLELLTGRAP
VQPLEQGGDLVTWVKNYIRDNSLGPGVLDKNLDLEDQSVVDHMIEVLKIALVCTSLSPYERPPMRHVVV
MLSESKDRTRVSSASSPASDDSSKKGSS*
>Sevir.9G072100
MRLLPLLLLLALAAGAAGAAGGTDADALLAAKAALSDPTGALRSWNATSPDHCAWAGVTCAPPGGGR
GGGIVVGLDVSGLNLSGALPQALSRLHGLQRLS
VAANALYGPIPPSLARLQQLVHLNLSNNAFNGSFPPALARLRGLRVLDLYNNNLTSATLPLEVTQMPMLR
HLHLGGNFFSGEIPPEYGRWPRLQYLAVSG
NELSGRIPPELGNLTTLRELYIGYYNSYTGGLPPELGNLTELVRLDAANCGLSGEIPPELGRLQNLDTLFLQV
NGLTGSIPSELGHLKSLSSLDLSNNAL
TGEIPESFSELKNLTLLNLFRNKLRGDIPDFVGDMPSLEVLQLWENNFTGGVPRRLGRNGRLQLLDLSSNK
LTGTLPPELCAGGKLQTLIALGNFLFGAI
PDSLGQCKSLSRVRLGENYLNGSIPKGLFELPKLTQVELQDNLLTGNFPAVIGVAAPNLGEISLSNNQLTGA
LPASLGSFSGIQKLLLDRNSFSGAVPPE
IGRLQQLSKADLSSNKFEGGVPPEIGKCRLLTYLDMSQNNLSGKIPPAISGMRILNYLNLSRNHLDGEIPPSI
ATMQSLTAVDFSYNNLSGLVPGTGQFT
YFNATSFVGNPGLCGPYLGPCRPGIAGADHTPHGHGGLTNTVKLLIVLGLLVCSIAFAAAAILKARSLKKAS
EARVWKLTAFQRLDFTSDDVLDCLKEEN
IIGKGGAGIVYKGAMPNGELVAVKRLPAMGRGSSHDHGFSAEIQTLGRIRHRHIVRLLGFCSNNETNLLVY
EYMPNGSLGEMLHGKKGGHLHWDTRYNIA
IEAAKGLCYLHHDCSPLILHRDVKSNNILLDSNFEAHVADFGLAKFLQDSGASECMSAIAGSYGYIAPEYAY
TLKVDEKSDVYSFGVVLLELVTGRKPVG
EFGDGVDIVQWAKMMTDSSKEQVMKILDPRLSTVPLHEIMHVFYVALLCTEEQSVQRPTMREVVQILSE
LPKPSTKQGEEVPNACDGSASGPLHPAPAGS
NEAPTGEARDQQQQQTSSPSSPPPDLISI*
>Sevir.9G226700
MRYDSLAWPAPFFSFAAMCESTRKAETGHLIGTPTNHEHARQASRPKFSRPVLSFVDGSGTLLLPENDQS
TFNHSSPKTTHQPAVNAASEQAQDKPRAPS
TPRPRPAPPHRTHGHGHGHVALRSTASARPMPTPPPAPARLLHLAAPLLVVLALAAGVANAATPPSPAD
ALLAWKSSLGDPPALSTWADAASLCTGWRGV
ACDAAGRVTSLRLRGLGLTGGLDALNAAALPGLTSLDLNGNNLAGPIPASLSRLRALAALDLGSNGLNGTI
PPQLGDLSGLVDLRLYNNNLAGAIPHQLS

KLPKIVHFDLGSNYLTNPDKFESMPTVSFLSLYLNYLNGSFPEFVLRSSNVTYLDLSQNTFSGPIPDSLPERLP
NLRWLNLSANAFSGKIPASVARLTRL
QDLHIGGNNLTGGVPEFLGSMSKLRVLELGGTQLGGRLPPVLGRLKMLQRLDVKNAGLVSTLPPELGNLS
NLDFVDLSGNHLSGSLPASFVGMRKMREFG
VSFNNLTGEIPGGLFTGWPELISFQVQSNSLTGKIPPALGKATKLRILFLFSNKLTGSIPPELGELANLNELDL
SVNSLTGPIPNSFGNLKQLTRLALFF
NGLTGEIPPEIGNMTALQILDVNTNHLEGELPSTISSLRNLQYLALFDNNLSGTIPPDLGAGLALTDVSFAN
NSFSGELPRNLCNGFTLQNFTANHNKFS
GKLPPCMKNCSELYRVRLEGNQFTGDISEVFGVHPNMDYLDVSGNKLTGRLSDDWGQCTNITRLHMDG
NRISGGIPVAFWSMTSLQDLSLAGNNLTGVIP
PELGYLSVLFNFNLSHNSFSGPIPTSLGNNSKLQKVDFSGNMLNGTIPVGISNLGSLTYLDLSKNKLSGQIPS
ELGNLVQLQIVLDLSSNSLSGPIPSNL
VKLMNLQKLNLSRNELSGSIPAGFSRMSSLETVDFSYNQLTGEIPSGSAFQNSSAAAYIGNLGLCGNVQGI
PSCDRNASSGHRKRTVIEIVLSVVGAVLL
AAIVACLILSCCRRPREQKVLEASTSDPYECMIWEKEGKFTFLDIVNATDSFNESFCIGKGGFGSVYKAELTS
GQVVAVKRFHVAETGDISEASKKSFEN
EIKALTEVRHRNIVKLHGFCTSGDYMYLVYEYLERGSLGKTLYSEEGKKKFDWGMRVKVVQGVAHALAYL
HHDCNPAIVHRDTTVNNILLESEFEPRLSD
FGTAKLLGSASTNWTSVAGSYGYMAPELAYTMRVTEKCDVYSFGVVALEVMMGKHPGDLLTSLPAISAS
KEDDLLLQDVLDQRLDPPMGEIAEEIVFVVR
IALACTRANPESRPSMRSVAQEISAHTQACLSEPFRQITVSKLTDYQK*
>Sevir.9G291100
MSLTLLTLLLLIAIPPATSATQPASNSSADVLLSFLAALPPAAQRLLLPSWNTAASGNGNSTAAGPHCAFLG
VTCSAAGAVAALNLSGAGLSGDLAASAP
QLCSLPELAALDLSGNNFTGAIPLELAACSALSALLLGRNGLSGALPPELLSSRQLKNIDLNSNALTGEIPAPS
AGGFSLLQYLDLSNNNFSGAIPLELA
ALPALSYLDLSTNKLSGPMPDFPVHCVLKFLNVDSNKIDGKLPRSLGNCGNLTRLYLSNNKISGSVPDFFAS
MPGLEKLFLSNNSFTGEFPASIGELVNL
EKLMVSANGFTGPVPESIGKCHSLTMLWMHSNRFTGSIPAAIGNLVSLQWFTIKDNLITGTIPPEIGKCQE
LTWLELHNNSLSGVIPPEITQLTKLQVLS
LFGNRLHGQVPAALWQMPYMEELALSYNNLTGEVPAEITLMRNLRELILAYNNFTGEIPQALGLNTTQG
LQRIDLTGNRFRGEIPPGLCTGGRLAVLDLG
HNQFTGAIPSEIWKCQSLWRVILGNNLFSGSLLPSELGTNTGWSFVELSGNLFEGRVPSVFGSWRNLTVL
DLSSNRFSGPIPRELGALSILGNLNLSSNM
LSGPIPHELGNCKRLVRLDLQYNYLNGSISSEIIAHDSLQTLMLSGNKLTGKIPDVFTGTQGLLELHLGANSL
EGPIPESLGKLQFISKIINISNNRLSN
EIPSSLGNLQMLEMLDLSKNSLSGPIPSQLSNMMALSFVNVSFNELSGQLPAGWVKLAERSPEGFLGNP
QLCIQSNNAPCSRNQSAKRIRKNTRIIVALL
VSALAIMAAGLFLVHYMVKRSQRQLAKHVSVRGLDTTEELPKDITFDDILRATDNWSEKYVIGRGRHGTV
YRTEFAPGRQWAVKTVDLSQFKFPIEMKIL
NMVKHRNIVKMEGYCIRGNFGIILSEYMPQGTLFELLHGRKLQVALDWNVRHQIALGTAQGLSYLHHDC
VPMIVHRDVKSSNILMDADLVPKITDFGMGK
IIDDDDADATVSVVVGTLGYIAPEHGYNTRLTEKSDVYSYGVVLLELMCRKMPVDPAFGDGVDIVAWMT
SKLKSADHCSLMNYLDEEIMYWPGDEQAKAL
DLLDLAMSCTQVSFQSRPSMREVVSTLMRIEDEYITNE*
>Sevir.9G484200

MAPSMAAASTLLLPVLLLIATANHCADAADSPSSPDAAALLNLSAALTDPSGYLATHWTPDTALCSWPRV
SCDVADRRVISLDLSGLNLSGPIPAAALSS
LPLLQTLNLSNNILNSTFPDEIIASLRSLRVLDLYNNNLTGPLPAALPKLTDLVHLHLGGNFFSGSIPRSYGQ
WTRIRYLALSGNELTGEIPPELGNLST
LRELYLGYFNSFTGGIPPELGRLRALVRLDMANCGISGEIPPEVANLTSLDTLFLQINALTGRLPTEIGAMGA
LKSLDLSNNLFVGAIPASFASLKNLTL
LNLFRNRLAGEIPEFIGELPNLEVLQLWENNFTGGIPPNLGVAATRLKIVDVSTNKLTGVLPSELCAGEQLE
TFIALGNSLFGGIPDGLAGCPSLTRIRL
GENYLNGTIPAKLFTLPNLTQIELHDNLLSGELSLEAGKVSSSIGELSLFNNRLSGQVPTGIGGFVGLQKLLLA
GNRLSGELPPEIGKLQQLSKADLSGN
LISGEVPPAIGRCRLLTFLDLSGNKLSGRIPPELASLRILNYLNVSHNALEGEIPSAIAGMQSLTAVDFSYNNL
WGEVPATGQFAYFNATSFAGNDELCG
AFLSPCRSHGVATSAFGSLSSTSKLLLVLGLLALSIIFAAAAVLKARSLKRSAEARAWRLTAFQRLDFAVDDV
LDCLKEENVIGKGGSGIVYKGAMPGGA
VVAVKRLPAIGRAGAAHDDYGFSAEIQTLGRIRHRHIVRLLGFAANRETNLLVYEYMPNGSLGEVLHGKK
GGHLQWATRFKIAVEAAKGLCYLHHDCSPP
ILHRDVKSNNILLDADFEAHVADFGLAKFLRGNAGGSECMSAIAGSYGYIAPEYAYTLKVDEKSDVYSFGV
VLLELITGRKPVGEFGDGVDIVQWVRMVT
GSSKEGVMKIADPRLSTVPLYELTHVFYVAMLCVAEQSVERPTMREVVQILADMPGSTSTSIDAPLVIEPK
EDASSEKPPRQQEGPHDSPPQHDLLSI*
>Bradi1g07180
MHLRLLPLLLVLLLAGAGVGAAADGDADALLAAKAALSDPTGALASWAAPKKNESAAHCAWAGVTCGP
RGTVVGLDVGGLNLSGALPPALSRLRGLLRLD
VGANAFFGPVPAALGHLQFLTHLNLSNNAFNGSLPPALACLRALRVLDLYNNNLTSPLPLEVAQMPLLRH
LHLGGNFFSGQIPPEYGRWARLQYLAVSGN
ELSGTIPPELGNLTSLRELYLGYYNSYSGGLPAELGNLTELVRLDAANCGLSGEIPPELGKLQKLDTLFLQVN
GLSGSIPTELGYLKSLSSLDLSNNVLT
GVIPASFSELKNMTLLNLFRNKLRGDIPDFVGDLPSLEVLQLWENNFTGGVPRRLGRNGRLQLVDLSSNKL
TSTLPAELCAGGKLHTLIALGNSLFGSIP
DSLGQCKSLSRIRLGENYLNGSIPKGLFELQKLTQVELQDNLLTGNFPAVVGVAAPNLGEINLSNNQLTGT
LPASIGNFSGVQKLLLDRNSFSGVMPAEI
GRLQQLSKADLSSNSIEGGVPPEIGKCRLLTYLDLSRNNLSGDIPPAISGMRILNYLNLSRNHLDGEIPPSIAT
MQSLTAVDFSYNNLSGLVPVTGQFSY
FNATSFVGNPSLCGPYLGPCRPGIADTGHNTHGHRGLSSGVKLIIVLGLLLCSIAFAAAAILKARSLKKASDA
RMWKLTAFQRLDFTCDDVLDSLKEENI
IGKGGAGTVYKGSMPNGDHVAVKRLPAMVRGSSHDHGFSAEIQTLGRIRHRHIVRLLGFCSNNETNLLV
YEYMPNGSLGELLHGKKGEHLHWDTRYKIAI
EAAKGLCYLHHDCSPLILHRDVKSNNILLDSDFEAHVADFGLAKFLQDTGASECMSAIAGSYGYIAPEYAYT
LKVDEKSDVYSFGVVLLELVTGRKPVGE
FGDGVDIVQWVKMMTDSNKEQVMKILDPRLSTVPLHEVMHVFYVALLCIEEQSVQRPTMREVVQILSE
LPKPASNQGEELPHFDEGSASSPPAPTSSSEA
APTTDAKDQQLHQTGSESSAPPDLISI*
>Bradi1g26900
MARAAPRFSSVMPAAWLLLVVLVSCTAAAAGDDGDVLLDVKAAFSQDPEGVLDGWSADAAGSLGFCS
WSGVTCDAAGLRVSGLNLSGAGLAGPVPSALSR
LDALQTIDLSSNRLTGSIPPALGRLGRSLEVLMLYSNDLASEIPASIGRLAALQVLRLGDNPRLSGPIPDSLGE
LSNLTVLGLASCNLTGAIPRRLFARL

SGLTALNLQENSLSGPIPAGIGAIAGLQVISLANNNLTGVIPPELGSLAELQKLNLGNNTLEGPIPPELGALG
ELLYLNLMNNSLTGRIPRTLGALSRVR
TLDLSWNMLTGGIPAELGRLTELNFLVLSNNNLTGRIPGELCGDEEAESMMSLEHLMLSTNNLTGEIPGTL
SRCRALTQLDLANNSLSGNIPPALGELGN
LTDLLLNNNSLSGELPPELFNLTELGTLALYHNELTGRLPGSIGNLRSLRILYAYENQFTGEIPESIGECSTLQ
MMDFFGNQLNGSIPASIGNLSRLTFL
HLRQNELSGEIPPELGDCRRLEVLDLADNALSGEIPGTFDKLQSLEQFMLYNNSLSGAIPDGMFECRNITR
VNIAHNRLSGSLVPLCGSARLLSFDATNN
SFQGGIPAQLGRSASLQRVRLGSNALSGPIPPSLGRIAALTLLDVSCNALTGGIPDALSRCAQLSHVVLNNN
RLSGPVPAWLGTLPQLGELTLSTNEFSG
AMPVELSNCSKLLKLSLDGNLINGTVPHEIGRLASLNVLNLARNQLSGPIPATVARLGNLYELNLSQNHLSG
RIPPDMGKLQELQSLLDLSSNDLIGKIP
ASLGSLSKLEDLNLSHNALVGTVPSQLAGMSSLVQLDLSSNQLEGRLGDEFSRWPEDAFSDNAALCGNHL
RGCGDGVRRGRSALHSASIALVSTAVTLTV
VLLVIVLVLMARRRGRMSGEVNCTGFSSSLGNTNRQLVIKGSARREFRWEAIMEATANLSDQFAIGSGGS
GTVYRAELSTGETVAVKRIASMDSDMLLHD
KSFAREIKILGRVRHRHLVKLLGFLAHGADRGGSMLIYEYMENGSLYDWLHGGGGEGGKKKRALSWDAR
LKVAAGLVQGVEYLHHDCVPRVVHRDIKSSN
LLLDADMEAHLGDFGLAKAVAENRQGAKECTESASFFAGSYGYMAPECAYSLKATEKSDVYSTGIVLMEL
VTGLLPTDKTFGGDVDMVRWVQSRVEAPSQ
ARDQVFDPALKPLAPREESSMAEALEVALRCTRPAPGERPTARQISDLLLHISMDYYRTGEHKR*
>Bradi1g46450
MATTAAASGALIAILLLLVAGAGAVGDDGSTLLEIKKSFRNVENVLYDWSGDDYCSWRGVLCDNVTFAV
AALNLSGLNLEGEISPAVGSLKSLVSIDLKS
NGLTGQIPDEIGDCSSIKTLDLSFNNLDGDIPFSVSKLKHLETLILKNNQLIGAIPSTLSQLPNLKILDLAQNKL
SGEIPRLIYWNEVLQYLGLRGNHLE
GSLSPDICQLTGLWYFDVKNNSLTGEIPETIGNCTSFQVLDLSYNQFTGSIPFNIGFLQIATLSLQGNKFTGP
IPSVIGLMQALAVLDLSYNQLSGPIPS
ILGNLTYTEKLYMQGNRLTGTIPPELGNMSTLHYLELNDNQLTGSIPSELGKLTGLYDLNLANNNLEGPIPN
NISSCVNLNSFNAYGNKLNGTIPRSLCK
LESMTSLNLSSNYLTGPIPIELSRINNLDVLDLSCNMITGPIPSAIGSLEHLLTLNLSKNGLVGFIPAEFGNLRSI
MEIDLSNNHLAGLIPQEIGMLQNL
MLLKLESNNITGDVSSLMNCFSLNILNISYNNLVGAVPTDNNFSRFSPDSFLGNPGLCGYWLGSSCRSPN
HEVKPPISKAAILGIAVGGLVILLMILVAV
CRPHRPHVSKDFSVSKPVSNVPPKLVILNMNMALHVYEDIMRMTENLSEKYIIGYGASSTVYKCVLKNCR
PVAIKKLYAHYPQSLKEFQTELETVGSIKH
RNLVSLQGYSLSPVGNLLFYEYMENGSLWDVLHEGPSKKKKLDWETRLRIALGAAQGLAYLHHDCSPRII
HRDVKSKNILLDNDYEAHLTDFGIAKSLCV
SKTHTSTYVMGTIGYIDPEYARTSRLNEKSDVYSYGIVLLELLTGKKPVDNECNLHHSILSKTASNAVMETV
DPDIADTCQDLGEVKKVFQLALLCTKRQ
PSDRPTMHEVVRVLDCLVRPDPPLKPVQTSSSLQPVPSYVNEYVSLRGAGALSCATSSSTSDAELFLKFGE
AISQNTE*
>Bradi1g49950
MAASNSRGAPSGRWAAAAMVVLMVVLGAAAVEGGDGEALMAVKAGFGNAANALVDWDGGRDHY
CAWRGVTCDNASFAVLALNLSNLNLGGEISPAVGELK
SLQLVDLKGNKLTGQIPDEIGDCVSLKYLDLSFNLLYGDIPFSISKLKQLEDLILKNNQLTGPIPSTLSQIPNLK
TLDLAQNQLTGDIPRLIYWNEVLQY

LGLRGNSLTGTLSPDMCQLTGLWYFDVRGNNLTGSIPESIGNCTSFEILDISYNQISGEIPYNIGFLQVATLS
LQGNRLTGKIPDVIGLMQALAVLDLSE
NELVGPIPPILGNLSYTGKLYLHGNKLTGEVPPELGNMTKLSYLQLNDNELVGTIPAELGKLEELFELNLAN
NNLEGPIPTNISSCTALNKFNVYGNRLN
GSIPAGFQNLESLTYLNLSSNNFKGQIPSELGHIINLDTLDLSYNEFSGPIPATIGDLEHLLQLNLSKNHLNGP
VPAEFGNLRSVQVIDISNNAMSGYLP
QELGQLQNLDSLILNNNSFVGEIPAQLANCFSLNILNLSYNNFSGHVPLAKNFSKFPMESFLGNPMLHVYC
KDSSCGHSRGPRVNISRTAIACIILGFII
LLCAMLLAIYKTNRPQPLVKGSDKPIPGPPKLVILQMDMAIHTYEDIMRLTENLSEKYIIGYGASSTVYKCVL
KNGKAIAVKRLYSQYNHGAREFETELE
TVGSIRHRNLVSLHGFSLSPHGNLLFYDYMENGSLWDLLHGPSKKVKLDWDTRLRIAVGAAQGLAYLHH
DCNPRIVHRDVKSSNILLDEHFEAHLSDFGI
AKCVPAAKTHASTYVLGTIGYIDPEYARTSRLNEKSDVYSFGIVLLELLTGKKAVDNDSNLHQLILSRADDNT
VMEAVDSEVSVTCTDMGLVRKAFQLAL
LCTKRHPMDRPTMHEVARVLLSLMPAPALKPSYTTASKTVDYTRYLATTPDLNHDGTDIGNNSSSDEQW
FVRFGEVISKHTM*
>Bradi1g57900
MLLPLVLLLAALAAIDPASGSDADALLAAKAALDDPTGALASWTSTSPNPCAWSGVSCAAGSNSVVSLDL
SGRNLSGRIPPSLSSLPALILLDLAANALS
GPIPAQLSRLRRLASLNLSSNALSGSFPPQLSRRLRALKVLDLYNNNLTGPLPVEIAAGTMPELSHVHLGGN
FFSGAIPAAYGRLGKNLRYLAVSGNELS
GNLPPELGNLTSLRELYIGYYNSYSGGIPKEFGNMTELVRFDAANCGLSGEIPPELGRLAKLDTLFLQVNGL
TDAIPMELGNLGSLSSLDLSNNELSGEI
PPSFAELKNLTLFNLFRNKLRGNIPEFVGDLPGLEVLQLWENNFTGGIPRHLGRNGRFQLLDLSSNRLTGTL
PPELCAGGKLHTLIALGNSLFGAIPESL
GECRSLARVRLGENFLNGSIPEGLFQLPNLTQVELQGNLLSGGFPAMAGASNLGGIILSNNQLTGALPASI
GSFSGLQKLLLDQNAFSGPIPPEIGRLQQ
LSKADLSGNSFDGGVPPEIGKCRLLTYLDVSRNNLSAEIPPAISGMRILNYLNLSRNHLEGEIPATIAAMQSL
TAVDFSYNNLSGLVPATGQFSYFNATS
FLGNPGLCGPYLGPCHSGSAGADHGGRTHGGLSSTLKLIIVLVLLAFSIVFAAMAILKARSLKKASEARAWK
LTAFQRLEFTCDDVLDSLKEENIIGKGG
AGTVYKGTMRDGEHVAVKRLSTMSRGSSHDHGFSAEIQTLGSIRHRYIVRLLGFCSNNETNLLVYEYMPN
GSLGELLHGKKGCHLHWDTRYKIAVEAAKG
LCYLHHDCSPPILHRDVKSNNILLDSDFEAHVADFGLAKFLQDSGTSECMSAIAGSYGYIAPEYAYTLKVDE
KSDVYSFGVVLLELITGKKPVGEFGDGV
DIVQWIKMMTDSSKERVIKIMDPRLSTVPVHEVMHVFYVALLCVEEQSVQRPTMREVVQILSEPPKLIPK
QGEELPGSGEGDELDPAIPAETVESVSNEA
QEQQQLSPKSSLPPNLISI*
>Bradi1g58460
MARFLLPLLLFAAAACSAVSASEEEPAAAALREFKRALADIDGRLSSWDNSTGRGPCEWAGIACSSSGEVT
GVKLHGLNLSGSLSASAAAAICASLPRLA
VLNVSKNALSGPIPATLSACHALQVLDLSTNSLSGAIPPQLCSSLPSLRRLFLSENLLSGEIPAAIGGLAALEEL
VIYSNNLTGAIPPSIRLLQRLRVVR
AGLNDLSGPIPVEITECAALEVLGLAQNALAGPLPPQLSRFKNLTTLILWQNALTGEIPPELGSCTSLEMLAL
NDNGFTGGVPRELGALSMLVKLYIYRN
QLDGTIPKELGSLQSAVEIDLSENRLVGVIPGELGRISTLQLLHLFENRLQGSIPPELAQLSVIRRIDLSINNLT
GKIPVEFQKLTCLEYLQLFNNQIHG

VIPPLLGARSNLSVLDLSDNRLKGRIPRHLCRYQKLIFLSLGSNRLIGNIPPGVKACMTLTQLRLGGNKLTGS
LPVELSLLQNLSSLEMNRNRFSGPIPP
EIGKFKSMERLILAENYFVGQIPASIGNLAELVAFNVSSNQLAGPVPRELARCSKLQRLDLSRNSFTGIIPQE
LGTLVNLEQLKLSDNNLTGTIPSSFGG
LSRLTELQMGGNLLSGQVPVELGKLNALQIALNISHNMLSGEIPTQLGNLRMLEYLYLNNNELEGKVPSSF
GELSSLMECNLSYNNLVGPLPDTMLFEHL
DSTNFLGNDGLCGIKGKACPASLKSSYASREAAAQKRFLREKVISIVSITVILVSLVLIAVVCWLLKSKIPEIVS
NEERKTGFSGPHYFLKERITYQELL
KATEGFSEGAVIGRGACGIVYKAVMPDGRRIAVKKLKCQGEGSSVDRSFRAEITTLGNVRHRNIVKLYGFC
SNQDSNLILYEYMENGSLGEFLHGKDAYL
LDWDTRYRIAFGAAEGLRYLHSDCKPKVIHRDIKSNNILLDEMMEAHVGDFGLAKIIDISNSRTMSAVAGS
YGYIAPEYAFTMKVTEKCDIYSFGVVLLE
LVTGQCPIQPLEKGGDLVNLVRRTMNSMAPNSDVFDSRLNLNSKRAVEEMTLVLKIALFCTSESPLDRPS
MREVISMLIDARASSCDSYSSPASEPPTED
ESHFKLQRQVHPCTKGVYTA*
>Bradi1g69097
MARSMAMAATPTFLLLVLIFLLATATCSTAAGSSPSNASLEAAALANLSAALADPSGYLSAHWTPVTPLCS
WPRLSCDAAGSRVISLDLSALNLSGPIPA
AALSSLTHLQSLNLSNNLFNSTFPEALIASLPNIRVLDLYNNNLTGPLPSALPNLTNLVHLHLGGNFFSGSIP
GSYGQWSRIRYLALSGNELTGAVPPEL
GNLTTLRELYLGYFNSFTGGIPRELGRLRELVRLDMASCGISGTIPPEVANLTSLDTLFLQINALSGRLPPEIG
AMGALKSLDLSNNLFVGEIPASFVSL
KNMTLLNLFRNRLAGEIPGFVGDLPSLEVLQLWENNFTGGVPAQLGVAATRLRIVDVSTNKLTGVLPTEL
CAGKRLETFIALGNSLFGGIPDGLAGCPSL
TRIRLGENYLNGTIPAKLFSLQNLTQIELHDNLLSGELRLEAGEVSPSIGELSLYNNRLSGPVPAGIGGLSGLQ
KLLIAGNILSGELPPAIGKLQQLSKV
DLSGNRISGEVPPAIAGCRLLTFLDLSGNKLSGSIPTALASLRILNYLNLSNNALDGEIPASIAGMQSLTAVDF
SYNGLSGEVPATGQFAYFNSTSFAGN
PGLCGAFLSPCRTTHGVATSSAFGSLSSTSKLLLVLGLLALSIVFAGAAVLKARSLKRSAEARAWRITAFQRL
DFAVDDVLDCLKDENVIGKGGSGVVYK
GAMPGGAVVAVKRLLSAALGRSAGSAHDDYGFSAEIQTLGRIRHRHIVRLLGFAANRETNLLVYEYMPN
GSLGEVLHGKKGGHLQWATRYKIAVEAAKGL
CYLHHDCSPPILHRDVKSNNILLDADFEAHVADFGLAKFLHGSNAGGSECMSAIAGSYGYIAPEYAYTLKV
DEKSDVYSFGVVLLELIAGRKPVGEFGDG
VDIVQWVRMVAGSTKEGVMKIADPRLSTVPIQELTHVFYVAMLCVAEQSVERPTMREVVQILTDLPGTT
TSMSLPPPDLEEGREENQGHEQQQGEPHDSP
AHQDLLSI*
>Bradi2g56750
MPTSPLQIYFWCILLFANVGISTSLPLETDALLDIKSHLEDPEKWLHNWDEFHSPCYYYGVTCDKLSGEVIG
VSLSNVSLSGTISPSFSLLRRLHTLELG
ANSISGIIPAALANCTNLQVLNLSMNSLTGQLPDLSPLLKLQVLDLSTNNFSGAFPVWISKLSGLTELGLGE
NNFTEGDVPESIGVLKNLTWLFLGKCNL
RGDIPASVFDLVSLGTLDFSRNQMTGMFPKAISKLRNLWKIELYQNNLTGEIPPELAHLTLLSEFDVSQNEL
TGILPREISNLKNLKIFHIYMNNFYGEL
PEGLGDLQFLESFSTYENQLSGKFPANLGRFSPLNAIDISENYFSGEFPRFLCQNNKLQFLLALNNNFSGEF
PSSYSSCKKLERFRISQNQFAGSIPYGI

WGLPNAVIIDVADNGFIGGISSDIGISANLNQLFVQNNNFSSELPLELGKLSQLQKLIAFNNRFSGQIPTQI
GNLKQLSYLHLEHNALEGSIPPNIGLCN
SLVDLNLAENSLSGNIPDALASLLMLNSLNLSHNMISGEIPQRLQSLKLSYVNFSHNNLSGPVSPQLLMIAG
EDAFSENYDLCVTNISEGWRQSGTSLRS
CQWSDDHHNFSQRQLLAVVIMMTFFLVLLSGLACLRYENNKLEDVSRKRDTESSDGSDSKWIVESFHPP
EVTAEEVCNLDGESLIGYGRTGTVYRLELSK
GRGIVAVKQLWDCIDAKVLKTEINTLRKICHRNIVKLHGFLAGGGSNFLVYEYAVNGNLYDAIRRKFKAGQ
PELDWARRYRIAVGAAKGIMYLHHDCSPA
IIHRDVKSTNILLDEDYEAKLADFGIAKLVETSPLNCFAGTHGYIAPELTYSLKATEKSDVYSFGVVLLELLTER
SPTDQQFDGELDIVSWASSHLAGQN
TADVLDPRVSNYASEDMIKVLNIAIVCTVQVPSERPTMREVVKMLIDI*
>Bradi3g20867
MRAAAPAPARSLRSLASMSQFPLLLLLLFFLPLVTSAPPPPPNTSAAAGTGSTTAILLSFLAALPPAAQRFLL
PTWLRTGVNHTSSPASKRHHHHHCAFL
GVTCSAATTGEVSAVNLSGSGLSGALASSAPRLCALPALAALDLSRNSLTGPVPAALAACSALTELVLAFNL
LSGTVPAELLSSRSLLRKLDLNTNALTG
DIPPSPSMILEYLDLSANSFSGEIPPEFSALPRLTYLDLSNNNLSGPIPEFSAPCRLLYLSLFSNKLAGELPQSLA
NCVNLTVLYLPDNEISGEVPDFFA
AMPNLQKLYLGDNAFTGELPASIGELVSLEELVVSNNWFTGSVPGAIGRCQSLTMLYLNGNRFTGSIPLFI
GNLSQLQMFSAADNGFTGRIPPEVRNCRG
LVDLELQNNSLSGTIPPEIAELSQLQKLYLFNNLLHGPVPPALWRLADMVELYLNNNSLSGEIHSEITHMR
NLREITLYSNSFTGELPQDLGFNTTPGIV
RVDLTGNRFHGAIPPGLCTGGQLAILDLGDNLFDGGFPSEIAKCQSLYRLKLNNNQISGSLPADLGTNRGL
SYVDMSGNRLEGRIPAVIGSWSNLTMLDL
SGNNLLGPIPGELGALSNLVTLRMSSNMLTGLIPHQLGNCKILVCLDLGNNLLNGSLPAEVTTLGSLQNLLL
DRNNFTSAIPDSFTATQALLELQLGDNY
FEGAIPHSLGNLQYLSKTLNISNNRLSSQIPSSLGNLQDLEVLDLSENSLYGPIPPQVSNMISLLVVNLSFNEL
SGQLPASWVKFAARSPEGFSGNPHLC
VRSDIDAPCSSKKQSVKNRTSRNSWIIVALVLPTVVVLVAALFAIHYIVKMPGRLSAKRVSLRSLDSTEELPE
DMTYEDILRATDNWSEKYVIGKGRHGT
VYRTDCKLGKQWAVKTVDLSQCKFPIEMKILNTVKHRNIVRMAGYYIRGNVGLILYEYMPEGTLFELLHER
KPQVALGWMARHQIALGVAQGLSYLHQDC
VPMIVHRDVKSSNILMDVELVPKLTDFGMGKIVGDEDSDATVSVIVGTLGYIAPEHGYSTRLSEKSDVYSY
GVVLLELLCRKMPVDSAFGDGVDIVTWMR
SNLKQADHCSVMSCLDEEIVYWPEDEQAKALHLLDLAISCTEVACQLRPSMREVVNVLVRMDK*
>Bradi3g28380
MPTPAAVFAGLLLLVLTSGAANAATGPEAKALLAWKASLGNPPALSTWAESSGSVCAGWRGVSCDATG
RVTSLRLRGLGLAGRLGPLGTAALRDLATLDL
NGNNLAGGIPSNISLLQSLSTLDLGSNGFDGPIPPQLGDLSGLVDLRLYNNNLSGDVPHQLSRLPRIAHFDL
GSNYLTSLDGFSPMPTVSFLSLYLNNLN
GSFPEFVLGSANVTYLDLSQNALSGTIPDSLPENLAYLNLSTNGFSGRIPASLSKLRKLQDLRIVSNNLTGGIP
DFLGSMSQLRALELGANPLLGGPIPP
VLGQLRLLQHLDLKSAGLDSTIPPQLGNLVNLNYVDLSGNKLTGVLPPALASMRRMREFGISGNKFAGQI
PSALFTNWPELISFQAQENSFTGKIPPELG
KATKLNILYLYSNNLTGSIPAELGELVSLLQLDLSVNSLTGSIPSSFGKLTQLTRLALFFNQLTGALPPEIGNM
TALEILDVNTNHLEGELPAAITSLRN

LKYLALFDNNFSGTIPPDLGKGLSLIDASFANNSFSGELPRRLCDGLALQNFTANRNKFSGTLPPCLKNCTEL
YRVRLEGNHFTGDITEAFGVHPSLVYL
DVSENKLTGRLSSDWGQCVNITLLHMDGNALSGGIPAVFGGMEKLQDLSLAENNLSGGIPSELGRLGLLF
NLNLSHNYISGPIPENLGNISKLQKVDLSG
NSLTGTIPVGIGKLSALIFLDLSKNKLSGQIPSELGNLIQLQILLDVSSNSLSGPIPSNLDKLRTLQKLNLSRNEL
SGSIPAGFSSMSSLEAVDFSYNRL
TGKIPSGNNIFQNTSADAYIGNLGLCGNVQGVAPCDLNSGSASSGHRRRIVIATVVVVVGVVLLAAVAAC
LILMCRRRPCEHKVLEANTNDAFESMIWEK
EGKFTFFDIMNATDNFNETFCIGKGGFGTVYRAELASGQVVAVKRFHVAETGDISDVSKKSFENEIKALTE
VRHRNIVKLHGFCTSGDYMYLVYECLERG
SLAKTLYGEEGKKNLDWDVRMKVIQGVAHALAYLHHDCNPPIVHRDITLNNILLESDFEPRLCDFGTAKLL
GSASTNWTSVAGSYGYMAPELAYTMRVTE
KCDVYSFGVVALEVMMGKHPGDLLTSLPAISSSQQDDLLLKDILDQRLDPPKEQLAEEVVFIVRIALACTRV
NPESRPTMRSVAQEISAHTQAYLSEAFK
LITISKLTDYQK*
>Bradi4g28107
MAALFLALLLLSLRTAAPYDAGSLLAAKRKLSDPAGALSGWKARSGGHSPCAWPHVACAVNSTTDVAGL
YLKNVSLSGVFPASLCSLRSLRHLDLSQNDI
GGPLPVCLAALPALAYLDLSGNNFSGHVPAAYGAGFRSLATLNLVENALSGAFPAFLANLTSLQELMLGY
NDFTPSPLPENLGDLAGLRLLYLSRCYLKG
RIPSSLGNLRNLVNLDMSVNGLSGEIPGSIGNLGSAVQIEFYSNQLSGRIPEGLGRLKKLQFLDLSMNLLSG
AMPEDAFAGPRLESVHIYQNNLSGRLPA
SLASAPRLNDLRLFGNQIEGPFPPEFGKNTPLQFLDMSDNRLSGPIPPTLCASGRLAEIMLLNNKLEGSIPV
ELGQCWSLTRIRLLNNSLSGTVPPEFWA
LPNVRMLELRLNALSGTIDPAIGGARNLSKLLLQDNRFTGALPAELGNLAILKELFVSGNNLSGPLPASLVEL
SELYTIDLSNNSLSGEIPRDIGRLKKL
VQVRLSHNHLTGVIPPELGEIDGISVLDLSHNELSGGVPGQLQKLRIGNLNLSYNKLTGPLPDLFTNGAWY
NNSFLGNPGLCNRTCPSNGSSDAARRARI
QSVASILAVSAVILLIGFTWFGYKYSSYKRRAAEIDRENSRWVFTSFHKVEFDEKDIVNSLDEKNVIGEGAA
GKVYKAVVGRRSELALAVKKLWPSNTVS
TKMDTFEAEVATLSKVRHRNIVKLFCSMANSTCRLLIYEYMPNGSLGDFLHSAKAGILDWPTRFKIAVHAA
EGLSYLHHDCVPSILHRDVKSNNILLDAD
FGAKVADFGVAKAIVDGTATMSVVAGSCGYIAPEYAYTIHVTEKSDVYSFGVVILELVTGKWPMASEIGEK
DLVAWVRDTVEQNGVESVLDQKLDSLFKD
EMHKVLHIGLMCVNIVPNNRPPMRSVVKMLLDVEEENKRKARIEASLPSI*
>Bradi5g15070
MGPGTASASWGLQLGVVLVFLLASGSQGLNHEGWLLLALKSQMIDSSHHLDNWKPRDPSPCMWTGVI
CSSAPMPAVVSLNLSNMELSGTVGQSIGGLAEL
TDLDLSFNEFFGTIPTGIGNCSKLVWLALNNNNFEGTIPPELGKLAMLTTCNLCNNKLYGSIPDEIGNMASL
VDLVGYSNNISGSIPHSIGKLKNLQSIR
LGQNLISGNIPVEIGECHNLVVFGLAQNKLQGPLPKEIGNLSLMTDLILWGNQLSGAIPPEIGNCTNLRTIA
LYDNGLVGPIPPTIGNIKYLQRLYLYRN
SLNGTIPPEIGNLLLAGEIDFSENFLMGGIPKELGNIPGLYLLYLFQNQLTGFIPKELCGLKNLTKLDLSINSLT
GPIPAGFQYMPKLIQLQLFNNRLSG
DIPPRFGIYSRLWVVDFSNNNITGQIPRDLCRQSNLILLNLMSNKLSGNIPHRITSCRSLVQLRLSDNSLTGS
FPTDLCNLVNLTTIELARNKFNGPIPP

QIGNCMALQRLDLTNNYFTSELPREIGNLSKLVVFNISSNRLGGSIPLEIFNCTMLQRLDLSQNSLEGSLPTE
VGRLPQLELLSFADNRLSGQVPPILGK
LSHLTALQIGGNQFSGGIPKELGLLSSLQIAMNLSYNNLSGNIPSELGSLALLENLFLNNNKLTGAIPDTFAN
LSSLLELNVSYNNLTGALPPVPLFDNM
VVTSFIGNRGLCGGQLGKCGSESPSSSQSSNSVSRPMGKIIAIVAAIIGGISLILIAILLHQMRKPRETIAPLQ
DKQILSAGSNMPVSAKDAYTFQELVS
ATNNFDESCVIGRGACGTVYRAILKPGHIIAVKKLASNREGSNTDNSFRAEILTLGKIRHRNIVKLYGFIYHQ
GSNLLLYEYMSRGSLGELLHGQSSSSL
DWDTRFMIALGAAEGLSYLHHDCKPRIIHRDIKSNNILLDENFEAHVGDFGLAKVIDMPYSKSMSAIAGSY
GYIAPEYAYTMKVTEKCDIYSYGVVLLEL
LTGRAPVQPIELGGDLVTWAKNYIRDNSVGPGILDRNLDLEDKAAVDHMIEVLKIALLCSNLSPYDRPPM
RHVIVMLSESKDRAQTSSASSPASDNSSKK
DSS*
