# Supplementary material for: BdERECTA controls vasculature patterning and phloem-xylem organization in Brachypodium distachyon
Source: BMC Plant Biol. 2021 Apr 23;21:196. doi: 10.1186/s12870-021-02970-2 (PMC8067424; doi:10.1186/s12870-021-02970-2)
Supplement: Supplementary file 7 — Additional file 7: Figure S1. Surface area of the eight largest vascular bundles along the fifth node in the main Brachypodium stem. Figure S2. Mutation loci and expression of Bradi1g46450 in WT, vasc1–1 and vasc1–2 .Figure S3. Laser capture microdissection. Figure S4. Validation of LCM data. Figure S5. Picture of WT and mutants at 20 DAG. Figure S6. Full length gels [file 12870_2021_2970_MOESM7_ESM.pdf]

Figure S1

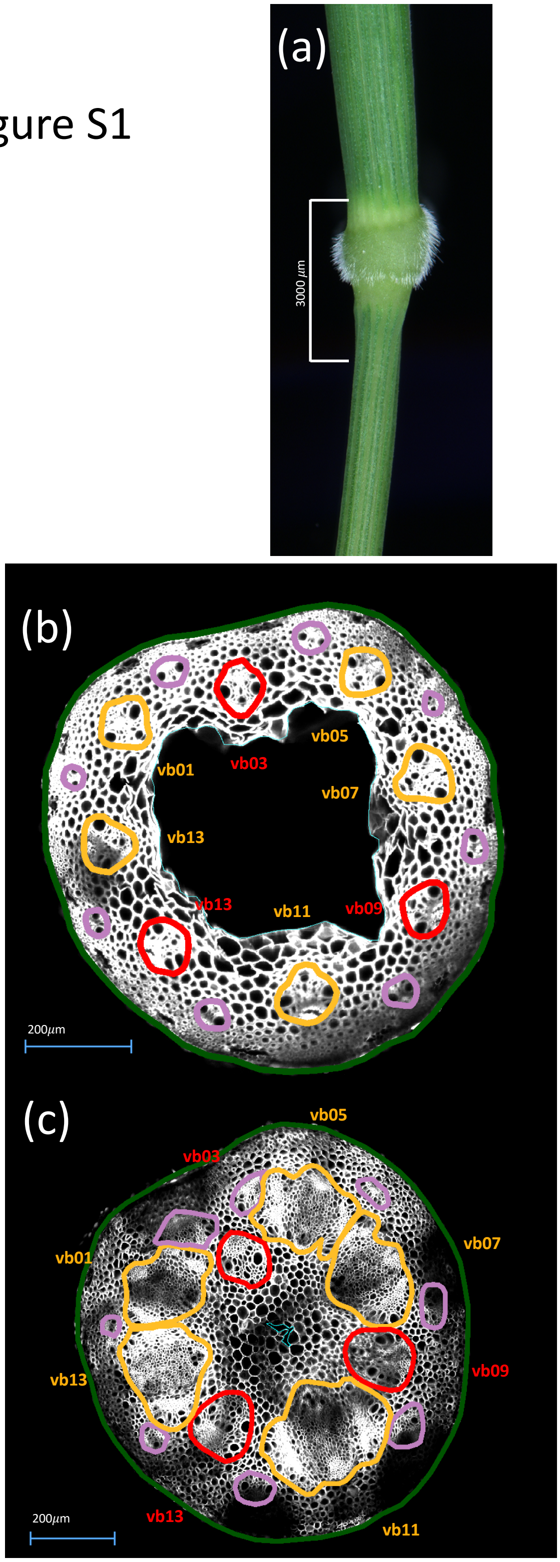

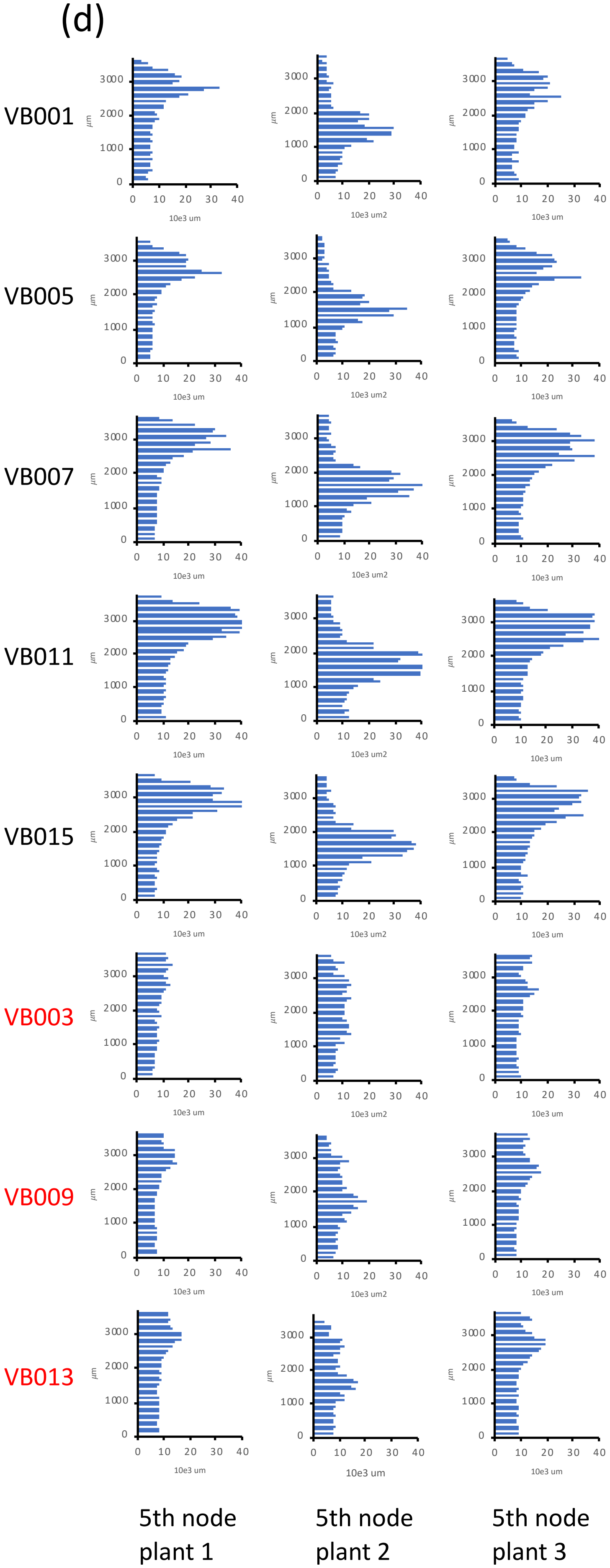

Figure S1. Surface area of the eight largest vascular bundles along the fifth node in the main Brachypodium stem.

(a) studied node area. (b) cross section at the base of the node (at 0.1 mm, plant 2) acquired with confocal microscopy. Position and name of vascular bundles are shown.

(c), cross section (at 1.5 mm, plant 2) acquired with confocal microscopy. VB identified in (b) have an enlarged shape at this position. (d), thickness of VB all along the node area. VB003, VB009, VB13 show reduced enlargement in the node compared with VB001, VB005, VB007, VB011, VB015.

Figure S2

(a)

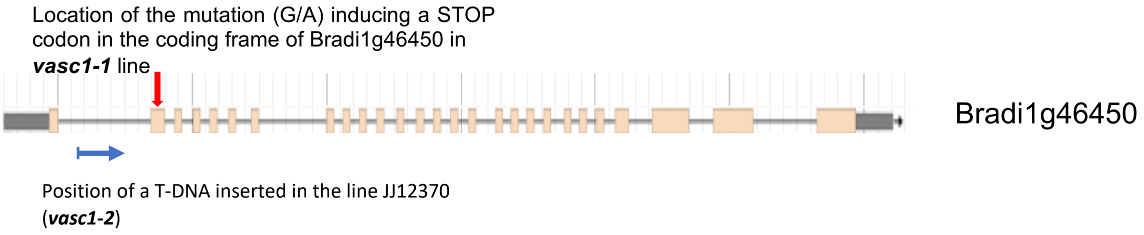

(b)

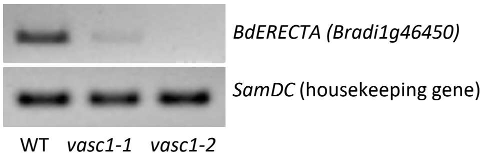

Figure S2. Mutation loci and *ERECTA* expression in mutants.

(a) Position of chemically induced mutation (red arrow) and T-DNA (blue arrow) in Bradi1g46450 of *vasc1-1* and *vasc1-2* respectively. Orange squares represent exons. (b) RT-PCR analysis. *BdERECTA* transcript level is decreased in *vasc1-1* and not detected in *vasc1-2* compared to WT.

Figure S3

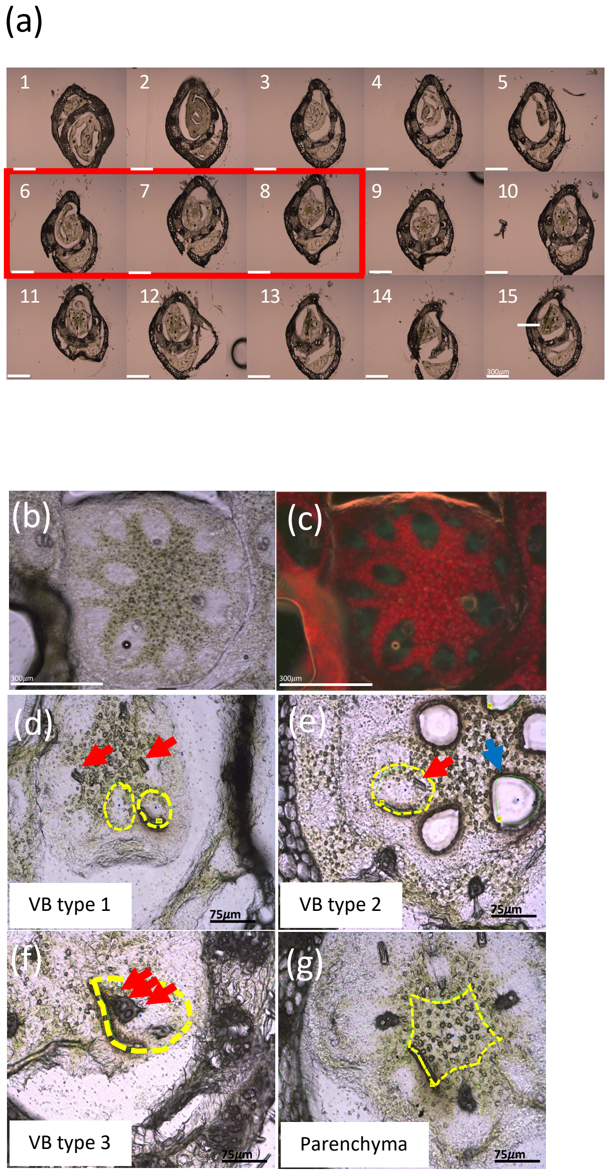

Figure S3. Laser capture microdissection (LCM).

(a) serial cross cryo-sections from coiled leaves (1 to 5), top part of meristem (6 to 8) and lower part of meristem (9 to 15). Red frame shows the selected sections for LCM. Bar = 300 μm. (b) cross section of meristem under light microscopy. (c) cross section of meristem under UV microscopy. (d) dotted yellow areas show VB type 1 used for microdissection. (e) dotted yellow area shows VB type 2 with one protoxylem cell. (f) dotted yellow area shows VB type 3 with three protoxylem cell. (g) dotted yellow line shows parenchyma tissue microdissected for control. Red arrows show protoxylem cell. Blue arrow shows microdissected area.

Figure S4

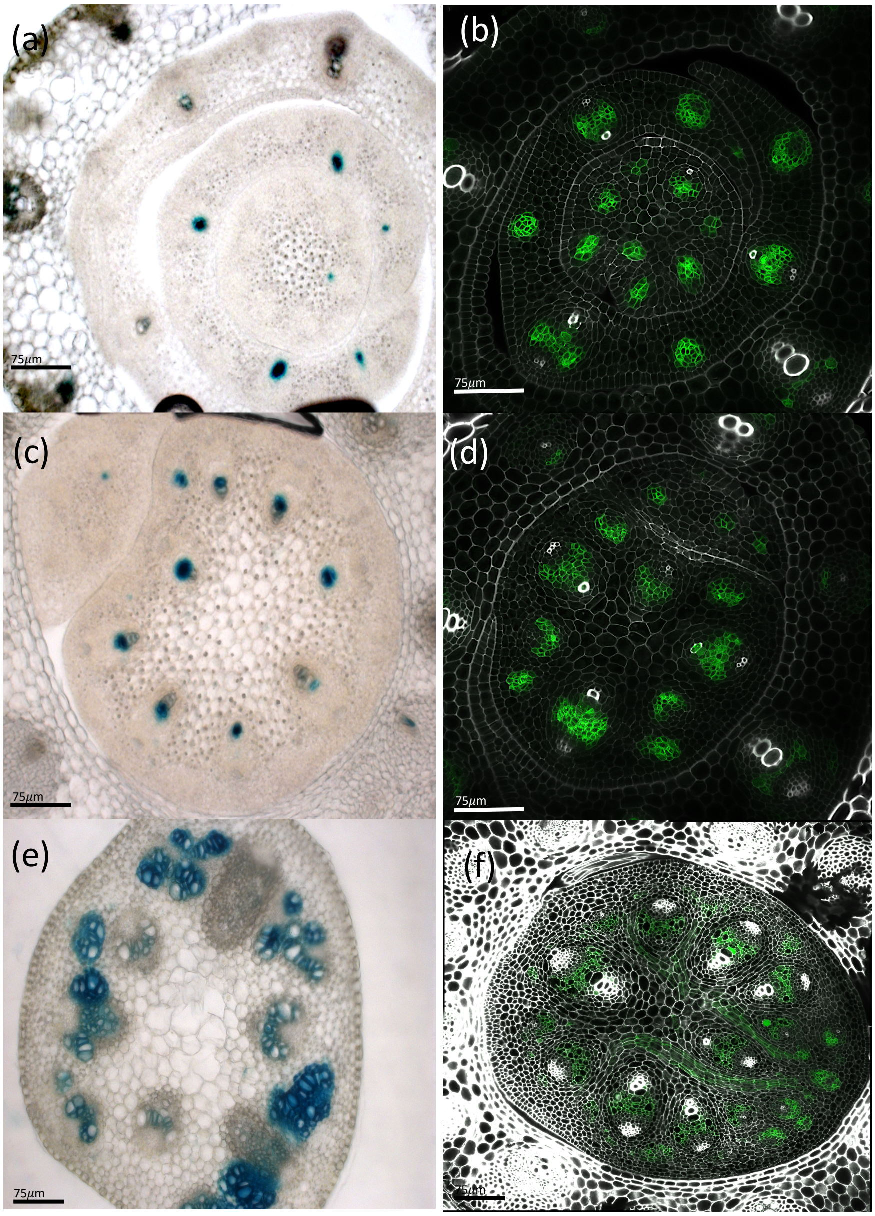

Figure S4. Validation of LCM data.
(a), (c), (e) expression profile of XCP1:GUS (Valdivia *et al.*, 2013). (b), (d), (f), expression profile of PIN1a:Citrine (O'Connor *et al.*, 2014b).
(a) and (b), section in shoot apical meristem. (c), (d), section in a zone located below the apical meristem. (e), (f), section in the node area.

Figure S5

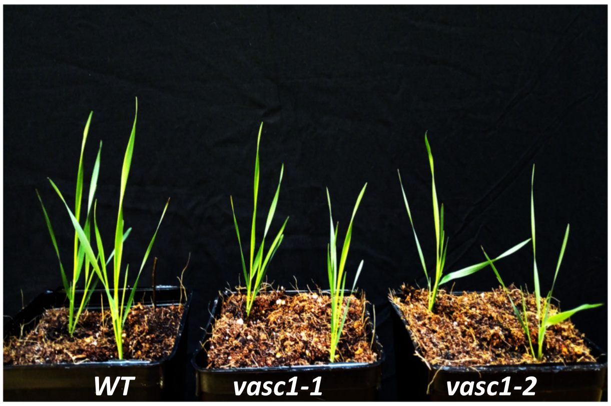

Figure S5. Picture of WT and mutants at 20 DAG.

Figure S6

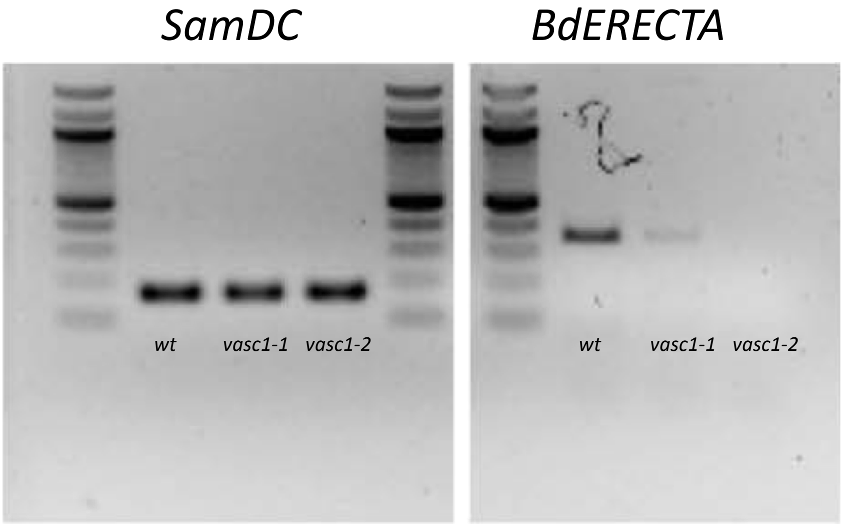

Figure S6. Full-length gels of cropped-gel images (displayed in figure S2). A 100 base pairs ladder was used to verify amplicon sizes.
